# Structure Merging Approach Leads to New Dual Potent and Selective USP25/USP28 Inhibitors

**DOI:** 10.1021/acs.jmedchem.5c03045

**Published:** 2026-04-22

**Authors:** Victor Hernandez-Olmos, Jonathan Vincent Patzke, Caroline E. Stone, Radhika Karal Nair, Kathrin Weller, Florian Sauer, Erik Endres, Oumaima Jamai, Lea Rachor, Cornelia H. Warmutz, Martin P. Schwalm, Marko Mitrovic, Vincent Grenier, Johanna H. M. Ehrler, Anna Proschak, Jan Heering, Christoph Sotriffer, Monique P. C. Mulder, Stefan Knapp, Caroline Kisker, Ewgenij Proschak

**Affiliations:** † 587462 Fraunhofer Institute for Translational Medicine and Pharmacology ITMP, Theodor-Stern-Kai 7, 60596 Frankfurt am Main, Germany; ‡ Fraunhofer Cluster of Excellence Immune-Mediated Diseases CIMD, Theodor-Stern-Kai 7, 60596 Frankfurt am Main, Germany; § Rudolf Virchow Center for Integrative and Translational Bioimaging, Institute for Structural Biology, 9190 Julius-Maximilians-University Würzburg, 97080 Würzburg, Germany; ∥ Department of Cell and Chemical Biology, 4501 Leiden University Medical Centre, Einthovenweg 20, 2300 RC Leiden, The Netherlands; ⊥ Institute of Pharmacy and Food Chemistry, Julius-Maximilians-University Würzburg, 97074 Würzburg, Germany; # Institute of Pharmaceutical Chemistry, 9173 Goethe University Frankfurt, 60438 Frankfurt am Main, Germany; ¶ Structural Genomics Consortium (SGC), Buchmann Institute for Molecular Life Sciences (BMLS), 60438 Frankfurt am Main, Germany

## Abstract

USP25 and USP28 are critical deubiquitylases (DUBs) that have been implicated in various diseases, particularly cancer and cardiac dysfunction. Several small-molecule inhibitors have been reported, exhibiting dual inhibitory activity in the low micromolar range. In this study, we present a strategy that merges structural features of the previously identified inhibitors AZ1 and vismodegib to develop a new class of potent dual inhibitors. Several of these newly synthesized compounds exhibit high potency across multiple orthogonal assays and demonstrate excellent selectivity over other ubiquitin-specific proteases. Moreover, a suitable negative control has also been identified, supporting the validity of the observed effects in cellular assays. These results highlight the potential of these compounds to serve as advanced chemical probes for dual USP25/USP28 inhibition and as candidates for further therapeutic development.

## Graphical abstract

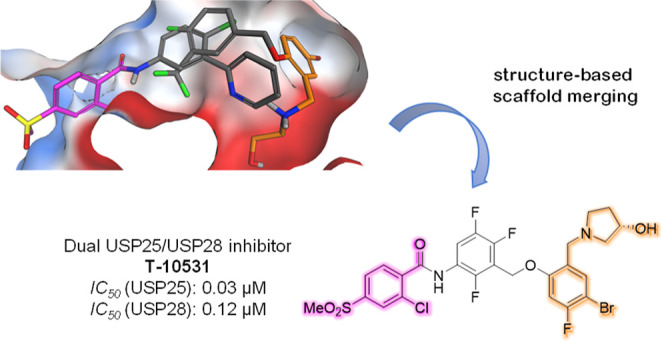

## Introduction

Deubiquitylases (DUBs) define a class of more than 100 human enzymes that are divided into seven families and play a crucial role in cellular regulation by removing ubiquitin (Ub) from substrates, influencing processes such as protein degradation and activity control.^1^ ^,^ ^2^ ^,^ ^3^ By targeting specific substrates, DUBs can either suppress or completely reverse the effects of ubiquitylation, making them vital regulators of numerous cellular processes in eukaryotic cells.^4^ ^,^ ^5^ Dysregulation of DUB expression or function is often linked to the development and progression of various diseases.^2^ ^,^ ^6^ ^,^ ^7^

Ubiquitin-specific proteases (USPs) are cysteine proteases and represent the largest family among the seven known human DUB families.^8^ They are of significant interest as potential drug targets for therapeutic interventions because many USPs regulate key cellular pathways and are often overexpressed or display heightened activity in various cancerous and noncancerous conditions.^8^ However, their evolutionary similarity and wide range of biological functions pose a major challenge for developing selective small-molecule inhibitors. As a result, drug screening efforts aimed at targeting a single USP have frequently produced bi- or polyspecific inhibitors that lack the desired selectivity.^2,9,10^

USP28 and USP25 are closely related members of the USP family, characterized by identical domain architectures and a high degree of sequence similarity, particularly in their central catalytic domains, which share 57% identity.^11^ Despite these similarities, they exhibit distinct biological functions and notably different catalytic activities.

USP28 plays a key role in regulating cell proliferation,^12^ differentiation,^13^ DNA damage repair,^14^ genome maintenance,^15^ and apoptosis by stabilizing specific nuclear proteins.^16^ It targets oncogenic factors such as c-Myc, HIF1α, ΔNp63, LSD1, and RecQ5, linking it to cancers like squamous cell carcinoma, colorectal, gastric, and triple-negative breast cancer.^16^ Interestingly, USP28 also stabilizes p53 in melanoma models, suggesting a dual role as both an oncogene and a potential tumor suppressor.^16^ ^,^ ^17^ Additionally, USP28 inhibition has also been associated with cardiac dysfunction in the diabetic heart.^18^

Similarly, USP25 acts as a tumor promoter in Wnt-dependent cancers by positively regulating Wnt signaling through the stabilization of tankyrases.^19^ It also plays a crucial role in the metabolic reprogramming of pancreatic cancer cells within the tumor core by stabilizing the HIF1α transcription factor.^20^ Beyond these roles, USP25 is involved in various cell-type and tissue-specific pathways, including insulin-dependent glucose metabolism,^21^ the regulation of immune cell activity both positively and negatively,^22^ ^,^ ^23^ and endoplasmic reticulum-associated degradation.^24^ Recently, USP25 modulation has also been associated with a potential treatment for cardiac dysfunction^25^ and Alzheimer’s disease.^26^

High-throughput screening efforts have identified several potent small-molecule inhibitors targeting USP25 and USP28.^27^ ^,^ ^28^ ^,^ ^29^ ^,^ ^30^ Among these, two compounds stand out as the most studied in human and animal models of cancer and other diseases: AZ1 ^31^ and vismodegib (VSM),^32^ for which Patzke et al. have recently reported the crystal structures in complex with USP28 ^33^ (Figure 1).

**Figure 1:**
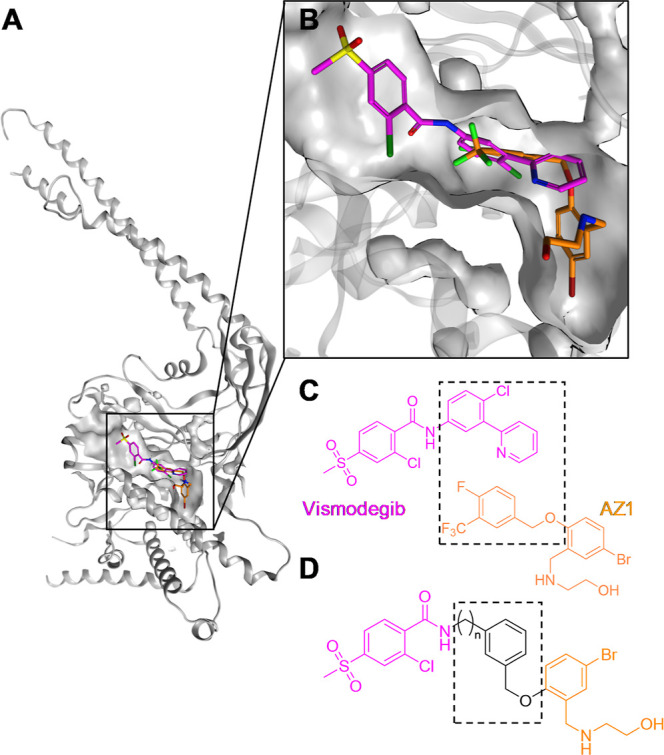
Chemical structures and crystal structure of AZ1 and VSM in complex with USP28. (A) Crystal structure of AZ1 and vismodegib with USP28. Gray cartoon shows the USP28 structure in complex with AZ1 (PDB ID: 8P1P). (B) Close-up of the binding pocket with VSM (pink) and AZ1 (orange). (C) VSM and AZ1 structures. (D) Proposed merged structure.

The superimposition of the structure of USP28 in complex with VSM (PDB ID: 8P14) and AZ1 (PDB ID: 8P1P) revealed that both compounds’ binding sites overlap partially (Figure 1A,B).^33^ The 2-phenylpyridine moiety of VSM superimposes with the benzyloxy group of AZ1 (Figure 1C). We therefore envisioned that a merged structure of these two inhibitors could result in a significantly more potent compound, which led to the design of merged compounds bridging the benzamide and the phenyl moiety by a benzyloxy linker of varied length (Figure 1D).

## Results and Discussion

### Chemistry

First, the length of the linker between the sulfone-containing aromatic ring and the central aromatic ring was studied to explore the SARs of the selected scaffold. Synthesis of the compounds was accomplished in 4 steps, as displayed in Scheme 1. In the first step, an amide coupling between acid chloride **1** and 2 equiv of aminoalcohols **2–4** took place quantitatively in DCM without the need for the presence of an additional base. This approach was chosen as we discovered that adding bases led to undesired side reactions at the alcohol group. The resulting benzylic alcohol was converted to the corresponding chloride or bromide by treatment with thionyl chloride or phosphorus tribromide, respectively. Subsequently, a nucleophilic substitution between **8**–**10** and 5-bromosalicylaldehyde as nucleophile in the presence of potassium carbonate in DMF led to the intermediates **11**–**13**. Finally, a reductive amination reaction using ethanolamine and acetic acid in DMF, followed by the addition of sodium cyanoborohydride, provided final compounds **14**–**16**.

**Scheme 1:**
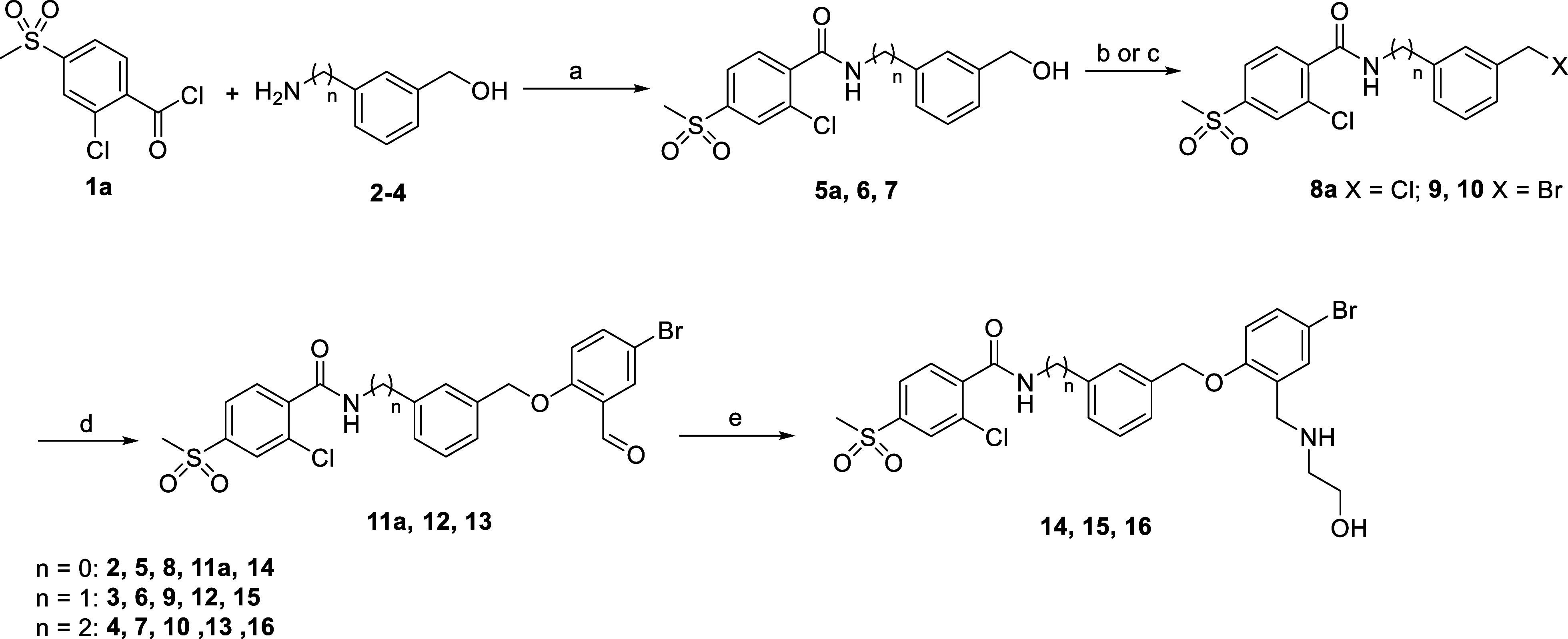
Synthetic Procedure Used for the Synthesis of Compounds **14, 15**, and **16** ^a^

a Conditions. (a) DCM, rt, 52–97%; (b) SOCl_2_, dichloroethane, 40 °C, quantitative; (c) PBr_3_, THF/DCM, 97–98%; (d) 5-bromosalicylaldehyde, K_2_CO_3_, DMF, 60 °C; (e) 1. ethanolamine, acetic acid, DMF 2. NaBH_3_CN, 30–46%.

Next, based on the scaffold of compound **14**, we prepared a series of derivatives as displayed in Scheme 2 where we studied the effect of different substitutions around the molecule using the same synthetic route as described in Scheme 1.

**Scheme 2:**
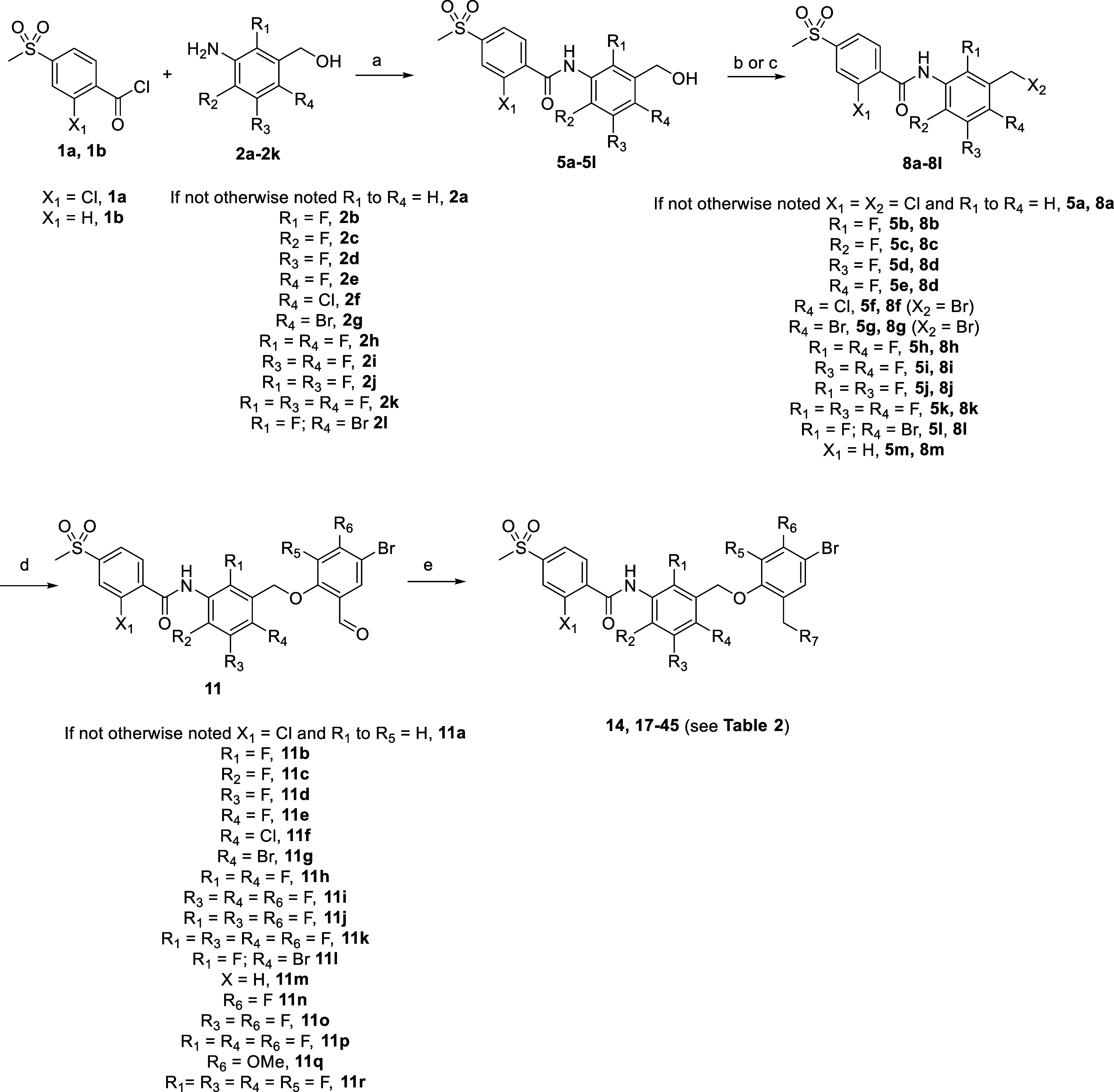
Synthetic Procedure Used for the Synthesis of Compounds **14** and **17–45** ^a^

a Conditions. (a) DCM, rt, 35%, quantitative; (b) SOCl_2_, dichloroethane, 40 °C, 50%, quantitative; (c) PBr_3_, THF/DCM, quantitative; (d) corresponding salicylaldehyde, K_2_CO_3_, DMF, 60 °C, 6.1–74%; (e) 1. corresponding amine, acetic acid, DMF 2. NaBH_3_CN or NaBH­(OAc)_3_, 16–82%.

Compound **50** (Scheme 3), with a *para*-substituted central aromatic ring, was also prepared using the same synthetic route as described in Scheme 2.

**Scheme 3:**
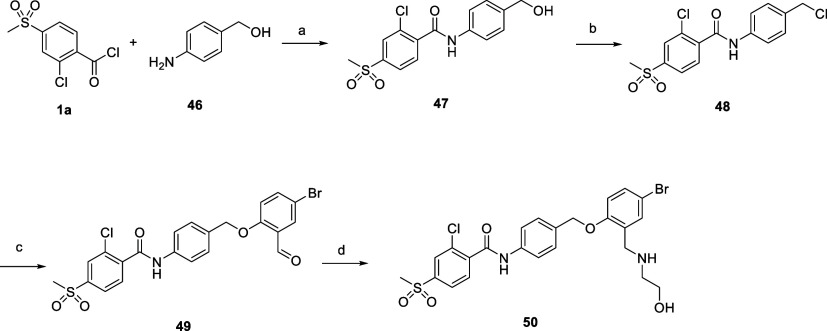
Synthetic Procedure Used for the Synthesis of Compound **50** ^a^

a Conditions. (a) DCM, rt, 83%; (b) SOCl_2_, dichloroethane, 40 °C, quantitative; (c) 5-bromosalicylaldehyde, K_2_CO_3_, DMF, 60 °C, 54%; (d) 1. ethanolamine, acetic acid, DMF 2. NaBH_3_CN, 21%.

Derivatives with inverse amide configuration (**55a** and **55b**) with *para* and *meta* substitution, were prepared in 3 steps according to Scheme 4. In the first step, an amide coupling reaction between aniline **51** and the acid chlorides **52a** or **52b** took place in DCM in the presence of triethylamine as a base. The intermediate **53** was then directly subjected to the final two steps of the synthesis route described in Scheme 2 to obtain the final compounds **55a** and **55b**.

**Scheme 4:**
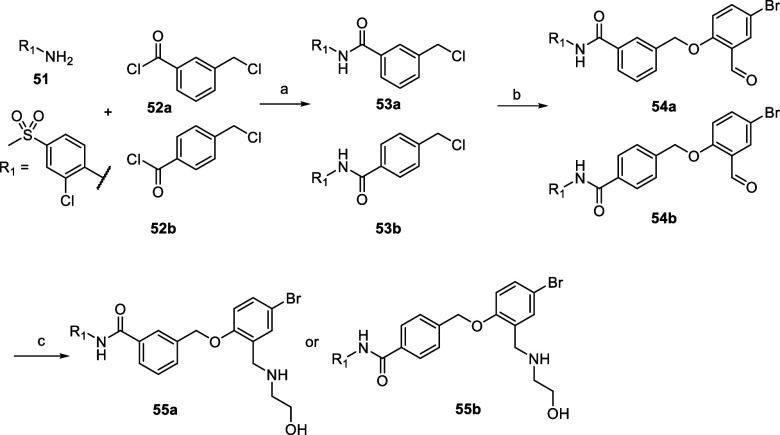
Synthetic Procedure Used for the Synthesis of Compounds **55a** and **55b** ^a^

a Conditions. (a) DCM, Et_3_N, rt, 35–69%; (b) 5-bromosalicylaldehyde, K_2_CO_3_, DMF, 60 °C, 71–84%; (c) 1. ethanolamine, acetic acid, DMF 2. NaBH_3_CN, 31–33%.

A further series of derivatives where the 2-chloro-4-methylsulfone benzamide was exchanged by either smaller groups like methyl or trifluoromethyl or by a bioisosteric replacement of the amide bond with a heterocycle was prepared according to Scheme 5. Some of the starting chlorides were commercially available, and some had to be prepared from the corresponding alcohols (see Supporting Information). The last two steps of the synthesis, leading to intermediates **57a**–**57j** and final compounds **58a**–**58j**, were accomplished in the usual conditions as reported before.

**Scheme 5:**
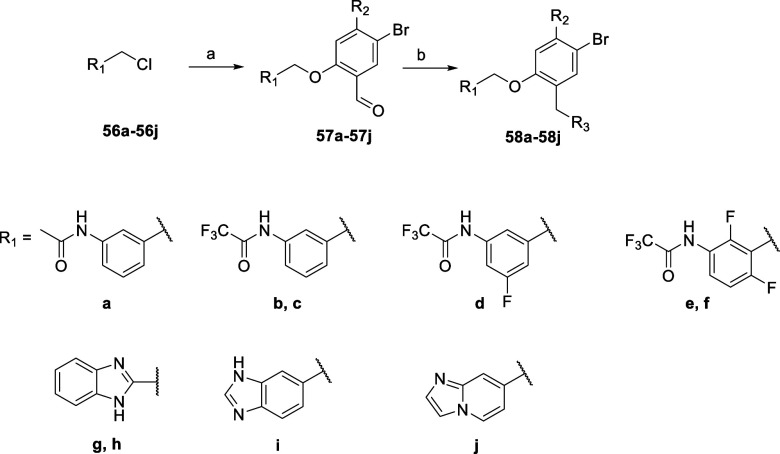
Synthetic Procedure Used for the Synthesis of Compounds **58a**–**58g** ^a^

a Conditions: (a) 5-Bromosalicylaldehyde or 5-bromo-4-fluoro-2-hydroxybenzaldehyde, K_2_CO_3_, DMF, 60 °C, 24–88%; (b) 1. amine, acetic acid, DMF 2. NaBH_3_CN, 19–72%.

The synthesis of the fluorescent ligand (tracer) used in the NanoBRET target engagement assay was accomplished in three steps from **11p** (Scheme 6). In the first step, the usual reductive amination conditions using BocNH-PEG2-CH_2_CH_2_NH_2_ as amine provided intermediate **60** in good yield. Then, *N*-Boc deprotection with trifluoroacetic acid followed by amide formation by reaction of **60** with the Py-BODIPY-NHS ester produced the fluorescent compound **61**.

**Scheme 6:**
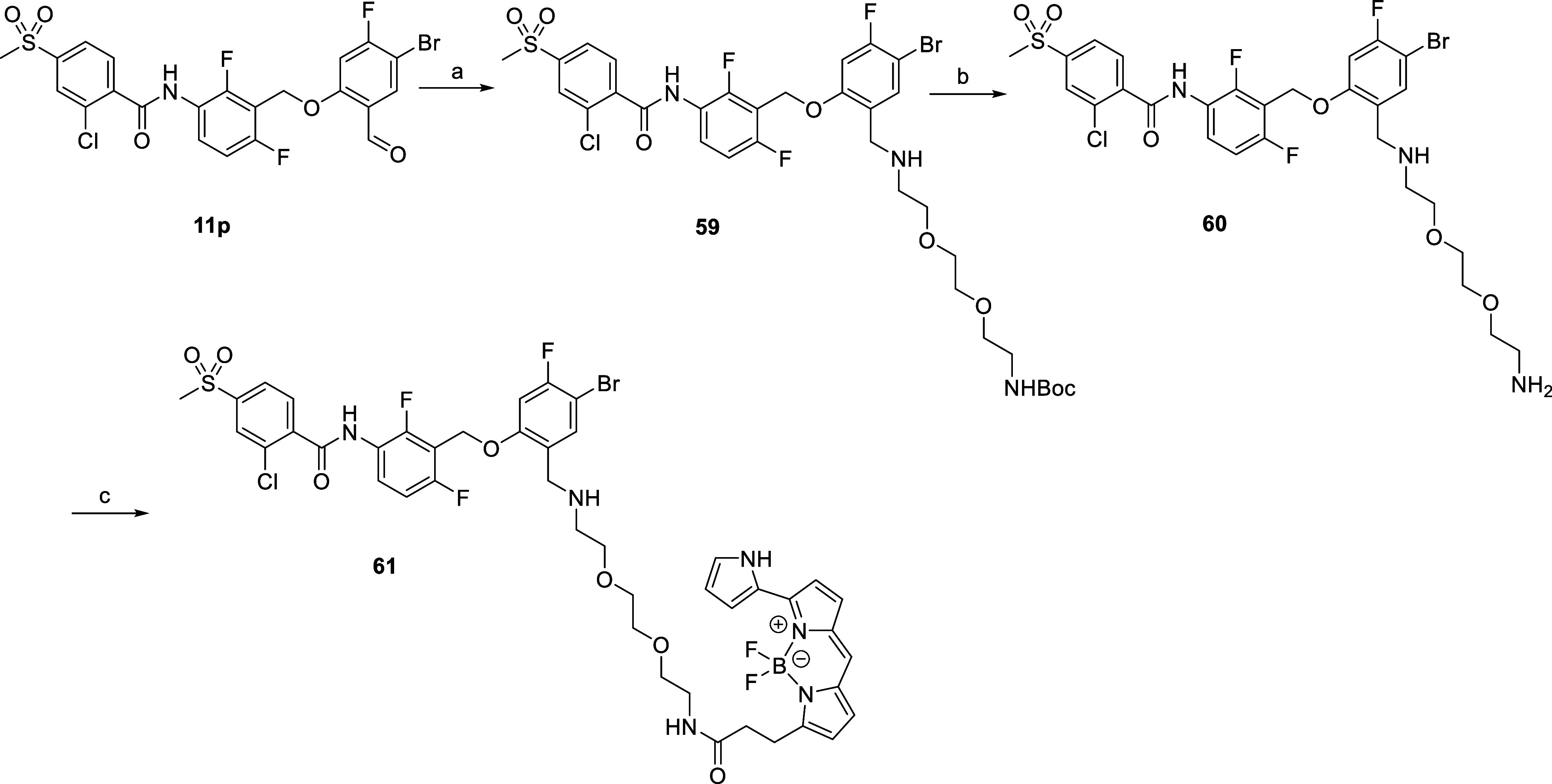
Synthetic Procedure Used for the Synthesis of Compound **60** ^a^

a Conditions. (a) 1. BocNH-PEG2-CH_2_CH_2_NH_2_, acetic acid, DMF 2. NaBH_3_CN, 85%; (b) TFA, DCM, quantitative; (c) Py-BODIPY-NHS ester, DIPEA, DMF, 43%.

Biotin conjugate **62** was prepared, similarly to fluorescent compound **61**, by reaction of intermediate **60** with NHS-biotin (Scheme 7).

**Scheme 7:**
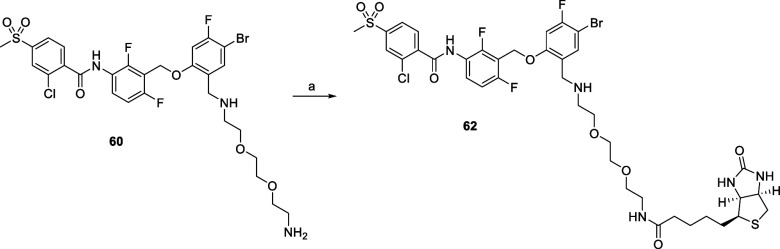
Synthetic Procedure Used for the Synthesis of Compound **62** ^a^

a Conditions. (a) NHS-biotin, Et_3_N, DCM (25%).

### SAR Studies

The inhibitory potencies of the compounds, assessed by a Ub-Rho110 cleavage assay, are shown in Table 1. From the three compounds initially prepared, **14** and **16,** with linkers *n* = 0 and *n* = 2, respectively, displayed higher potencies than **15**. Due to the more abundant availability of 3-aminobenzyl alcohol derivatives in comparison to 3-(2-aminoethyl)­benzyl alcohols, a linker with *n* = 0 as in **14** was selected as the optimal one for further studies. Compound **50** with a *para* configuration resulted in a weaker inhibitor than the corresponding *meta* compound (**14**), while the compounds with an inverse amide (**55a**–**55b**) were weak or inactive.

**Table 1:** IC_50_ Results for Compounds **14**–**16, 49, 55a**, and **55b**

cpd	IC_50_ (μM) USP25 ± SD	IC_50_ (μM) USP28 ± SD
**AZ1**	1.08 ± 0.06	1.76 ± 0.13
**VSM**	2.92 ± 0.29	3.51 ± 0.12
**14**	2.68 ± 0.08	10.4 ± 1.3
**15**	16.7 ± 1.7	70.5 ± 28.0
**16**	4.94 ± 0.85	5.25 ± 0.96
**50**	9.33 ± 2.87	32.2 ± 5.0
**55a**	23.5 ± 1.0	45.5 ± 7.6
**55b**	>100	13.8 ± 3.4

We proposed the binding mode of compound **14** in complex with USP28 using molecular modeling (Figure 2). Based on the previously shown superposition of VSM (PDB ID: 8P14) and AZ1 (PDB ID: 8P1P), we employed the Builder panel in Molecular Operating Environment (MOE 2024.0601) to generate compound **14**, followed by a minimization of the potential energy of USP28 in complex with **14** using the built-in implementation of the Amber12:EHT force field with default settings. The resulting binding mode provided insights for rationalizing the subsequent optimization efforts. Our analysis revealed that the amide moiety forms directed interactions with C644 and E366. A preliminary SAR study of vismodegib derivatives as USP28 inhibitors, reported by Zhou et al.,^35^ demonstrated a preference for the methylsulfone substituent on the benzamide moiety, which we retained in our design. However, the influence of the Cl-substituent still requires further investigation. The 3-aminobenzyloxy linker is engaged in lipophilic interactions with the methyl groups of T364 and L264. We hypothesized that these interactions could be enhanced by reducing the electron density of the central aromatic moiety. This hypothesis is supported by the observations made by Wrigley et al.,^32^ who showed that the derivative of AZ1 lacking the trifluoromethoxy, trifluoromethyl, and/or fluorine substituents exhibited reduced activity. Similar interactions between L264 and the 2-bromophenoxy substituent were observed, further suggesting that this moiety might benefit from substitution with electron-withdrawing groups. The 2-amino-ethanol moiety participates in a hydrogen bond network with Q315 and D265, as described for AZ1.^34^ While maintaining these interactions is advantageous, reducing the flexibility of the 2-amino-ethanol moiety by rigidization might result in increased compound potencies.

**Figure 2:**
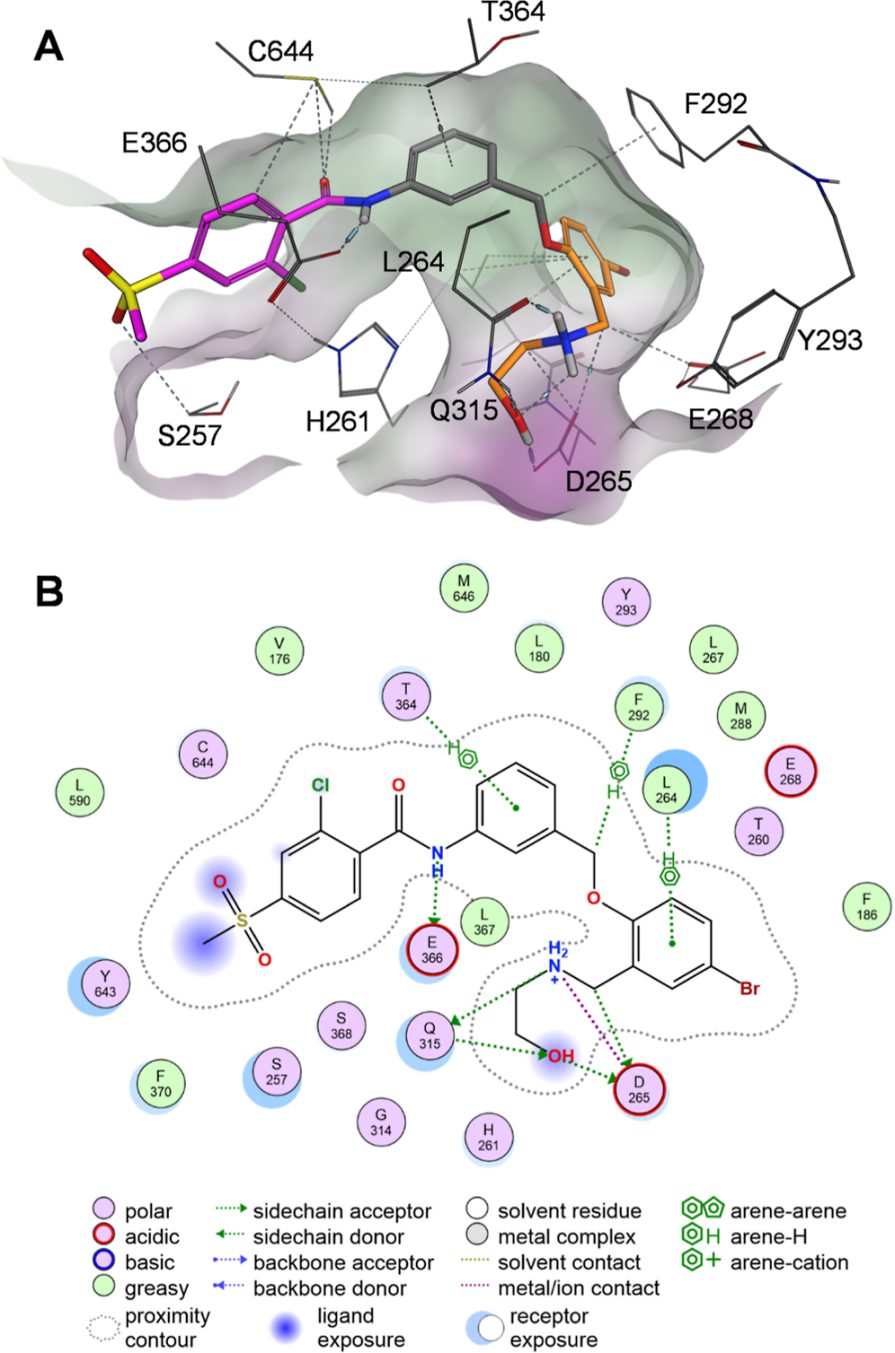
Proposed binding mode of compound **14** in complex with USP28 in (A) 3D and (B) 2D.

The IC_50_ values for this series of new compounds are summarized in Table 2. Fluorine atoms were systematically introduced in positions R_1_ to R_4_ (compounds **17**–**20**). The resulting inhibitors were more potent than compound **14**, excluding compound **18,** which was completely inactive, indicating that substitution R_2_ = F is not tolerated. Chlorine and bromine substitutions in R_4_ were also produced (compounds **21**–**22**). The chlorine-substituted compound (**21**) resulted in a comparably active inhibitor to **14**, but bromine at this position (**22**) was not tolerated. Different rigidification options for the aminoethanol moiety were explored (compounds **23**–**27** and **34**). Derivatives such as 3-hydroxypyrrolidine (**23** and **24**), 3-hydroxypiperidine (**25** and **26**), or 3-carboxamidepyrrolidine (**34**) were found to be potent inhibitors, whereas the 2-hydroxycyclopentanamine derivative **27** exhibited only poor inhibition. The 3-(*S*)-hydroxypyrrolidine moiety, as in **23**, was chosen as the ideal scaffold for most of the subsequent compounds prepared. The fluorine screening in the three most favorable positions was repeated with the 3-(*S*)-hydroxypyrrolidine moiety, providing compounds **28**–**30**, resulting in comparable or slightly higher potencies than the corresponding aminoethanol derivatives **17**, **18**, and **20**. Additionally, R_6_ = F in the new derivative **31** proved to be a beneficial substituent. In contrast, compound **32**, lacking the chlorine in the sulfone-containing aromatic ring, and compound **33** with R_5_ = OMe displayed weak or no inhibition, respectively.

**Table 2:**
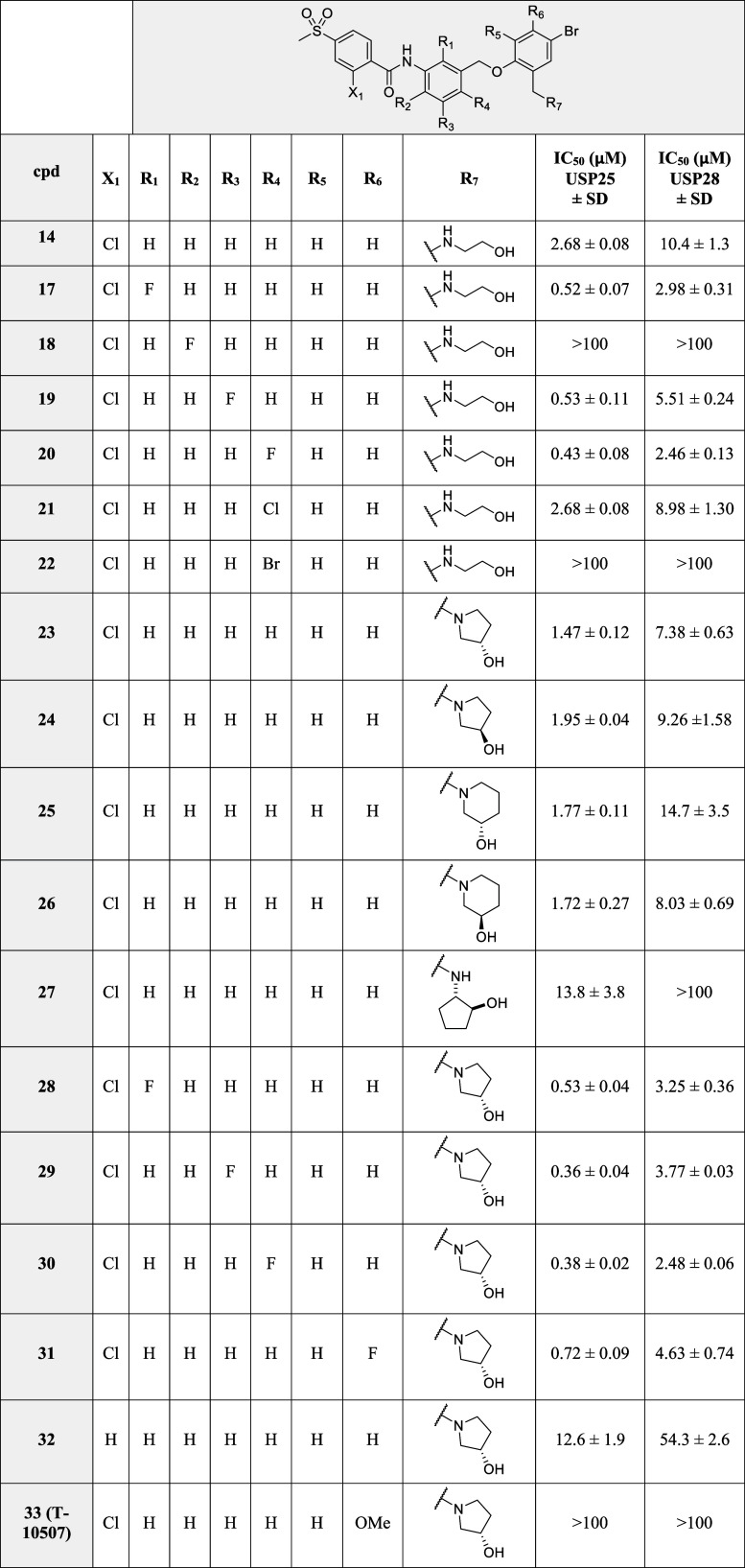

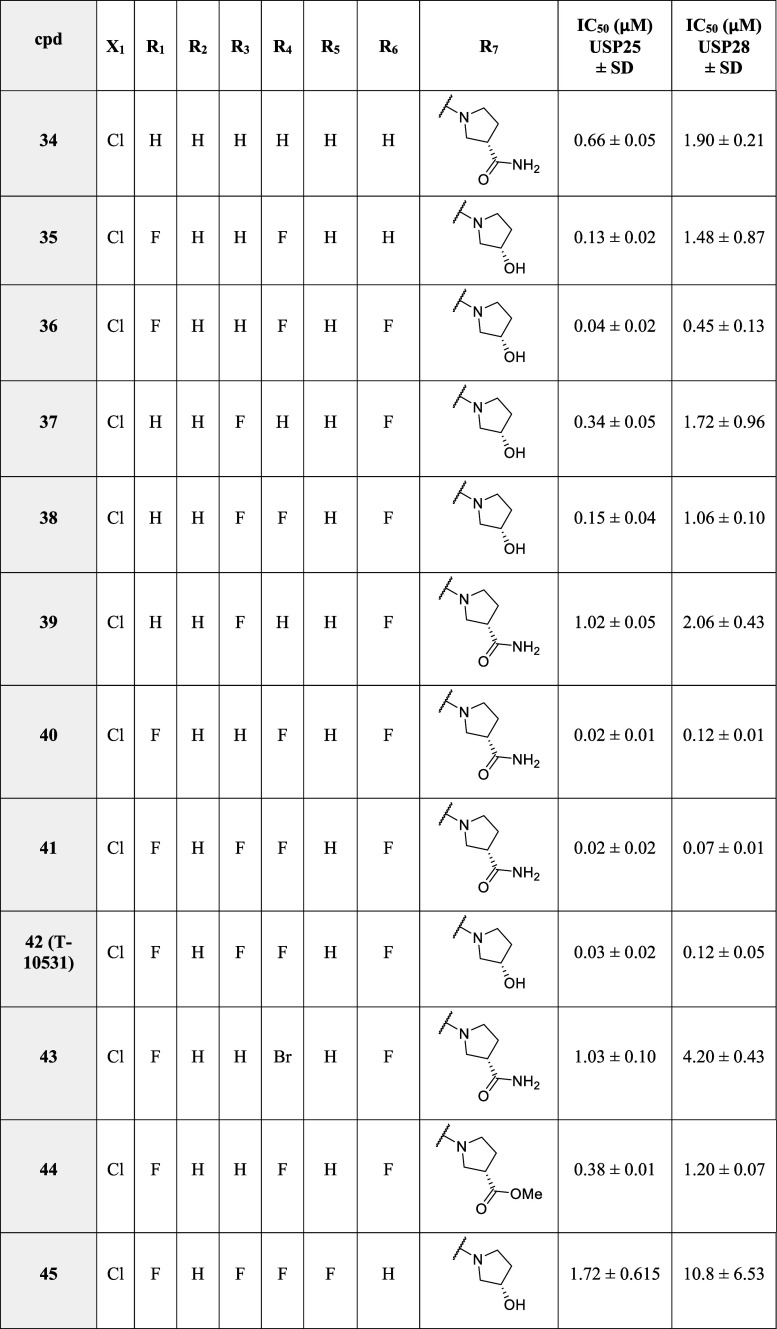
IC_50_ Results for Compounds **14** and **17**–**45**

Next, we combined beneficial features of the developed lead structure, such as fluorine atoms in R_1_, R_3_, R_4_, and R_6_ (compounds **35**–**42**). As expected, this strategy resulted in highly potent inhibitors with IC_50_ values in the low nanomolar range, particularly for compounds comprising 2 or 3 fluorine atoms in the central aromatic ring (compounds **35**, **36**, **38**, and **40**–**42**). The activating effect of the fluorine substituents was also evident in compound **43**, which contains a bromine at position R_4_ and exhibited low micromolar IC_50_ values. In contrast, the corresponding compound without the fluorines (compound **22**) was inactive. Compound **44**, with a methyl ester residue in the pyrrolidine ring, was also synthesized. Subsequent activity assays showed a lower potency of this compound compared to the corresponding hydroxy- or carboxamide-residues (compound **44** vs **36** or **40**). This observation was in accordance with the proposed binding mode, which indicated that hydrogen bond donors are key in that region, which the ester cannot provide. Finally, compound **45**, bearing a fluorine substituent at R_5_, was synthesized and showed markedly weaker activity compared to compound **42**, its R_6_-fluorinated analogue. This comparison highlights a specific and beneficial effect of fluorine substitution at the R_6_ position, as opposed to R_5_.

From all the new scaffolds prepared without the arylsulfone moiety, the trifluoroacyl compounds (**58b**–**58f**) were clearly the most active derivatives with comparable IC_50s_ to the corresponding original arylsulfone derivatives, reaching nanomolar IC_50_ values (Table 3). However, for some of these derivatives, especially with several fluorine atoms in the central aromatic ring (**58e** and **58f**), chemical instability was observed. All other substitutions led to inactive compounds except for **58g**, which had a modest USP25 IC_50_ of 4.68 μM. The SARs of the merged scaffold based on all compounds prepared are summarized in Figure 3.

**Table 3:**
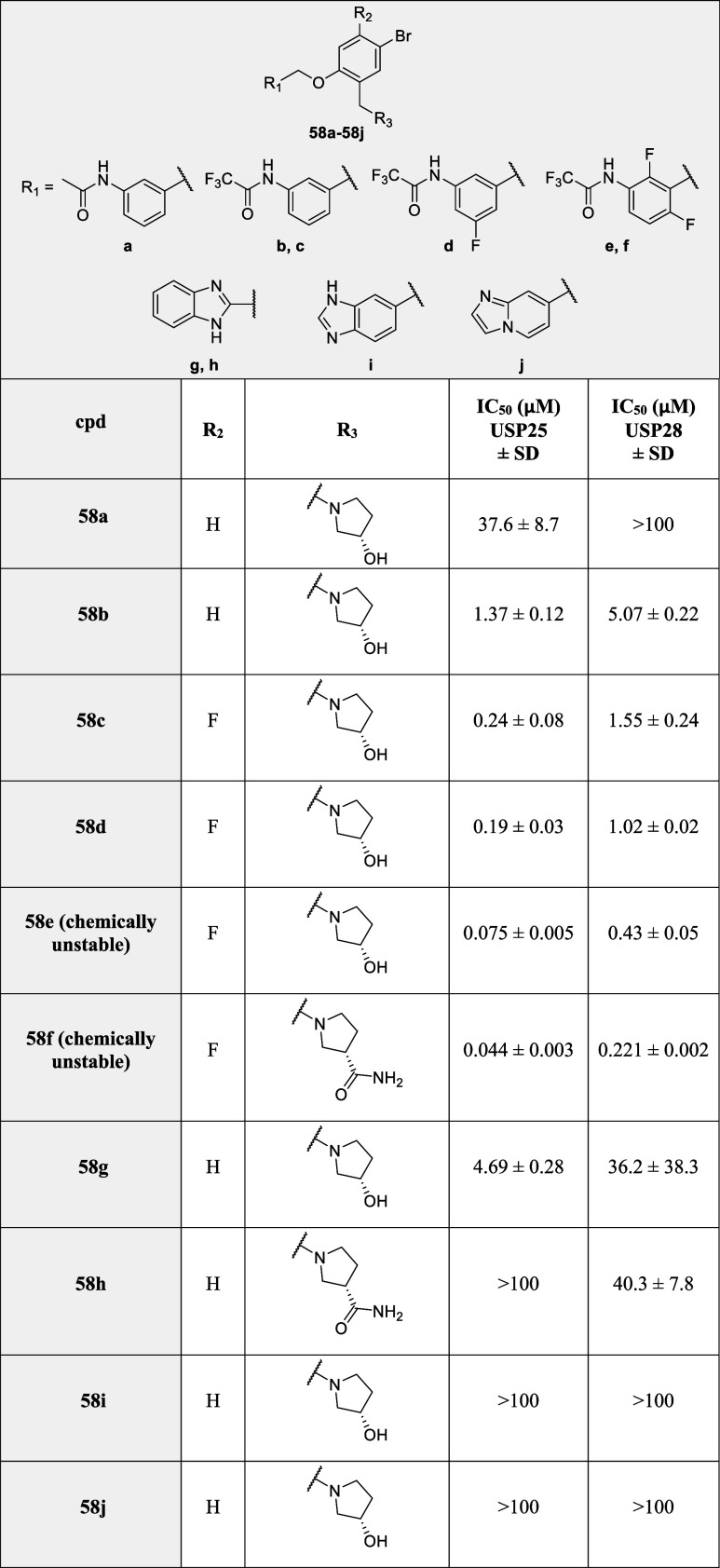
IC_50_ Results for Compounds **58a**–**58j**

**Figure 3:**
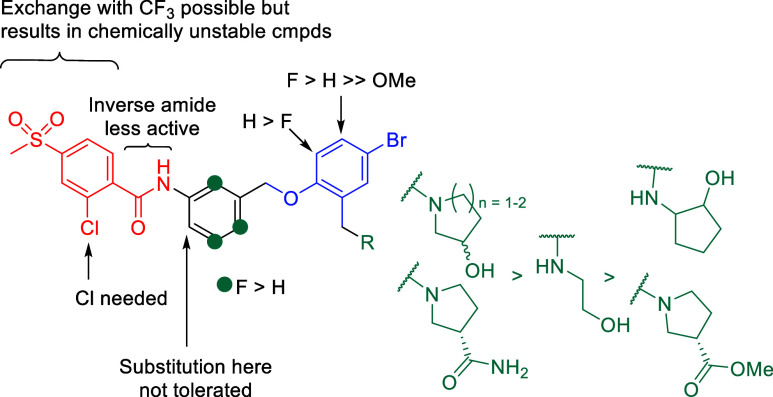
SAR summary.

### Verification of Binding Using Orthogonal Assays

Reference compounds AZ1, VSM, and the recently described USP25/USP28 inhibitor FT206 ^35,36^ together with the inactive compound **33** (**T-10507**) and potent compounds **36** and **42** (**T-10531**) were analyzed in differential scanning fluorimetry (DSF) experiments with USP25 and USP28. For USP25, only compounds FT206, **36**, and **T-10531** displayed Δ*T*_m_ shifts larger than 1 °C. The highest Δ*T*_m_ value of 7 °C was achieved by **T-10531** (Table 4 and Figure 4A). With respect to USP28, the same compounds led to a Δ*T*_m_ shift larger than 1 °C, whereby the largest shift was obtained for FT206 with 3.4 °C and **T-10531** with 3.8 °C.

**Table 4:** *T*_m_-Shift as Determined in DSF Assays for Each Protein and Compound^a^

cpd	USP25	USP28
**AZ1**	0.5	0.5
**VSM**	0.6	0.2
**FT206**	1.6	3.4
**33 (T-10507)**	–0.9	–2.1
**36**	3.4	2.3
**42 (T-10531)**	7.0	3.8

a Data presented is the median of *n* = 3.

**Figure 4:**
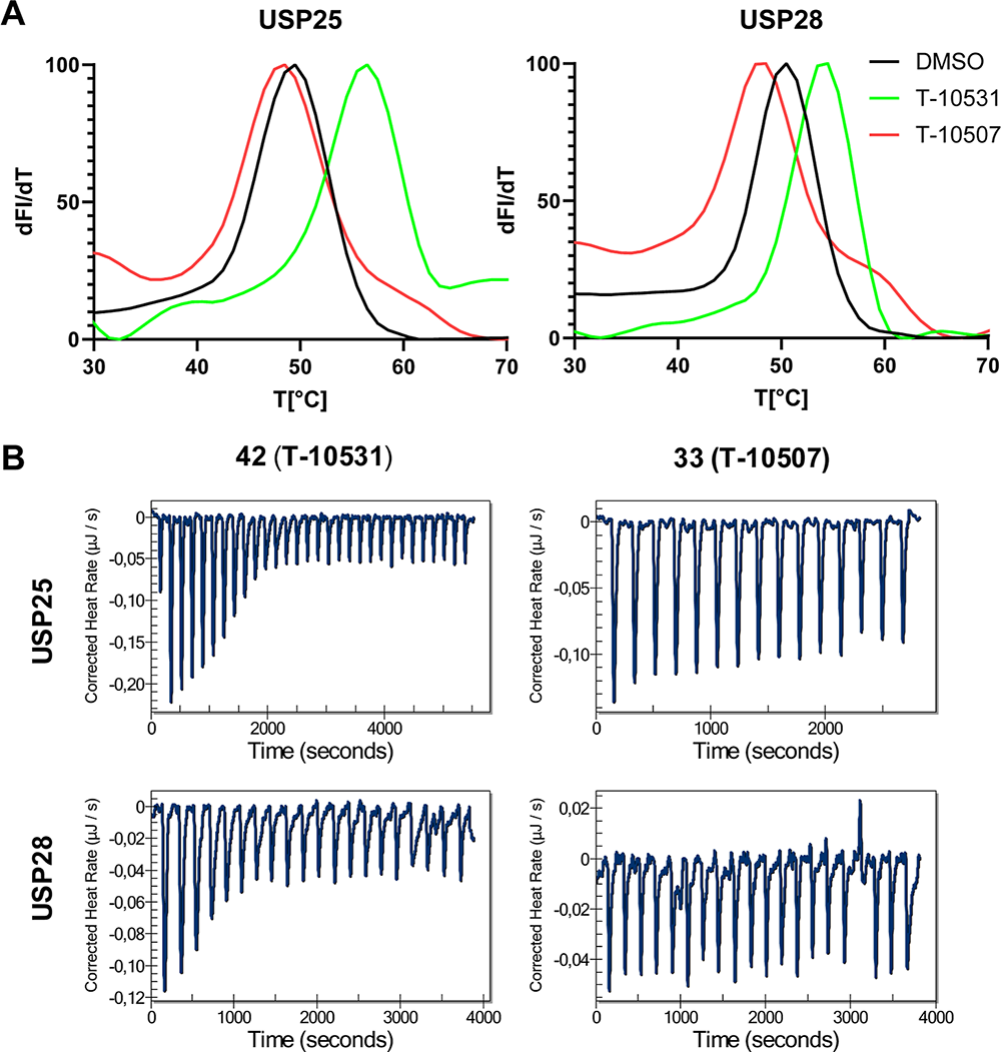
DSF and isothermal titration calorimetry (ITC) experiments. (A) Thermal shift graphs for compounds **T-10507** and **T-10531.** (B) ITC results for **T-10507** and **T-10531** with USP25 and USP28.

Subsequently, isothermal titration calorimetry (ITC) was performed to elucidate the binding affinities of **33** (**T-10507**) and **42** (**T-10531**) toward USP25 and USP28. Inverse titration was applied, and the protein solution was titrated to the compound solution in the cell (Figure 4B). For compound **42** (**T-10531**), a *K*_d_ of 0.2 ± 0.1 μM for USP25 and a *K*_d_ of 0.06 ± 0.2 μM for USP28 were determined. For compound **33** (**T-10507**), no significant binding was detected.

### Crystal Structure of USP28 in Complex with Inhibitor **T-10531**

A crystal structure of USP28 in complex with inhibitor **T-10531** was obtained at a resolution of 2.75 Å (Figure 5A). The USP28ΔUCID construct was used, as previously described.^33,34,37^ Following molecular replacement, a clear inhibitor density was obtained in the hydrophobic thumb-palm cleft of USP28, allowing for unambiguous placement of **T-10531** in the model (Figure S3). The observed inhibitor position is in the same cleft as AZ1 and VSM, providing experimental confirmation that the merged inhibitor targets USP28 at the targeted position (Figure S4). H261 and E366 form a hydrogen bond bridging over the thumb-palm cleft, enclosing **T-10531**, while simultaneously, E366 forms a hydrogen bond with the amide nitrogen of **T-10531**, as observed previously in the interaction between USP28 and VSM (Figure 1A).^33^ The methyl-sulfone moiety of **T-10531** is displaced by 1.2 Å away from the end of the cleft compared to the equivalent portion of VSM. As the *N*-terminal end of α5 was not resolved in the USP28:VSM structure, a direct comparison of the role of S257 in inhibitor binding is not possible. However, in the merged inhibitor **T-10531**, the position and interactions of the VSM-derived portion remain broadly similar. The pyrrolidine ring of **T-10531** emerges at the surface of the hydrophobic cleft in the space between D265 and Q315, supporting the hydroxyl group as it turns back to form a hydrogen bond with Q315. Interestingly, SAR suggests that the stereochemistry of the hydroxyl group is of minor importance—compound **23** ((*S*)-enantiomer) has only slightly higher inhibitory potency compared to compound **24** ((*R*)-enantiomer). One could speculate that the hydroxyl group of the (*R*)-enantiomer is engaged in hydrogen bonding toward D256.^35^

**Figure 5:**
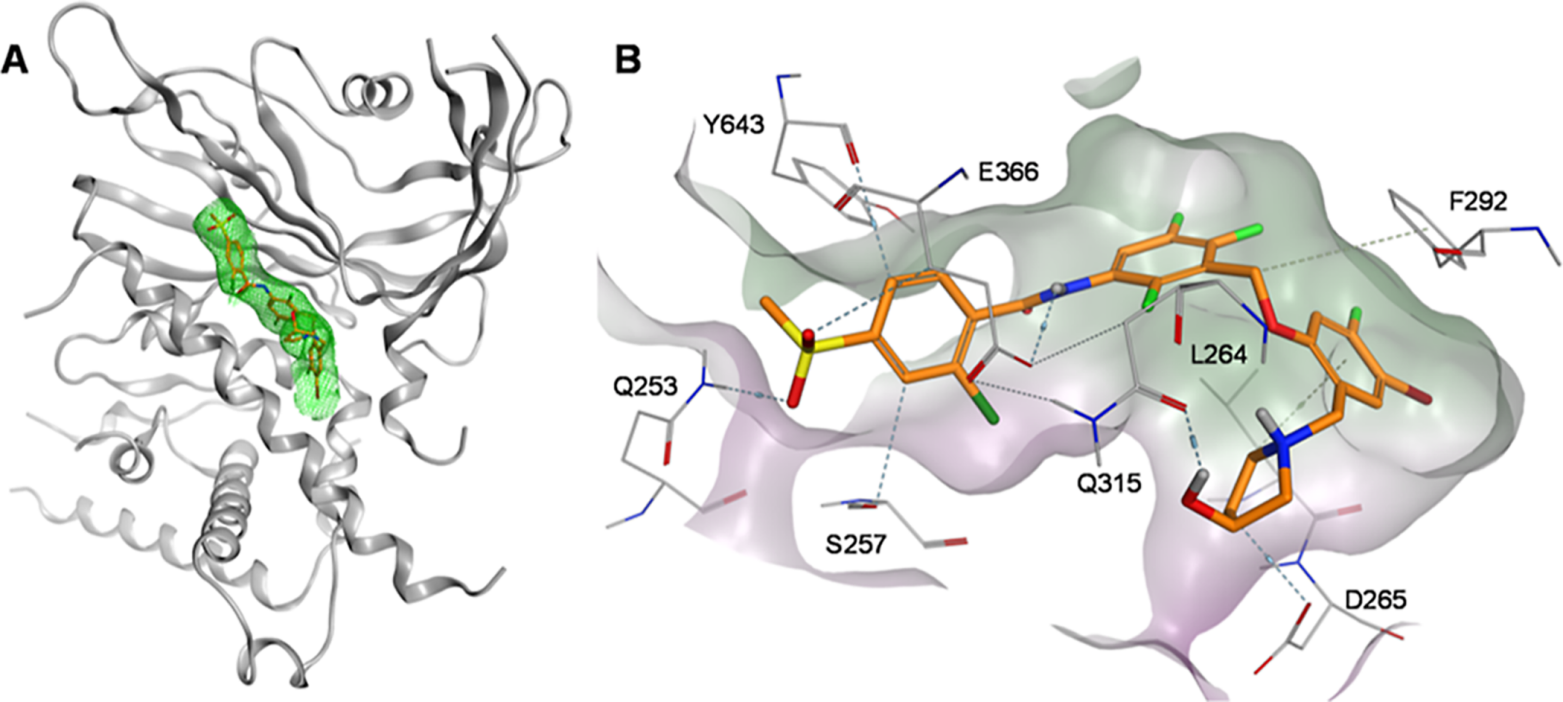
Crystal structure of USP28:T-10531 complex (PDB ID: 9SUU). (A) Crystal structure of the USP28: **T-10531** complex (USP28—gray ribbon, **T-10531**—orange sticks; green mesh represents the 2F_o_-F_c_ map around **T-10531** at 2σ). (B) Detail view of **T-10531** (orange) binding to USP28 (key side chains shown as lines). Hydrogen bonds are depicted as dashed lines.

As previously observed in the interaction between USP28 and AZ1, the bromophenol ring of **T-10531** engages with the side chain of F292 of USP28 via π-stacking. The Br-substituent fits nicely into the hydrophobic binding pocket. While a fluorine adjacent to bromine also fits the binding site, the introduction of the larger methoxy group leads to a loss in affinity. The positions of the fluorobenzene rings of **T-10531** and AZ1, and the chlorobenzene ring of VSM correspond well, demonstrating the success of merging AZ1 and VSM at this position. The amide NH forms a directed H-bond toward E366, and the amide is almost coplanar to the central phenyl ring. This explains the loss in affinity of compound **18**, which exhibits a fluorine in the 6-position and probably disturbs this geometry by repulsive interactions with the amide oxygen. The position of helix α5 in the **T-10531** bound state is most like the position of α5 in the VSM bound state; the methyl-sulfone group wedges the palm-thumb cleft in an open, apo-like position (Figure S4).

### Cell Toxicity, Aqueous Solubility, and Metabolic Stability

The toxicological profile of compound **42** (**T-10531**) was tested in hepatocellular carcinoma cells (HepG2; DSMZ #ACC 180) using CellTiter-Glo as a global measure for metabolic activity and cell survival, which were both unaffected (threshold ≥90%) after 72 h of treatment with the compound at up to 10 μM.

To evaluate the potential of **T-10531** as a chemical tool for *in vivo* studies, we first evaluated aqueous solubility and metabolic stability as key factors for good absorption and low clearance of the drug. Solubility was determined in PBS buffer, and *in vitro* metabolic half-life was studied using rat liver microsomes (Table 5). **T-10531** showed very high metabolic stability and good aqueous solubility of around 75 μM.

**Table 5:** Aqueous Solubility and Metabolic Stability Compound **T-10531**

cpd	aq. solubility^a^ ^,^ (μM)	rat liver microsomes^b^ (remaining after 60 min)	CL_int_ (μL min^–1^ mg^–1^)
**T-10531**	75 μM	86%^c^	4.15^d^

a Solubility of compound in PBS buffer, pH 7.4, containing 1% DMSO.

b Microsome mix from the liver of Sprague–Dawley rats.

c Percentage of remaining compound after 60 min.

d Intrinsic clearance calculated according to formula [Disp-formula eq1], see experimental part.

### The Developed Inhibitors Have Excellent Cellular Activity

For the determination of cellular target engagement, full-length USP25 and USP28 were cloned as NanoLuc luciferase fusions and expressed in HEK293T cells. As a tracer for this displacement assay, the dye compound (**61**) was titrated into the NanoLuc USP25/28 expressing cells for tracer *K*_d_ determination. Due to incomplete sigmoidal fits, the working concentration of the tracer was set to yield a sufficient signal compared to background values, which was obtained at a concentration of 1 μM for USP25 and 4 μM for USP28. No notable differences were observed comparing titration experiments in intact live cells and permeabilized digitonin-treated cells, suggesting good cell permeability of **61**. Subsequent compound titrations were carried out for the most interesting synthesized compounds. IC_50_ values for AZ1 and VSM were in the μM range, whereas the most active compounds of our merged inhibitor series reached sub-100 nM potencies (**40** and **42**). Compound **33**, a structurally similar analogue to **T-10531**, was found to be inactive in this assay, rendering it a suitable negative control (Figure 6 and Table 6).

**Figure 6:**
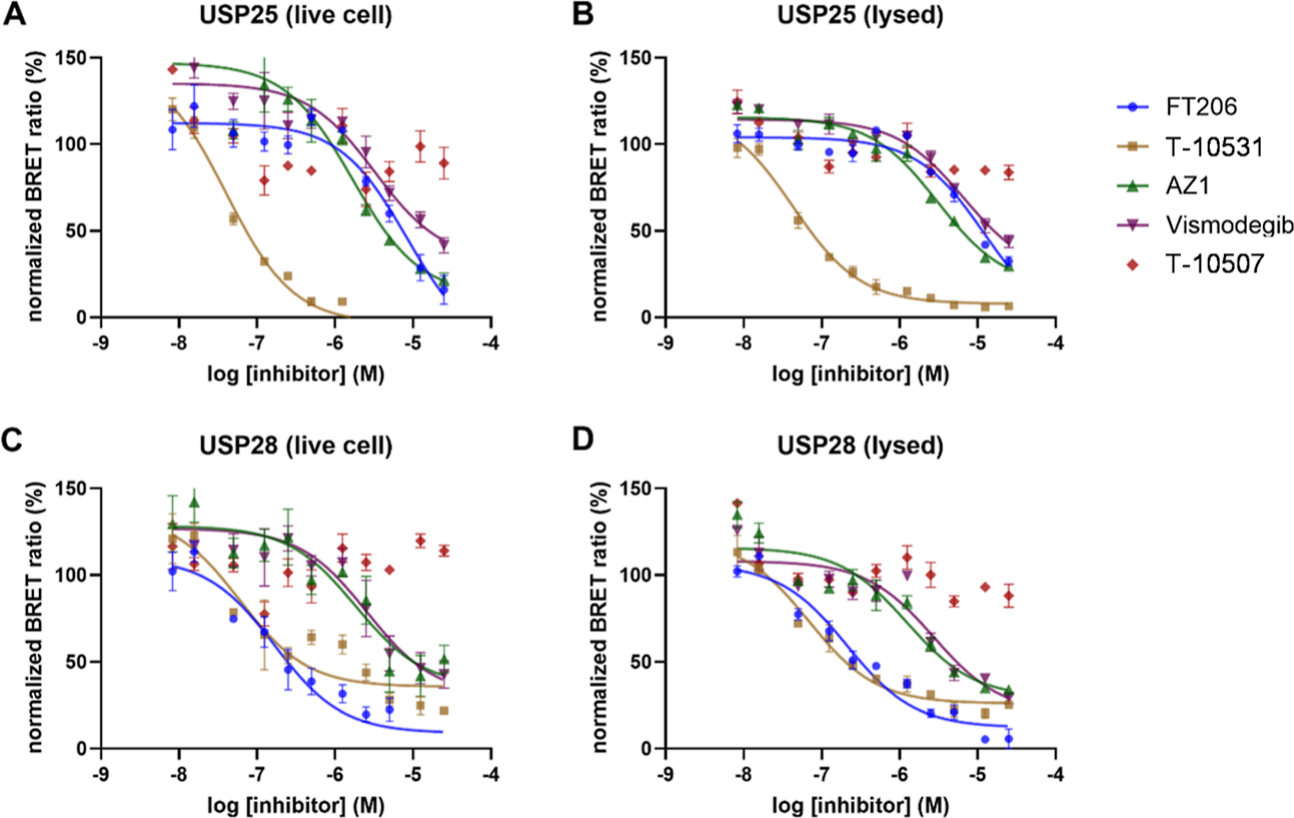
Cellular target engagement assay using developed bioluminescence resonance energy transfer (BRET) assays. Shown are dose-response data of the interaction of **T-10531** with USP25 (A) and USP28 (C) in live cells and lysed cells (permeabilized using digitonin) (B,D). The normalized BRET ratio is shown together with SE (*n* = 3).

**Table 6:** IC_50_ Values of Most Active Compounds and Literature Compounds Using the Developed BRET Assays in Live and in Lysed (Permeabilized) Cells

inhibitor	pIC_50_			
	USP25		USP28	
	live cell	lysed	live cell	lysed
FT206	5.07 ± 0.16	4.92 ± 0.14	6.77 ± 0.14	6.67 ± 0.09
**35**	6.20 ± 0.14	6.10 ± 0.09	5.65 ± 0.13	5.74 ± 0.08
**36**	7.02 ± 0.16	6.84 ± 0.06	6.54 ± 0.20	6.44 ± 0.11
**38**	6.58 ± 0.10	6.62 ± 0.06	6.06 ± 0.28	6.36 ± 0.13
**40**	7.33 ± 0.10	7.05 ± 0.07	6.89 ± 0.20	6.80 ± 0.08
**41**	7.65 ± 0.10	7.39 ± 0.06	7.12 ± 0.14	7.05 ± 0.08
**42**	7.40 ± 0.07	7.37 ± 0.07	7.22 ± 0.20	7.16 ± 0.10
AZ1	5.78 ± 0.14	5.51 ± 0.08	5.73 ± 0.22	5.91 ± 0.14
Vismodegib	5.49 ± 0.16	5.18 ± 0.19	5.59 ± 0.21	5.56 ± 0.16

To investigate whether **T-10531** inhibits USP25/28 in live cells, HeLa cells were treated with the inhibitor at concentrations ranging from 0.1 to 50 μM for 4 h. Following treatment, the cells were lysed, and the lysates were incubated with a fluorescent broad-spectrum DUB probe, rhodamine-biotin-ubiquitin-propargylamide (Rho-K­(Biotin)-Ub-PA), for 10 min at 37 °C to label cysteine-DUB activity.^37,38^ The samples were denatured, resolved by SDS-PAGE, and scanned for rhodamine fluorescence (Figure 7A). Each band corresponds to an active DUB that reacted with the probe, while the disappearance of a band indicates that the compound effectively inhibited the corresponding DUB. Here, we demonstrated that **T-10531** does not affect the activity of highly abundant or active DUBs. To validate the inhibition of USP25 and USP28 by **T-10531**, we performed immunoblotting for both DUBs. This analysis confirmed that the DUB-ABP bands corresponding to USP25 and USP28 disappeared upon treatment with **T-10531** in a dose-dependent manner. Furthermore, the immunoblot revealed a dose-dependent decrease in the protein levels of USP25, but not of USP28. This reduction in USP25 levels, following treatment with **T-10531**, caught our attention for further investigation.

**Figure 7:**
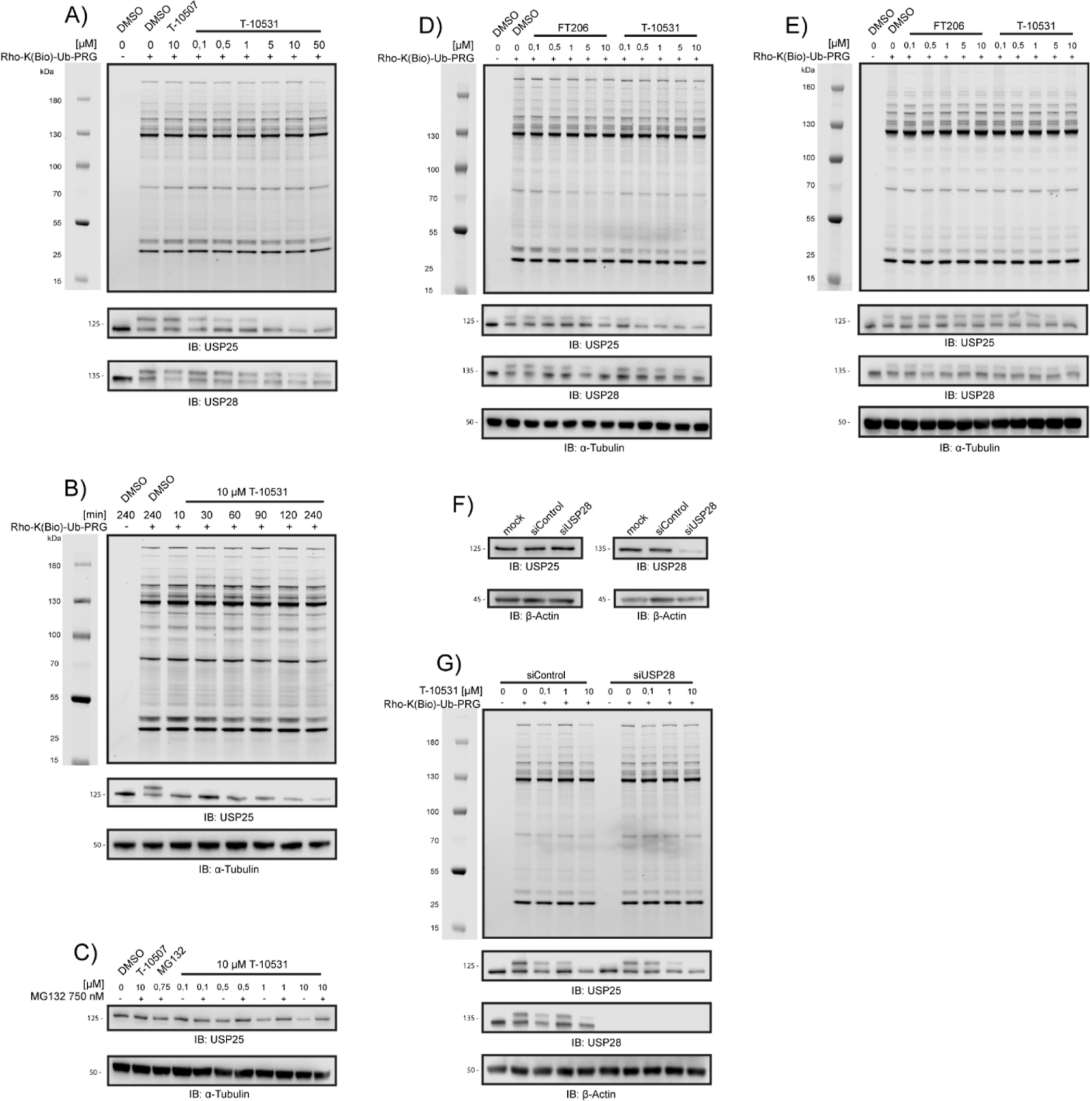
Investigation of selectivity within the cysteine-DUB family using activity-based protein profiling. (A) Incubation of living HeLa cells for 4 h with **T-10531** at concentrations ranging from 0.1 to 50 μM, followed by lysate labeling with ABP for 10 min at 37 °C does not show inhibition of the DUB panel. (B) Incubation of HeLa cells with 10 μM **T-10531** for 10–240 min shows time dependence of USP25 inhibition. (C) Proteasome inhibition with MG132 and incubation with **T-10531** for 4 h in living HeLa cells, shows that proteasome inhibition rescues USP25 degradation as observed with **T-10531**. (D) Incubation of living HeLa cells for 4 h with **T-10531** and FT206 at concentrations ranging from 0.1 to 10 μM, followed by lysate labeling with ABP for 10 min at 37 °C does not show inhibition of the DUB panel. Incubation with **T-10531** leads to degradation of USP25. (E) Incubation of HeLa cell lysates for 5 min with **T-10531** and FT206 at concentrations ranging from 0.1 to 10 μM, followed by lysate labeling with ABP for 10 min at 37 °C does not show inhibition of the DUB panel. (F) Silencing of USP28 shows no influence on USP25 protein level. (G) Silencing of USP28 and subsequential treatment with **T-10531** for 4 h at concentrations ranging from 0.1 to 10 μM shows USP25 degradation independent of USP28.

First, we determined whether the decrease in USP25 levels occurs after shorter time periods and whether its potency increases over longer durations. A time-dependent experiment was conducted using 10 μM **T-10531**, with incubation times ranging from 10 to 240 min. USP25 levels decreased by approximately 50% after 10 min, with a further reduction to 80% after 240 min (Figure 7B). To investigate whether the reduction in USP25 levels is proteasome-dependent, we tested whether proteasome inhibition with MG132 could mitigate this effect. Our data revealed that USP25 degradation indeed occurs via the proteasome (Figure 7C). We also considered the possibility that the reduction in USP25 levels might result from USP28 inhibition, as USP28 could potentially act as a deubiquitinating enzyme for USP25. Additionally, it remained unclear whether the decreased USP25 levels were due to the structural properties of **T-10531** or its inhibition of USP25, leading to autodeubiquitination.

To address these questions, we tested a structurally unrelated dual USP25/USP28 inhibitor, FT206, which exhibits a higher preference for USP28 inhibition, whereas **T-10531** shows a preference for USP25. Live HeLa cells were incubated with both inhibitors, followed by probe labeling of the lysates. Immunoblotting confirmed that FT206 preferentially inhibits USP28, while **T-10531** shows a preference for USP25. Notably, in experiments conducted on cells, treatment with FT206 did not result in a decrease in USP25 levels, whereas **T-10531** consistently reduced USP25 levels (Figure 7D). In contrast, when lysates were incubated with FT206 and **T-10531**, both compounds retained their target preferences; however, no decrease in DUB protein levels was observed under these conditions (Figure 7E). These results suggest that the decrease in USP25 levels is not due to USP28 inhibition. For further analysis, we performed a knockdown of USP28, which had no impact on USP25 levels (Figure 7F). Finally, silencing USP28 followed by incubation with **T-10531** for 4 h and subsequent probe labeling confirmed that USP28 does not influence USP25 degradation (Figure 7G). Together, these findings indicate that USP28 is not responsible for the deubiquitination of USP25, and USP25 is likely undergoing autodeubiquitination. This autodeubiquitination is reduced by USP25 inhibition, leading to degradation via the proteasome.

## Conclusions

The enzymes USP25 and USP28 have emerged as promising therapeutic targets in recent years. In order to validate their role in physiological and pathophysiological processes, as well as to conduct basic investigations into their function, chemical probes are required. In this study, we designed new USP25/28 inhibitors using a structure merging approach from known inhibitors AZ1 and vismodegib. As a result, here we report new highly potent inhibitors. Compound **42** (**T-10531**), in particular, exhibited high potency in orthogonal assays, selectivity over other USPs and showed low toxicity. Our analysis shows that **T-10531** is qualified for use as a chemical probe to study the consequences of USP25/USP28 inhibition *in vitro*, while related compound **T-10507** was inactive and validated as a negative control.

## Experimental Section

### General

Chemicals for the synthesis of **1**–**62** were purchased from Acros Organics (Geel, Belgium), Alfa Aesar GmbH & Co KG (Karlsruhe, Germany), BLD Pharmatech GmbH (Kaiserslautern, Germany), Enamine Ltd. (Kyiv, Ukraine), Sigma-Aldrich Chemie GmbH (Steinheim, Germany), and TCI Deutschland (Eschborn, Germany).

Reactions were monitored via thin-layer chromatography using ALUGRAM from Merck (Darmstadt, Germany). To record NMR spectra, compounds were dissolved in DMSO-*d*_6_, CDCl_3_, or CD_2_Cl_2_ and measured on DPX250, Avance 300, Avance 400, and Avance 500 from Bruker Corporation (Massachusetts, USA) using tetramethylsilane as an internal standard. All chemical shift values are reported in ppm, the multiplicity of the signals was assigned as follows: s (singlet), d (duplet), t (triplet), and m (multiplet). Mass spectrometry analysis was performed in positive ion mode by electrospray-ionization (ESI) on a LCMS-2020 single quadrupole MS from Shimadzu (Duisburg, Deutschland). Precision mass was measured using the MALDI Orbitrap XL from Life Technologies GmbH (Darmstadt, Germany). For purity estimation of the synthesized compounds, a reverse-phase high-performance liquid chromatography was performed using the Luna 10 μm C18(2) 100 Å, LC Column 250 × 4.6 mm from Phenomenex LTD (Aschaffenburg, Germany), and the analysis was conducted using the Shimadzu Prominence module from Shimadzu. Acetonitrile and aqueous formic acid 0.1% were used as eluents. The established method for purity determination was initiated with 90% water (0.1% formic acid), then a linear gradient from 90% to 5% water (0.1% formic acid) for 13 min was chosen, and finally an additional 7 min 5% water (0.1% formic acid). The flow rate was adjusted to 1.0 mL/min, and the UV–vis detection occurred at 254 and 280 nm, respectively. Purity of all tested compounds was determined to be higher than 95%.

### Synthetic Methods

Altogether, 47 new final products were synthesized, analyzed, and tested. Pure compounds (≥95% purity) were obtained after purification by reverse-phase HPLC (see Experimental Section). The structures of all final products were confirmed by ^1^H, ^13^C NMR, and ^19^F (if applicable) spectroscopy and HPLC analysis coupled to electrospray ionization mass spectrometry (HPLC/ESI-MS), which was also used to determine the purity. Additionally, HRMS was also determined.

### General Procedures

#### GP1. Reduction of Aryl Carboxyl Groups

An ice-cooled suspension of sodium borohydride (2 equiv) in THF is treated with a solution of a benzoic acid derivative (1 equiv) in THF. Boron trifluoride etherate (2.7 equiv) is then added slowly. The solution is stirred at room temperature overnight and quenched with an aqueous solution of HCl (1 M). Afterward, the product is extracted with EtOAc three times. The combined organic layers are washed with water two times and brine one time, dried over magnesium sulfate, and evaporated under reduced pressure to yield the crude product. If necessary, the crude product is purified by flash chromatography.

#### GP2. Reduction of Aromatic Nitro Groups

A solution of tin­(II) chloride dihydrate (4 equiv) in concentrated HCl (5 mL) is added to a solution of an aromatic nitro derivative (1 equiv) in 5 mL of ethanol and 5 mL of concentrated HCl. The resulting solution is stirred at room temperature overnight. A saturated, aqueous solution of NaHCO_3_ is added, and the product is extracted with EtOAc three times. The combined organic layers are washed with brine, dried over magnesium sulfate, and evaporated under reduced pressure. If necessary, the crude product is purified by flash chromatography.

#### GP3. Amide Bond Formation

An aromatic acyl chloride (1 equiv) is dissolved in DCM and cooled to 0 °C. A solution of an aniline derivative (2 equiv) in DCM is added dropwise at 0 °C. The resulting solution is stirred at room temperature for 2 h. Then, the reaction mixture is diluted with aqueous solution of HCl (1 M), and the product is extracted with EtOAc three times. The combined organic layers are dried over magnesium sulfate, filtered, and evaporated. If necessary, the crude product is purified by flash chromatography.

#### GP4. Chlorination of Benzylic Alcohols

A solution of an alcohol (1 equiv) in DCE is treated with thionyl chloride (30 to 60 equiv), and the resulting suspension is stirred at 55 °C overnight. The reaction mixture is diluted with DCM and washed with water. The organic layer is dried over magnesium sulfate, filtered, and evaporated to yield the crude product, which was used in the next step without further purification or purified by flash chromatography.

#### GP5. Williamson Ether Synthesis

An alkyl chloride (1 equiv), a phenol (1.2 equiv), and potassium carbonate (2 equiv) are dissolved in DMF, and the resulting solution is stirred at 60 °C overnight. After this time, the reaction mixture is evaporated under reduced pressure and diluted with water. The product is extracted with EtOAc three times, and the combined organic layers are dried over magnesium sulfate, filtered, and evaporated under reduced pressure. The crude product is purified by flash chromatography.

#### GP6. Reductive Amination

An amine (4 equiv) and acetic acid (6 equiv) are added to a solution of an aryl aldehyde (1 equiv) in DMF. The reaction mixture is stirred for 20 min before sodium cyanoborohydride or sodium triacetoxyborohydride (4 equiv) is added. The solution is stirred at room temperature overnight. Then, a saturated, aqueous solution of NaHCO_3_ is added, and the aqueous phase is extracted with EtOAc three times. The combined organic layers are dried over magnesium sulfate, filtered, and evaporated under reduced pressure. The crude product is purified by preparative HPLC.

#### Synthesis of Final Compounds **14**–**45** and **50**

##### 2-Chloro-*N*-(3-(hydroxymethyl)­phenyl)-4-(methylsulfonyl)­benzamide (**5a**)

The synthesis was performed according to GP3 using 2-chloro-4-(methylsulfonyl)­benzoyl chloride (253 mg, 1.0 mmol, 1.0 equiv) and 3-aminobenzyl alcohol (246 mg, 2.0 mmol, 2.0 equiv) in DCM (10 mL). The product **5a** was obtained in a yield of 95% (322 mg). ^1^H NMR (250 MHz, DMSO-*d*_6_): δ 10.65 (s, 1H), 8.12 (d, *J* = 1.5 Hz, 1H), 8.00 (dd, *J* = 8.0, 1.7 Hz, 1H), 7.86 (d, *J* = 8.0 Hz, 1H), 7.71 (t, *J* = 1.5 Hz, 1H), 7.57–7.51 (m, 1H), 7.31 (t, *J* = 7.7 Hz, 1H), 7.08 (d, *J* = 7.9 Hz, 1H), 5.22 (t, *J* = 5.7 Hz, 1H), 4.50 (d, *J* = 5.7 Hz, 2H), 3.34 (s, 3H).

##### 2-Chloro-*N*-(3-(hydroxymethyl)­benzyl)-4-(methylsulfonyl)­benzamide (**6**)

The synthesis was performed according to GP3 using 2-chloro-4-(methylsulfonyl)­benzoyl chloride (380 mg, 1.5 mmol, 1.0 equiv) and 3-(aminomethyl)­benzyl alcohol (412 mg, 3.0 mmol, 2.0 equiv) in DCM (12 mL). The product **6** was obtained in a yield of 97% (515 mg). ^1^H NMR (250 MHz, DMSO-*d*_6_): δ 9.15 (t, *J* = 5.8 Hz, 1H), 8.05 (d, *J* = 1.7 Hz, 1H), 7.94 (dd, *J* = 8.0, 1.7 Hz, 1H), 7.72 (d, *J* = 8.0 Hz, 1H), 7.34–7.27 (m, 2H), 7.25–7.19 (m, 2H), 5.18 (t, *J* = 5.7 Hz, 1H), 4.49 (d, *J* = 5.7 Hz, 2H), 3.32 (s, 3H).

##### 2-Chloro-*N*-(3-(hydroxymethyl)­phenethyl)-4-(methylsulfonyl)­benzamide (**7**)

2-Chloro-4-(methylsulfonyl)­benzoyl chloride (380 mg, 1.5 mmol, 1.0 equiv) was dissolved in DCM (5 mL) and cooled to 0 °C. 3-(2-Aminoethyl)­phenyl]­methanol hydrochloride (282 mg, 1.5 mmol, 1.0 equiv) and trimethylamine (422 μL, 3.0 mmol, 2.0 equiv) in DCM (3 mL) were added dropwise at 0 °C. The resulting solution was stirred at rt for 2 h. The reaction mixture was diluted with aqueous HCl 1 M and extracted with EtOAc (3×). The combined organic layers were dried over magnesium sulfate, filtered, and evaporated. The residue was purified by flash chromatography (hexane/EtOAc 1:1 to 0:1) to yield 288 mg (52%) of **7**. ^1^H NMR (250 MHz, DMSO-*d*_6_): δ 8.77–8.60 (m, 1H), 8.10–7.79 (m, 1H), 7.66–7.50 (m, 1H), 7.31–7.01 (m, 4H), 5.15 (m, 1H), 4.49 (m, 2H), 3.46 (m, 2H), 3.32 (s, 3H), 2.82 (m, 2H).

##### 2-Chloro-*N*-(2-fluoro-3-(hydroxymethyl)­phenyl)-4-(methylsulfonyl)­benzamide (**5b**)

The synthesis was performed according to GP3 using 2-chloro-4-(methylsulfonyl)­benzoyl chloride (253 mg, 1.0 mmol, 1.0 equiv) and 3-amino-2-fluorobenzyl alcohol (282 mg, 2.0 mmol, 2.0 equiv) in DCM (10 mL). The residue was purified by flash chromatography (hexane/EtOAc 1:1 to 0:1) to yield 347 mg (97%) of **5b**. ^1^H NMR (250 MHz, DMSO-*d*_6_): δ 10.51 (s, 1H), 8.11 (d, *J* = 1.5 Hz, 1H), 7.99 (dd, *J* = 8.0, 1.6 Hz, 1H), 7.86 (d, *J* = 8.0 Hz, 1H), 7.75 (td, *J* = 7.6, 1.4 Hz, 1H), 7.32 (td, *J* = 6.3, 1.4 Hz, 1H), 7.21 (t, *J* = 7.9 Hz, 1H), 5.32 (t, *J* = 5.7 Hz, 1H), 4.57 (d, *J* = 5.7 Hz, 2H), 3.34 (s, 3H).

##### 2-Chloro-*N*-(2-fluoro-5-(hydroxymethyl)­phenyl)-4-(methylsulfonyl)­benzamide (**5c**)

The synthesis was performed according to GP3 using 2-chloro-4-(methylsulfonyl)­benzoyl chloride (253 mg, 1.0 mmol, 1.0 equiv) and (3-amino-4-fluorophenyl)­methanol (282 mg, 2.0 mmol, 2.0 equiv) in DCM (10 mL). The residue was purified by flash chromatography (hexane/EtOAc 1:1 to 0:1) to yield 307 mg (86%) of **5c**. ^1^H NMR (250 MHz, DMSO-*d*_6_): δ 10.52 (s, 1H), 8.11 (d, *J* = 1.6 Hz, 1H), 7.99 (dd, *J* = 8.0, 1.6 Hz, 1H), 7.86 (d, *J* = 8.0 Hz, 1H), 7.84 (dd, *J* = 7.6, 1.8 Hz, 1H), 7.29–7.14 (m, 2H), 5.30 (t, *J* = 5.7 Hz, 1H), 4.49 (d, *J* = 5.7 Hz, 2H), 3.34 (s, 3H).

##### 2-Chloro-*N*-(3-fluoro-5-(hydroxymethyl)­phenyl)-4-(methylsulfonyl)­benzamide (**5d**)

The synthesis was performed according to GP3 using 2-chloro-4-(methylsulfonyl)­benzoyl chloride (253 mg, 1.0 mmol, 1.0 equiv) and 3-amino-2-fluorobenzyl alcohol (282 mg, 2.0 mmol, 2.0 equiv) in DCM (10 mL). The residue was purified by flash chromatography (hexane/EtOAc 1:1 to 0:1) to yield 320 mg (90%) of **5d**. ^1^H NMR (250 MHz, DMSO-*d*_6_): δ 10.86 (s, 1H), 8.12 (d, *J* = 1.6 Hz, 1H), 8.00 (dd, *J* = 8.0, 1.6 Hz, 1H), 7.88 (d, *J* = 8.0 Hz, 1H), 7.75 (dt, *J* = 10.9, 2.3 Hz, 1H), 7.44 (s, 1H), 6.93–6.87 (m, 1H), 5.37 (t, *J* = 5.7 Hz, 1H), 4.51 (d, *J* = 5.7 Hz, 2H), 3.34 (s, 3H).

##### 2-Chloro-*N*-(4-fluoro-3-(hydroxymethyl)­phenyl)-4-(methylsulfonyl)­benzamide (**5e**)

The synthesis was performed according to GP3 using 2-chloro-4-(methylsulfonyl)­benzoyl chloride (253 mg, 1.0 mmol, 1.0 equiv) and (5-amino-2-fluorophenyl)­methanol (282 mg, 2.0 mmol, 2.0 equiv) in DCM (10 mL). The residue was purified by flash chromatography (hexane/EtOAc 1:1 to 0:1) to yield 322 mg (90%) of **5e**. ^1^H NMR (250 MHz, DMSO-*d*_6_): δ 10.71 (s, 1H), 8.12 (d, *J* = 1.6 Hz, 1H), 8.00 (dd, *J* = 8.0, 1.6 Hz, 1H), 7.87 (d, *J* = 8.0 Hz, 1H), 7.84 (dd, *J* = 6.5, 2.7 Hz, 1H), 7.63–7.56 (m, 1H), 7.15 (t, *J* = 9.7 Hz, 1H), 5.34 (t, *J* = 5.7 Hz, 1H), 4.55 (d, *J* = 5.7 Hz, 2H), 3.34 (s, 3H).

##### 2-Chloro-*N*-(4-chloro-3-(hydroxymethyl)­phenyl)-4-(methylsulfonyl)­benzamide (**5f**)

The synthesis was performed according to GP3 using 2-chloro-4-(methylsulfonyl)­benzoyl chloride (253 mg, 1.0 mmol, 1.0 equiv) and 3-amino-2-chlorobenzyl alcohol (315 mg, 2.0 mmol, 2.0 equiv) in DCM (10 mL). The residue was purified by flash chromatography (hexane/EtOAc 6:4 to 3:7) to yield 333 mg (89%) of **5f**. ^1^H NMR (250 MHz, DMSO-*d*_6_): δ 10.81 (s, 1H), 8.12 (d, *J* = 1.6 Hz, 1H), 8.00 (dd, *J* = 8.0, 1.6 Hz, 1H), 7.93 (d, *J* = 2.5 Hz, 1H), 7.88 (d, *J* = 8.0 Hz, 1H), 7.64 (dd, *J* = 8.7, 2.6 Hz, 1H), 7.39 (d, *J* = 8.6 Hz, 1H), 5.47 (t, *J* = 5.7 Hz, 1H), 4.55 (d, *J* = 5.7 Hz, 2H), 3.34 (s, 3H).

##### *N*-(4-Bromo-3-(hydroxymethyl)­phenyl)-2-chloro-4-(methylsulfonyl)­benzamide (**5g**)

The synthesis was performed according to GP3 using 2-chloro-4-(methylsulfonyl)­benzoyl chloride (253 mg, 1.0 mmol, 1.0 equiv) and 5-amino-2-bromobenzenemethanol (404 mg, 2.0 mmol, 2.0 equiv) in DCM (10 mL). The residue was purified by flash chromatography (hexane/EtOAc 6:4 to 3:7) to yield 349 mg (83%) of **5g**. ^1^H NMR (250 MHz, DMSO-*d*_6_): δ 10.81 (s, 1H), 8.12 (d, *J* = 1.6 Hz, 1H), 8.00 (dd, *J* = 8.0, 1.6 Hz, 1H), 7.92 (d, *J* = 2.1 Hz, 1H), 7.88 (d, *J* = 8.0 Hz, 1H), 7.61–7.52 (m, 1H), 5.51 (t, *J* = 5.7 Hz, 1H), 4.50 (d, *J* = 5.7 Hz, 2H), 3.34 (s, 3H).

##### 2-Chloro-*N*-(2,4-difluoro-3-(hydroxymethyl)­phenyl)-4-(methylsulfonyl)­benzamide (**5h**)

The synthesis was performed according to GP3 using 2-chloro-4-(methylsulfonyl)­benzoyl chloride (239 mg, 0.945 mmol, 1.0 equiv) and **2h** (300 mg, 1.89 mmol, 2.0 equiv) in DCM (10 mL). The residue was purified by flash chromatography (hexane/EtOAc 1:1 to 0:1) to yield 355 mg (quant.) of **5h**. ^1^H NMR (250 MHz, DMSO-*d*_6_): δ 10.54 (s, 1H), 8.11 (d, *J* = 1.6 Hz, 1H), 8.00 (dt, *J* = 7.9, 1.5 Hz, 1H), 7.87 (d, *J* = 8.0 Hz, 1H), 7.76 (td, *J* = 8.9, 6.1 Hz, 1H), 7.13 (td, *J* = 8.9, 2.1 Hz, 1H), 5.32 (t, *J* = 5.7 Hz, 1H), 4.54 (d, *J* = 5.7 Hz, 2H), 3.34 (s, 3H).

##### 2-Chloro-*N*-(3,4-difluoro-5-(hydroxymethyl)­phenyl)-4-(methylsulfonyl)­benzamide (**5i**)

The synthesis was performed according to GP3 using 2-chloro-4-(methylsulfonyl)­benzoyl chloride (390 mg, 1.54 mmol, 1.0 equiv) and **2i** (490 mg, 3.08 mmol, 2.0 equiv) in DCM (10 mL). The residue was purified by flash chromatography (hexane/EtOAc 1:1 to 0:1) to yield 578 mg (99%) of **5i**. ^1^H NMR (250 MHz, DMSO-*d*_6_): δ 10.91 (s, 1H), 8.12 (d, *J* = 1.6 Hz, 1H), 7.99 (d, *J* = 7.9, 1.6 Hz, 1H), 7.88 (d, *J* = 7.9 Hz, 1H), 7.78–7.69 (m, 1H), 7.56–7.54 (m, 1H), 4.58 (s, 2H), 3.34 (s, 3H).

##### 2-Chloro-*N*-(2,5-difluoro-3-(hydroxymethyl)­phenyl)-4-(methylsulfonyl)­benzamide (**5j**)

The synthesis was performed according to GP3 using 2-chloro-4-(methylsulfonyl)­benzoyl chloride (215 mg, 0.850 mmol, 1.0 equiv) and **2j** (270 mg, 1.70 mmol, 2.0 equiv) in DCM (10 mL). The residue was purified by flash chromatography (hexane/EtOAc 1:1 to 0:1) to yield 297 mg (93%) of **5j**. ^1^H NMR (250 MHz, DMSO-*d*_6_): δ 10.73 (s, 1H), 8.11 (d, *J* = 1.6 Hz, 1H), 8.00 (dd, *J* = 8.0, 1.7 Hz, 1H), 7.87 (d, *J* = 8.0 Hz, 1H), 7.79–7.71 (m, 1H), 7.14–7.07 (m, 1H), 4.57 (s, 2H), 3.34 (s, 3H).

##### 2-Chloro-4-(methylsulfonyl)-*N*-(2,4,5-trifluoro-3-(hydroxymethyl)­phenyl)­benzamide (**5k**)

The synthesis was performed according to GP3 using 2-chloro-4-(methylsulfonyl)­benzoyl chloride (313 mg, 1.24 mmol, 1.0 equiv) and **2k** (437 mg, 2.47 mmol, 2.0 equiv) in DCM (14 mL). The product **5k** was obtained in a quantitative yield (488 mg) and used in the next step without further purification. ^1^H NMR (250 MHz, DMSO-*d*_6_): δ 10.75 (s, 1H), 8.12 (d, *J* = 1.6 Hz, 1H), 8.01 (dd, *J* = 7.9, 1.6 Hz, 1H), 7.87 (d, *J* = 7.9 Hz, 1H), 6.72–6.60 (m, 1H), 4.58 (s, 2H), 4.46 (s, 1H), 3.34 (s, 3H).

##### *N*-(4-Bromo-2-fluoro-3-(hydroxymethyl)­phenyl)-2-chloro-4-(methylsulfonyl)­benzamide (**5l**)

The synthesis was performed according to GP3 using 2-chloro-4-(methylsulfonyl)­benzoyl chloride (213 mg, 0.84 mmol, 1.0 equiv) and **2l** (370 mg, 1.68 mmol, 2.0 equiv) in DCM (11 mL). The residue was purified by flash chromatography (hexane/EtOAc 8:2 to 3:7) to yield 273 mg (74%) of **5l**. ^1^H NMR (400 MHz, DMSO-*d*_6_): δ 10.65 (s, 1H), 8.11 (s, 1H), 8.00 (d, *J* = 7.9 Hz, 1H), 7.87 (d, *J* = 7.9 Hz, 1H), 7.84 (t, *J* = 8.3 Hz, 1H), 7.52 (d, *J* = 8.7 Hz, 1H), 5.29 (t, *J* = 5.5 Hz, 1H), 4.62–4.59 (m, 2H), 3.34 (s, 3H).

##### *N*-(3-(Hydroxymethyl)­phenyl)-4-(methylsulfonyl)­benzamide (**5m**)

The synthesis was performed according to GP3 using 4-(methylsulfonyl)­benzoyl chloride (100 mg, 0.457 mmol, 1.0 equiv) and 3-aminobenzylalcohol (113 mg, 0.914 mmol, 2.0 equiv) in DCM (7.0 mL). The residue was purified by flash chromatography (hexane/EtOAc 1:1 to 0:1) to yield 49.3 mg (35%) of **5m**. ^1^H NMR (250 MHz, DMSO-*d*_6_): δ 10.65 (s, 1H), 8.12–7.96 (m, 4H), 7.86 (d, *J* = 8.0 Hz, 1H), 7.71 (s, 1H), 7.54 (d, *J* = 7.9 Hz, 1H), 7.31 (t, *J* = 7.9 Hz, 1H), 7.08 (d, *J* = 7.9 Hz, 1H), 5.22 (br s, 1H), 4.50 (s, 2H), 3.34 (s, 3H).

##### 2-Chloro-*N*-(4-(hydroxymethyl)­phenyl)-4-(methylsulfonyl)­benzamide­(**47**)

The synthesis was performed according to GP3 using 2-chloro-4-(methylsulfonyl)­benzoyl chloride (127 mg, 0.50 mmol, 1.0 equiv) and 4-aminobenzyl alcohol (124 mg, 1.0 mmol, 2.0 equiv) in DCM (8.0 mL). The residue was purified by flash chromatography (hexane/EtOAc 5:5 to 0:1) to yield 141 mg (83%) of **47**. ^1^H NMR (250 MHz, DMSO-*d*_6_): δ 10.64 (s, 1H), 8.12 (d, *J* = 1.7 Hz, 1H), 7.99 (d, *J* = 8.0, 1.7 Hz, 1H), 7.87 (d, *J* = 8.0 Hz, 1H), 7.65 (d, *J* = 8.3 Hz, 2H), 7.31 (d, *J* = 8.5 Hz, 2H), 5.15 (t, *J* = 5.6 Hz, 1H), 4.47 (d, *J* = 5.7 Hz, 1H).

##### 2-Chloro-*N*-(3-(chloromethyl)­phenyl)-4-(methylsulfonyl)­benzamide (**8a**)

The synthesis was performed according to GP4 using **5a** (264 mg, 0.777 mmol) and thionyl chloride (2.0 mL) in DCE (10 mL). The product **8a** was obtained in a quantitative yield (281 mg). ^1^H NMR (250 MHz, DMSO-*d*_6_): δ 10.77 (s, 1H), 8.12 (d, *J* = 1.5 Hz, 1H), 8.00 (dd, *J* = 8.0, 1.7 Hz, 1H), 7.89 (d, *J* = 8.0 Hz, 1H), 7.86 (s, 1H), 7.63–7.57 (m, 1H), 7.38 (t, *J* = 7.7 Hz, 1H), 7.21 (d, *J* = 7.9 Hz, 1H), 4.78 (s, 2H), 3.35 (s, 3H).

##### *N*-(3-(Bromomethyl)­benzyl)-2-chloro-4-(methylsulfonyl)­benzamide (**9**)

PBr_3_ (225 μL, 2.37 mmol) was added to a solution of **6** (280 mg, 0.791 mmol) in THF/DCM 1:1 (16.0 mL) at 0 °C, and the resulting solution was stirred for 2 h at 0 °C. After this time, a cold aqueous saturated sodium bicarbonate solution was added to quench the reaction, and the solution was further diluted with water and EtOAc. The phases were separated, and the aqueous phase was extracted with EtOAc 2×. The combined organic layers were dried over magnesium sulfate, filtered, and evaporated to yield the crude product **9** (320 mg, 97%), which was used in the next step without further purification. ^1^H NMR (250 MHz, DMSO-*d*_6_): δ 9.20 (t, *J* = 5.9 Hz, 1H), 8.06–8.04 (m, 1H), 7.95 (dd, *J* = 8.0, 1.7 Hz, 1H), 7.75 (d, *J* = 8.0 Hz, 1H), 7.44–7.27 (m, 4H), 4.71 (s, 2H), 4.48 (d, *J* = 5.9 Hz, 1H), 3.32 (s, 3H).

##### *N*-(3-(Bromomethyl)­phenethyl)-2-chloro-4-(methylsulfonyl)­benzamide (**10**)

PBr_3_ (216 μL, 2.28 mmol) was added to a solution of **7** (280 mg, 0.761 mmol) in THF/DCM 1:1 (16.0 mL) at 0 °C, and the resulting solution was stirred for 2 h at 0 °C. After this time, a cold aqueous saturated sodium bicarbonate solution was added to quench the reaction, and the solution was further diluted with water and EtOAc. The phases were separated, and the aqueous phase was extracted with EtOAc 2×. The combined organic layers were dried over magnesium sulfate, filtered, and evaporated to yield the crude product **10** (323 mg, 98%), which was used in the next step without further purification. ^1^H NMR (250 MHz, DMSO-*d*_6_): δ 8.72 (t, *J* = 5.9 Hz, 1H), 8.03–8.01 (m, 1H), 7.95–7.89 (m, 1H), 7.60 (d, *J* = 8.0 Hz, 1H), 7.39–7.23 (m, 4H), 4.69 (s, 2H), 3.49 (q, *J* = 6.1 Hz, 2H), 3.30 (s, 3H), 2.85 (t, *J* = 7.3 Hz, 2H).

##### 2-Chloro-*N*-(3-(chloromethyl)-2-fluorophenyl)-4-(methylsulfonyl)­benzamide (**8b**)

The synthesis was performed according to GP4 using **5b** (185 mg, 0.517 mmol) and thionyl chloride (1.0 mL) in DCE (5.0 mL). The product **8b** was obtained in a quantitative yield (197 mg). ^1^H NMR (250 MHz, DMSO-*d*_6_): δ 10.64 (s, 1H), 8.12 (d, *J* = 1.5 Hz, 1H), 8.00 (dd, *J* = 8.0, 1.6 Hz, 1H), 7.92–7.84 (m, 2H), 7.40 (td, *J* = 7.8, 2.5 Hz, 1H), 7.25 (t, *J* = 7.8 Hz, 1H), 4.84 (s, 2H), 3.35 (s, 3H).

##### 2-Chloro-*N*-(5-(chloromethyl)-2-fluorophenyl)-4-(methylsulfonyl)­benzamide (**8c**)

The synthesis was performed according to GP4 using **5c** (307 mg, 0.858 mmol) and thionyl chloride (2.0 mL) in DCE (10.0 mL). The product **8c** was obtained in 95% yield (307 mg). ^1^H NMR (250 MHz, DMSO-*d*_6_): δ 10.63 (s, 1H), 8.11 (d, *J* = 1.6 Hz, 1H), 8.03–7.98 (m, 2H), 7.88 (d, *J* = 8.0 Hz, 1H), 7.35–7.31 (m, 2H), 4.81 (s, 2H), 3.34 (s, 3H).

##### 2-Chloro-*N*-(3-(chloromethyl)-5-fluorophenyl)-4-(methylsulfonyl)­benzamide (**8d**)

The synthesis was performed according to GP4 using **5d** (285 mg, 0.797 mmol) and thionyl chloride (2.0 mL) in DCE (10.0 mL). The product **8d** was obtained in 92% yield (275 mg). ^1^H NMR (250 MHz, DMSO-*d*_6_): δ 10.97 (s, 1H), 8.13 (d, *J* = 1.6 Hz, 1H), 8.01 (dd, *J* = 8.0, 1.6 Hz, 1H), 7.91 (d, *J* = 8.0 Hz, 1H), 7.60–7.44 (m, 2H), 7.09 (dt, *J* = 8.9, 1.8 Hz, 1H), 4.78 (s, 2H), 3.34 (s, 3H).

##### 2-Chloro-*N*-(3-(chloromethyl)-4-fluorophenyl)-4-(methylsulfonyl)­benzamide (**8e**)

The synthesis was performed according to GP4 using **5e** (320 mg, 0.894 mmol) and thionyl chloride (2.0 mL) in DCE (10.0 mL). The product **8e** was obtained in 94% yield (315 mg). ^1^H NMR (250 MHz, DMSO-*d*_6_): δ 10.82 (s, 1H), 8.13 (d, *J* = 1.6 Hz, 1H), 8.00 (dd, *J* = 8.0, 1.6 Hz, 1H), 7.94 (dd, *J* = 6.8, 2.7 Hz, 1H), 7.89 (d, *J* = 8.0 Hz, 1H), 7.67–7.60 (m, 1H), 7.28 (t, *J* = 9.7 Hz, 1H), 4.81 (s, 2H), 3.35 (s, 3H).

##### *N*-(3-(Bromomethyl)-4-chlorophenyl)-2-chloro-4-(methylsulfonyl)­benzamide (**8f**)

PBr_3_ (248 μL, 2.61 mmol) was added to a solution of **5f** (326 mg, 0.870 mmol) in THF/DCM 1:1 (16.0 mL) at 0 °C, and the resulting solution was stirred for 2 h at 0 °C. After this time, a cold aqueous saturated sodium bicarbonate solution was added to quench the reaction, and the solution was further diluted with water and EtOAc. The phases were separated, and the aqueous phase was extracted with EtOAc 2×. The combined organic layers were dried over magnesium sulfate, filtered, and evaporated to yield the crude product **8f** (380 mg, quantitative), which was used in the next step without further purification. ^1^H NMR (250 MHz, DMSO-*d*_6_): δ 10.91 (s, 1H), 8.13 (d, *J* = 1.4 Hz, 1H), 8.04–7.98 (m, 2H), 7.92–7.87 (m, 2H), 7.50 (d, *J* = 8.7 Hz, 1H), 5.03 (s, 2H), 3.35 (s, 3H).

##### *N*-(4-Bromo-3-(bromomethyl)­phenyl)-2-chloro-4-(methylsulfonyl)­benzamide (**8g**)

PBr_3_ (239 μL, 2.52 mmol) was added to a solution of **5g** (352 mg, 0.840 mmol) in THF/DCM 1:1 (16.0 mL) at 0 °C, and the resulting solution was stirred for 2 h at 0 °C. After this time, a cold aqueous saturated sodium bicarbonate solution was added to quench the reaction, and the solution was further diluted with water and EtOAc. The phases were separated, and the aqueous phase was extracted with EtOAc 2×. The combined organic layers were dried over magnesium sulfate, filtered, and evaporated to yield the crude product **8g** (404 mg, quantitative), which was used in the next step without further purification. ^1^H NMR (250 MHz, DMSO-*d*_6_): δ 10.91 (s, 1H), 8.12 (d, *J* = 1.4 Hz, 1H), 8.03–7.98 (m, 2H), 7.92–7.87 (m, 2H), 7.68–7.65 (m, 1H), 4.99 (s, 2H), 3.35 (s, 3H).

##### 2-Chloro-*N*-(3-(chloromethyl)-2,4-difluorophenyl)-4-(methylsulfonyl)­benzamide (**8h**)

The synthesis was performed according to GP4 using **5h** (434 mg, 1.15 mmol) and thionyl chloride (3.0 mL) in DCE (15.0 mL). The product **8h** was obtained in a quantitative yield (453 mg). ^1^H NMR (250 MHz, DMSO-*d*_6_): δ 10.64 (s, 1H), 8.11 (d, *J* = 1.6 Hz, 1H), 8.00 (dt, *J* = 7.9, 1.5 Hz, 1H), 7.94–7.85 (m, 2H), 7.24 (td, *J* = 9.0, 1.6 Hz, 1H), 4.82 (s, 2H), 3.34 (s, 3H).

##### 2-Chloro-*N*-(3-(chloromethyl)-4,5-difluorophenyl)-4-(methylsulfonyl)­benzamide (**8i**)

The synthesis was performed according to GP4 using **5i** (570 mg, 1.52 mmol) and thionyl chloride (3.0 mL) in DCE (15.0 mL). The product **8i** was obtained in 77% yield (461 mg). ^1^H NMR (250 MHz, DMSO-*d*_6_): δ 11.03 (s, 1H), 8.13 (s, 1H), 8.03–7.99 (m, 1H), 7.90 (d, *J* = 7.9 Hz, 1H), 7.83–7.77 (m, 1H), 7.67–7.64 (m, 1H), 4.86 (s, 2H), 3.34 (s, 3H).

##### 2-Chloro-*N*-(3-(Chloromethyl)-2,5-difluorophenyl)-4-(methylsulfonyl)­benzamide (**8j**)

The synthesis was performed according to GP4 using **5j** (200 mg, 0.532 mmol, 1.0 equiv) and thionyl chloride (1.5 mL) in DCE (8.0 mL). Purification by flash chromatography (hexane/EtOAc 6:4 to 3:7) gave the product **8j** as a white solid in a yield of 75% (157 mg). ^1^H NMR (250 MHz, DMSO-*d*_6_): δ 10.86 (s, 1H), 8.12 (d, *J* = 1.5 Hz, 1H), 8.00 (dd, *J* = 8.0, 1.7 Hz, 1H), 7.93–7.87 (m, 2H), 7.34–7.27 (m, 1H), 4.81 (s, 2H), 3.35 (s, 3H).

##### 2-Chloro-*N*-(3-(chloromethyl)-2,4,5-trifluorophenyl)-4-(methylsulfonyl)­benzamide (**8k**)

The synthesis was performed according to GP4 using **5k** (603 mg, 1.53 mmol) and thionyl chloride (6.0 mL) in DCE (30.0 mL). The crude product **8k** was obtained as a mixture of starting material and product (1:1, 610 mg). ^1^H NMR (250 MHz, DMSO-*d*_6_): δ 10.85 (d, *J* = 4.1 Hz, 1H), 8.12 (d, *J* = 1.5 Hz, 2H), 8.04–8.01 (m, 1H), 7.90–7.88 (m, 1H), 4.57 (s, 2H), 3.38 (s, 3H).

##### *N*-(4-Bromo-3-(chloromethyl)-2-fluorophenyl)-2-chloro-4-(methylsulfonyl)­benzamide (**8l**)

The synthesis was performed according to GP4 using **5l** (268 mg, 0.615 mmol) and thionyl chloride (2.0 mL) in DCE (10.0 mL). The product **8l** was obtained in 84% yield (235 mg). ^1^H NMR (250 MHz, DMSO-*d*_6_): δ 10.76 (s, 1H), 8.12 (d, *J* = 1.5 Hz, 1H), 8.00 (dd, *J* = 8.0, 1.6 Hz, 1H), 7.96–7.86 (m, 2H), 7.61 (dd, *J* = 8.8 Hz, 1H), 4.86 (d, *J* = 1.9 Hz, 2H), 3.34 (s, 3H).

##### *N*-(3-(Chloromethyl)­phenyl)-4-(methylsulfonyl)­benzamide (**8m**)

The synthesis was performed according to GP4 using **5l** (46.0 mg, 0.151 mmol) and thionyl chloride (1.0 mL) in DCE (5.0 mL). The product **8l** was obtained in a quantitative yield (49.0 mg). ^1^H NMR (250 MHz, DMSO-*d*_6_): δ 10.57 (s, 1H), 8.18 (d, *J* = 8.6 Hz, 2H), 8.08 (d, *J* = 8.6 Hz, 2H), 7.91 (t, *J* = 1.7 Hz, 1H), 7.76–7.70 (m, 1H), 7.38 (t, *J* = 7.9 Hz, 1H), 7.20 (d, *J* = 7.9 Hz, 1H), 4.78 (s, 2H), 3.29 (s, 3H).

##### 2-Chloro-*N*-(4-(chloromethyl)­phenyl)-4-(methylsulfonyl)­benzamide (**48**)

The synthesis was performed according to GP4 using **46** (135 mg, 0.397 mmol) and thionyl chloride (1.0 mL) in DCE (5.0 mL). The product **47** was obtained in quantitative yield (142 mg). ^1^H NMR (250 MHz, DMSO-*d*_6_): δ 10.77 (s, 1H), 8.12 (d, *J* = 1.6 Hz, 1H), 8.00 (dd, *J* = 8.0, 1.7 Hz, 1H), 7.88 (d, *J* = 8.0 Hz, 1H), 7.71 (d, *J* = 8.6 Hz, 2H), 7.44 (d, *J* = 8.6 Hz, 2H), 4.75 (s, 2H), 3.34 (s, 3H).

##### *N*-(3-((4-Bromo-2-formylphenoxy)­methyl)­phenyl)-2-chloro-4-(methylsulfonyl)­benzamide (**11a**)

The synthesis was performed according to GP5 using **8a** (537 mg, 1.50 mmol), 5-bromosalicylaldehyde (362 mg, 1.80 mmol), and potassium carbonate (419 mg, 3.0 mmol) in DMF (15.0 mL). The crude was purified by flash chromatography (hexane/EtOAc, 7:3 to 5:5) to yield 427 mg (54%) of the desired product **11a**. ^1^H NMR (250 MHz, CDCl_3_): δ 10.47 (s, 1H), 8.12 (s, 1H), 7.99 (s, 1H), 7.94 (d, *J* = 2.6 Hz, 1H), 7.88–7.84 (m, 3H), 7.62 (dd, *J* = 8.9, 2.6 Hz, 1H), 7.59 (d, *J* = 7.5 Hz, 1H), 7.44 (t, *J* = 7.9 Hz, 1H), 7.26 (d, *J* = 7.5 Hz, 1H), 6.95 (d, *J* = 8.9 Hz, 1H) 5.21 (s, 2H), 3.08 (s, 3H).

##### *N*-(3-((4-Bromo-2-formylphenoxy)­methyl)­benzyl)-2-chloro-4-(methylsulfonyl)­benzamide (**12**)

The synthesis was performed according to GP5 using **6** (27.9 mg, 0.075 mmol), 5-bromosalicylaldehyde (18.1 mg, 0.090 mmol), and potassium carbonate (20.9 mg, 0.15 mmol) in DMF (3.0 mL). The crude was purified by flash chromatography (hexane/EtOAc, 1:1 to 0:1) to yield 17.2 mg (43%) of the desired product **12**. ^1^H NMR (250 MHz, CDCl_3_): δ 10.43 (s, 1H), 7.99 (d, *J* = 1.6 Hz, 1H), 7.93 (d, *J* = 2.6 Hz, 1H), 7.89 (dd, *J* = 8.0, 1.6 Hz, 1H), 7.83 (d, *J* = 8.0 Hz, 1H), 7.61 (dd, *J* = 8.9, 2.6 Hz, 1H), 7.46–7.34 (m, 4H), 6.94 (d, *J* = 8.9 Hz, 1H), 6.52 (br s, 1H), 5.18 (s, 2H), 4.70 (d, *J* = 5.9 Hz, 2H), 3.07 (s, 3H).

##### *N*-(3-((4-Bromo-2-formylphenoxy)­methyl)­phenethyl)-2-chloro-4-(methylsulfonyl)­benzamide (**13**)

The synthesis was performed according to GP5 using **10** (284 mg, 0.736 mmol), 5-bromosalicylaldehyde (178 mg, 0.883 mmol), and potassium carbonate (205 mg, 1.47 mmol) in DMF (8.0 mL). The crude was purified by flash chromatography (hexane/EtOAc, 3:2 to 0:1) to yield 154 mg (38%) of the desired product **13**. ^1^H NMR (250 MHz, DMSO-*d*_6_): δ 10.32 (s, 1H), 8.71 (t, 5.6 Hz, 1H), 8.00 (d, *J* = 1.7 Hz, 1H), 7.89 (dd, *J* = 8.0, 1.7 Hz, 1H), 7.81 (dd, *J* = 8.8, 2.7 Hz, 1H), 7.76 (d, *J* = 2.6 Hz, 1H), 7.58 (d, *J* = 8.0 Hz, 1H), 7.42 (s, 1H), 7.37–7.31 (m, 3H), 7.30–7.24 (m, 1H), 5.28 (s, 2H), 3.51 (q, *J* = 6.0 Hz, 2H), 3.30 (s, 3H), 2.87 (t, *J* = 7.1 Hz, 2H).

##### *N*-(3-((4-Bromo-2-formylphenoxy)­methyl)-2-fluorophenyl)-2-chloro-4-(methylsulfonyl)­benzamide (**11b**)

The synthesis was performed according to GP5 using **8b** (190 mg, 0.505 mmol), 5-bromosalicylaldehyde (122 mg, 0.606 mmol), and potassium carbonate (141 mg, 1.01 mmol) in DMF (6.0 mL). The crude was purified by flash chromatography (hexane/EtOAc, 7:3 to 5:5) to yield 86.0 mg (32%) of the desired product **11b**. ^1^H NMR (250 MHz, DMSO-*d*_6_): δ 10.63 (s, 1H), 10.28 (s, 1H), 8.11 (d, *J* = 1.6 Hz, 1H), 8.00 (dd, *J* = 8.0, 1.6 Hz, 1H), 7.94–7.83 (m, 3H), 7.78 (d, *J* = 2.6 Hz, 1H), 7.49 (t, *J* = 7.4 Hz, 1H), 7.41 (t, *J* = 8.9 Hz, 1H), 7.29 (t, *J* = 7.6 Hz, 1H), 5.39 (s, 2H), 3.34 (s, 3H).

##### *N*-(5-((4-Bromo-2-formylphenoxy)­methyl)-2-fluorophenyl)-2-chloro-4-(methylsulfonyl)­benzamide (**11c**)

The synthesis was performed according to GP5 using **8c** (300 mg, 0.797 mmol), 5-bromosalicylaldehyde (192 mg, 0.956 mmol), and potassium carbonate (223 mg, 1.59 mmol) in DMF (8.0 mL). The crude was purified by flash chromatography (hexane/EtOAc, 7:3 to 5:5) to yield 211 mg (49%) of the desired product **11c**. ^1^H NMR (250 MHz, DMSO-*d*_6_): δ 10.63 (s, 1H), 10.34 (s, 1H), 8.11 (s, 1H), 8.06 (d, *J* = 7.3 Hz, 1H), 8.00 (d, *J* = 8.0 Hz, 1H), 7.89–7.82 (m, 2H), 7.78 (d, *J* = 2.5 Hz, 1H), 7.45–7.31 (m, 3H), 5.33 (s, 2H), 3.34 (s, 3H).

##### *N*-(3-((4-Bromo-2-formylphenoxy)­methyl)-5-fluorophenyl)-2-chloro-4-(methylsulfonyl)­benzamide (**11d**)

The synthesis was performed according to GP5 using **8d** (235 mg, 0.625 mmol), 5-bromosalicylaldehyde (151 mg, 0.750 mmol), and potassium carbonate (175 mg, 1.25 mmol) in DMF (10.0 mL). The crude was purified by flash chromatography (hexane/EtOAc, 8:2 to 5:5) to yield 86.0 mg (32%) of the desired product **11d**. ^1^H NMR (250 MHz, DMSO-*d*_6_): δ 10.96 (s, 1H), 10.39 (s, 1H), 8.13 (s, 1H), 8.00 (d, *J* = 8.0 Hz, 1H), 7.90 (d, *J* = 8.0 Hz, 1H), 7.84 (dd, *J* = 9.0, 2.5 Hz, 1H), 7.78 (d, *J* = 2.5 Hz, 1H), 7.66 (d, *J* = 10.9 Hz, 1H), 7.56 (s, 1H), 7.29 (d, *J* = 8.8 Hz, 1H), 7.14 (d, *J* = 9.3 Hz, 1H), 5.34 (s, 2H), 3.34 (s, 3H).

##### *N*-(3-((4-Bromo-2-formylphenoxy)­methyl)-4-fluorophenyl)-2-chloro-4-(methylsulfonyl)­benzamide (**11e**)

The synthesis was performed according to GP5 using **8e** (310 mg, 0.824 mmol), 5-bromosalicylaldehyde (199 mg, 0.988 mmol), and potassium carbonate (230 mg, 1.65 mmol) in DMF (8.0 mL). The crude was purified by flash chromatography (hexane/EtOAc, 7:3 to 5:5) to yield 278 mg (62%) of the desired product **11e**. ^1^H NMR (250 MHz, DMSO-*d*_6_): δ 10.80 (s, 1H), 10.34 (s, 1H), 8.13 (d, *J* = 1.6 Hz, 1H), 8.00 (dd, *J* = 8.0, 1.6 Hz, 1H), 7.91–7.77 (m, 5H), 7.40 (d, *J* = 9.0 Hz, 1H), 7.31 (t, *J* = 9.6 Hz, 1H), 5.38 (s, 2H), 3.34 (s, 3H).

##### *N*-(3-((4-Bromo-2-formylphenoxy)­methyl)-4-chlorophenyl)-2-chloro-4-(methylsulfonyl)­benzamide (**11f**)

The synthesis was performed according to GP5 using **8f** (380 mg, 0.870 mmol), 5-bromosalicylaldehyde (210 mg, 1.04 mmol), and potassium carbonate (243 mg, 1.74 mmol) in DMF (8.0 mL). The crude was purified by flash chromatography (hexane/EtOAc, 8:2 to 5:5) to yield 29.4 mg (6.1%) of the desired product **11f**. ^1^H NMR (250 MHz, DMSO-*d*_6_): δ 10.89 (s, 1H), 10.41 (s, 1H), 8.13 (d, *J* = 1.6 Hz, 1H), 8.01 (dd, *J* = 8.0, 1.6 Hz, 1H), 7.94 (d, *J* = 2.5 Hz, 1H), 7.92–7.82 (m, 3H), 7.79 (d, *J* = 2.7 Hz, 1H), 7.55 (d, *J* = 8.7 Hz, 1H), 7.37 (d, *J* = 9.0 Hz, 1H), 5.36 (s, 2H), 3.34 (s, 3H).

##### *N*-(4-Bromo-3-((4-bromo-2-formylphenoxy)­methyl)­phenyl)-2-chloro-4-(methylsulfonyl)­benzamide (**11g**)

The synthesis was performed according to GP5 using **8g** (405 mg, 0.840 mmol), 5-bromosalicylaldehyde (203 mg, 1.01 mmol), and potassium carbonate (235 mg, 1.68 mmol) in DMF (8.0 mL). The crude was purified by flash chromatography (hexane/EtOAc, 8:2 to 5:5) to yield 38.6 mg (7.6%) of the desired product **11g**. ^1^H NMR (250 MHz, DMSO-*d*_6_): δ 10.89 (s, 1H), 10.41 (s, 1H), 8.13 (d, *J* = 1.6 Hz, 1H), 8.01 (dd, *J* = 8.0, 1.6 Hz, 1H), 7.94–7.84 (m, 3H), 7.81–7.76 (m, 2H), 7.70 (d, *J* = 8.7 Hz, 1H), 7.35 (d, *J* = 8.9 Hz, 1H), 5.31 (s, 2H), 3.34 (s, 3H).

##### *N*-(3-((4-Bromo-2-formylphenoxy)­methyl)-2,4-difluorophenyl)-2-chloro-4-(methylsulfonyl)­benzamide (**11h**)

The synthesis was performed according to GP5 using **8h** (220 mg, 0.558 mmol), 5-bromosalicylaldehyde (134 mg, 0.669 mmol), and potassium carbonate (156 mg, 1.12 mmol) in DMF (6.0 mL). The crude was purified by flash chromatography (hexane/EtOAc, 7:3 to 5:5) to yield 99.8 mg (32%) of the desired product **11h**. ^1^H NMR (250 MHz, DMSO-*d*_6_): δ 10.64 (s, 1H), 10.16 (s, 1H), 8.11 (d, *J* = 1.6 Hz, 1H), 8.00 (dd, *J* = 8.0, 1.6 Hz, 1H), 7.97–7.87 (m, 3H), 7.76 (d, *J* = 3.6 Hz, 1H), 7.48 (d, *J* = 8.9 Hz, 1H), 7.27 (t, *J* = 9.0 Hz, 1H), 5.39 (s, 2H), 3.34 (s, 3H).

##### *N*-(3-((4-Bromo-5-fluoro-2-formylphenoxy)­methyl)-4,5-difluorophenyl)-2-chloro-4-(methylsulfonyl)­benzamide (**11i**)

The synthesis was performed according to GP5 using **8i** (450 mg, 1.14 mmol), 5-bromo-4-fluoro-2-hydroxybenzaldehyde (300 mg, 1.37 mmol), and potassium carbonate (318 mg, 2.28 mmol) in DMF (10.0 mL). The crude was purified by flash chromatography (hexane/EtOAc, 8:2 to 5:5) to yield 101 mg (15%) of the desired product **11i**. ^1^H NMR (250 MHz, DMSO-*d*_6_): δ 10.99 (s, 1H), 10.27 (s, 1H), 8.14 (s, 1H), 8.04–7.89 (m, 5H), 7.62 (d, *J* = 10.2 Hz, 1H), 5.44 (s, 2H), 3.36 (s, 3H).

##### *N*-(3-((4-Bromo-5-fluoro-2-formylphenoxy)­methyl)-2,5-difluorophenyl)-2-chloro-4-(methylsulfonyl)­benzamide (**11j**)

The synthesis was performed according to GP5 using **8j** (150 mg, 0.380 mmol), 5-bromo-4-fluoro-2-hydroxybenzaldehyde (99.9 mg, 0.456 mmol), and potassium carbonate (106 mg, 0.76 mmol) in DMF (6.0 mL). The crude was purified by flash chromatography (hexane/EtOAc, 7:3 to 5:5) to yield 50.9 mg (23%) of the desired product **11j**. ^1^H NMR (250 MHz, DMSO-*d*_6_): δ 10.86 (s, 1H), 10.21 (s, 1H), 8.11 (s, 1H), 8.02–7.98 (m, 2H), 7.92–7.87 (m, 2H), 7.61 (d, *J* = 10.8 Hz, 1H), 7.45–7.38 (m, 1H), 5.39 (s, 2H), 3.34 (s, 3H).

##### *N*-(3-((4-Bromo-5-fluoro-2-formylphenoxy)­methyl)-2,4,5-trifluorophenyl)-2-chloro-4-(methylsulfonyl)­benzamide (**11k**)

The synthesis was performed according to GP5 using **8k** (320 mg, 0.776 mmol), 5-bromo-4-fluoro-2-hydroxybenzaldehyde (204 mg, 0.931 mmol), and potassium carbonate (217 mg, 1.55 mmol) in DMF (10.0 mL). The crude was purified by flash chromatography (hexane/EtOAc, 8:2 to 3:7) to yield 75.6 mg (16%) of the desired product **11k**. ^1^H NMR (250 MHz, DMSO-*d*_6_): δ 10.87 (s, 1H), 10.10 (s, 1H), 8.21–8.10 (m, 1H), 8.12 (d, *J* = 1.6 Hz, 1H), 8.01 (dd, *J* = 8.0, 1.6 Hz, 1H), 7.94 (d, *J* = 8.2 Hz, 1H), 7.88 (d, *J* = 8.0 Hz, 1H), 7.68 (d, *J* = 10.9 Hz, 1H), 5.46 (s, 2H), 3.34 (s, 3H).

##### *N*-(4-Bromo-3-((4-bromo-5-fluoro-2-formylphenoxy)­methyl)-2-fluorophenyl)-2-chloro-4-(methylsulfonyl)­benzamide (**11l**)

The synthesis was performed according to GP5 using **8l** (235 mg, 0.516 mmol), 5-bromo-4-fluoro-2-hydroxybenzaldehyde (136 mg, 0.619 mmol), and potassium carbonate (144 mg, 1.03 mmol) in DMF (10.0 mL). The crude was purified by flash chromatography (hexane/EtOAc, 8:2 to 5:5) to yield 48.1 mg (15%) of the desired product **11l**. ^1^H NMR (300 MHz, DMSO-*d*_6_): δ 10.77 (s, 1H), 10.10 (s, 1H), 8.12 (d, *J* = 1.6 Hz, 1H), 8.00 (dd, *J* = 8.0, 1.6 Hz, 1H), 7.94 (d, *J* = 8.3 Hz, 1H), 7.89 (d, *J* = 8.0 Hz, 1H), 7.71 (d, *J* = 10.8 Hz, 1H), 7.66 (d, *J* = 8.9 Hz, 1H), 5.41 (s, 2H), 3.34 (s, 3H).

##### *N*-(3-((4-Bromo-2-formylphenoxy)­methyl)­phenyl)-4-(methylsulfonyl)­benzamide (**11m**)

The synthesis was performed according to GP5 using **8m** (48.9 mg, 0.151 mmol), 5-bromosalicylaldehyde (36.4 mg, 0.181 mmol), and potassium carbonate (42.2 mg, 0.302 mmol) in DMF (4.0 mL). The crude was purified by flash chromatography (hexane/EtOAc, 8:2 to 5:5) to yield 42.8 mg (58%) of the desired product **11m**. ^1^H NMR (250 MHz, CDCl_3_): δ 10.46 (s, 1H), 8.52 (s, 1H), 8.03–7.79 (m, 6H), 8.06 (d, *J* = 7.3 Hz, 1H), 7.68–7.63 (m, 1H), 7.60 (dd, *J* = 8.9, 2.6 Hz, 1H), 7.41 (t, *J* = 7.9 Hz, 1H), 7.22 (d, *J* = 7.7 Hz, 1H), 6.94 (d, *J* = 8.9 Hz, 1H), 5.19 (s, 2H), 3.07 (s, 3H).

##### *N*-(3-((4-Bromo-5-fluoro-2-formylphenoxy)­methyl)­phenyl)-2-chloro-4-(methylsulfonyl)­benzamide (**11n**)

The synthesis was performed according to GP5 using **8a** (136 mg, 0.380 mmol), 5-bromo-4-fluoro-2-hydroxybenzaldehyde (99.9 mg, 0.456 mmol), and potassium carbonate (106 mg, 0.76 mmol) in DMF (8.0 mL). The crude was purified by flash chromatography (hexane/EtOAc, 8:2 to 5:5) to yield 120 mg (58%) of the desired product **11n**. ^1^H NMR (250 MHz, DMSO-*d*_6_): δ 10.76 (s, 1H), 10.29 (s, 1H), 8.12 (d, *J* = 1.6 Hz, 1H), 8.00 (dd, *J* = 8.0, 1.6 Hz, 1H), 7.94 (d, *J* = 8.3 Hz, 1H), 7.89 (d, *J* = 8.0 Hz, 1H), 7.85 (s, 1H), 7.71 (d, *J* = 8.9 Hz, 1H), 7.53 (d, *J* = 11.1 Hz, 1H), 7.43 (t, *J* = 7.9 Hz, 1H), 7.28 (d, *J* = 7.6 Hz, 1H), 5.35 (s, 2H), 3.34 (s, 3H).

##### *N*-(3-((4-Bromo-5-fluoro-2-formylphenoxy)­methyl)-5-fluorophenyl)-2-chloro-4-(methylsulfonyl)­benzamide (**11o**)

The synthesis was performed according to GP5 using **8d** (400 mg, 1.06 mmol), 5-bromo-4-fluoro-2-hydroxybenzaldehyde (278 mg, 1.27 mmol), and potassium carbonate (296 mg, 2.12 mmol) in DMF (10.0 mL). The crude was purified by flash chromatography (hexane/EtOAc, 7:3 to 5:5) to yield 214 mg (36%) of the desired product **11o**. ^1^H NMR (250 MHz, DMSO-*d*_6_): δ 10.97 (s, 1H), 10.30 (s, 1H), 8.13 (d, *J* = 1.6 Hz, 1H), 8.01 (dd, *J* = 8.0, 1.7 Hz, 1H), 7.95 (d, *J* = 8.2 Hz, 1H), 7.90 (d, *J* = 8.0 Hz, 1H), 7.66 (dt, *J* = 11.2, 2.1 Hz, 1H), 7.56 (s, 1H), 7.49 (d, *J* = 11.0 Hz, 1H), 7.18–7.13 (m, 1H), 5.34 (s, 2H), 3.34 (s, 3H).

##### *N*-(3-((4-Bromo-5-fluoro-2-formylphenoxy)­methyl)-2,4-difluorophenyl)-2-chloro-4-(methylsulfonyl)­benzamide (**11p**)

The synthesis was performed according to GP5 using **8h** (560 mg, 1.42 mmol), 5-bromo-4-fluoro-2-hydroxybenzaldehyde (373 mg, 1.70 mmol), and potassium carbonate (396 mg, 2.84 mmol) in DMF (10.0 mL). The crude was purified by flash chromatography (hexane/EtOAc, 7:3 to 5:5) to yield 124 mg (15%) of the desired product **11p**. ^1^H NMR (250 MHz, DMSO-*d*_6_): δ 10.65 (s, 1H), 10.08 (s, 1H), 8.11 (d, *J* = 1.6 Hz, 1H), 8.01 (dd, *J* = 8.0, 1.6 Hz, 1H), 7.93–7.87 (m, 3H), 7.68 (d, *J* = 10.9 Hz, 1H), 7.23 (t, *J* = 9.1 Hz, 1H), 5.41 (s, 2H), 3.34 (s, 3H).

##### *N*-(3-((4-Bromo-2-formyl-5-methoxyphenoxy)­methyl)­phenyl)-2-chloro-4-(methylsulfonyl)­benzamide (**11q**)

The synthesis was performed according to GP5 using **8a** (140 mg, 0.391 mmol), 5-bromo-2-hydroxy-4-methoxybenzaldehyde (108 mg, 0.469 mmol), and potassium carbonate (109 mg, 0.782 mmol) in DMF (8.0 mL). The crude was purified by flash chromatography (hexane/EtOAc, 6:4 to 3:7) to yield 161 mg (74%) of the desired product **11q**. ^1^H NMR (250 MHz, DMSO-*d*_6_): δ 10.76 (s, 1H), 10.23 (s, 1H), 8.13 (d, *J* = 1.6 Hz, 1H), 8.00 (dd, *J* = 8.0, 1.6 Hz, 1H), 7.91–7.87 (m, 2H), 7.81 (s, 1H), 7.68 (d, *J* = 8.5 Hz, 1H), 7.43 (t, *J* = 7.8 Hz, 1H), 7.29 (d, *J* = 7.6 Hz, 1H), 7.05 (s, 1H), 5.39 (s, 2H), 4.00 (s, 3H), 3.34 (s, 3H).

##### *N*-(3-((4-Bromo-2-fluoro-6-formylphenoxy)­methyl)-2,4,5-trifluorophenyl)-2-chloro-4-(methylsulfonyl)­benzamide (**11r**)

The synthesis was performed according to GP5 using **8k** (294 mg, 0.712 mmol), 5-bromo-3-fluoro-2-hydroxybenzaldehyde (187 mg, 0.854 mmol), and potassium carbonate (199 mg, 1.42 mmol) in DMF (7.5 mL). The crude was purified by flash chromatography (hexane/EtOAc 9:1 to 1:1) to yield 153 mg (36%) of the desired product **11r**. ^1^H NMR (400 MHz, DMSO-*d*_6_): δ 10.85 (s, 1H), 10.06 (s, 1H), 8.17–8.10 (m, 1H), 8.12 (d, *J* = 1.6 Hz, 1H), 8.06 (dd, *J* = 10.7, 2.4 Hz, 1H), 8.01 (dd, *J* = 8.0, 1.7 Hz, 1H), 7.88 (d, *J* = 8.0 Hz, 1H), 7.66 (dd, *J* = 2.4, 1.5 Hz, 1H), 5.42 (s, 2H), 3.35 (s, 3H).

##### *N*-(4-((4-Bromo-2-formylphenoxy)­methyl)­phenyl)-2-chloro-4-(methylsulfonyl)­benzamide (**49**)

The synthesis was performed according to GP5 using **47** (136 mg, 0.380 mmol), 5-bromosalicylaldehyde (91.7 mg, 0.456 mmol), and potassium carbonate (106 mg, 0.760 mmol) in DMF (5.0 mL). The crude was purified by flash chromatography (hexane/EtOAc, 5:1 to 0:1) to yield 427 mg (54%) of the desired product **48**. ^1^H NMR (250 MHz, CDCl_3_): δ 10.75 (s, 1H), 10.32 (s, 1H), 8.12 (d, *J* = 1.6 Hz, 1H), 8.00 (dd, *J* = 8.0, 1.7 Hz, 1H), 7.88 (d, *J* = 8.0 Hz, 1H), 7.82 (dd, *J* = 8.9, 2.7 Hz, 1H), 7.77–7.71 (m, 3H), 7.52 (d, *J* = 8.5 Hz, 2H), 7.33 (d, *J* = 8.9 Hz, 1H), 5.28 (s, 2H), 3.34 (s, 3H).

##### *N*-(3-((4-Bromo-2-(((2-hydroxyethyl)­amino)­methyl)­phenoxy)­methyl)­phenyl)-2-chloro-4-(methylsulfonyl)­benzamide (**14**)

The synthesis was performed according to GP6 using **11a** (60 mg, 0.115 mmol), ethanolamine (21.0 μL, 0.345 mmol), acetic acid (39.5 μL, 0.690 mmol), and sodium cyanoborohydride (22.8 mg, 0.345 mmol) in DMF (5.0 mL). The crude was purified by preparative HPLC to yield 38.5 mg (30%) of the desired product **14**. ^1^H NMR (300 MHz, CDCl_3_): δ 10.20 (s, 1H), 8.15 (d, *J* = 8.4 Hz, 1H), 7.94 (s, 1H), 7.79 (d, *J* = 8.1 Hz, 1H), 7.71 (d, *J* = 8.0 Hz, 1H), 7.55 (s, 1H), 7.45–7.38 (m, 3H), 7.07 (d, *J* = 7.7 Hz, 1H), 6.85 (d, *J* = 8.7 Hz, 1H), 5.59 (br s, 2H), 5.07 (s, 2H), 4.05 (s, 2H), 3.57 (br s, 2H), 3.05 (s, 3H), 2.85 (br s, 2H); ^13^C NMR (75 MHz, CDCl_3_): δ 164.0, 155.8, 142.6, 141.3, 139.0, 136.5, 134.3, 133.8, 132.5, 130.2, 129.5, 128.8, 125.7, 122.7, 122.1, 120.0, 118.0, 113.7, 113.4, 69.7, 57.2, 48.7, 45.9, 44.3; *R*_*f*_ HPLC: 7.8 min (13 min from 10 to 95% MeCN in water (0.1% formic acid), then 7 min 95% MeCN). 95.5% purity; HRMS (MALDI): *m*/*z* found, 567.0350 [M + H]^+^ (calcd C_24_H_25_BrClN_2_O_5_S^+^, 567.0351).

##### *N*-(3-((4-Bromo-2-(((2-hydroxyethyl)­amino)­methyl)­phenoxy)­methyl)­benzyl)-2-chloro-4-(methylsulfonyl)­benzamide (**15**)

The synthesis was performed according to GP6 using **12** (110 mg, 0.205 mmol), ethanolamine (37.5 μL, 0.615 mmol), acetic acid (70.4 μL, 1.23 mmol), and sodium cyanoborohydride (40.7 mg, 0.615 mmol) in DMF (7.0 mL). The crude was purified by preparative HPLC to yield 54.7 mg (43%) of the desired product **15** as a formate salt. ^1^H NMR (300 MHz, DMSO-*d*_6_): δ 9.23 (t, *J* = 5.8 Hz, 1H), 8.25 (br s, 1H), 8.06 (d, *J* = 1.4 Hz, 1H), 7.94 (dd, *J* = 8.0, 1.6 Hz, 1H), 7.71 (d, *J* = 8.0 Hz, 1H), 7.58 (d, *J* = 2.4 Hz, 1H), 7.47–7.33 (m, 5H), 7.06 (d, *J* = 8.9 Hz, 1H), 5.19 (br s, 2H), 5.16 (s, 2H), 4.51 (d, *J* = 5.9 Hz, 2H), 3.88 (s, 2H), 3.52 (t, *J* = 5.5 Hz, 2H), 3.32 (s, 3H), 2.70 (t, *J* = 5.4 Hz, 2H); ^13^C NMR (75 MHz, CDCl_3_): δ 165.3, 164.5, 155.5, 142.7, 141.3, 139.1, 136.9, 132.0, 131.0, 130.9, 129.8, 128.7, 128.6, 128.0, 126.8, 126.2, 126.1, 125.7, 114.3, 112.1, 69.7, 59.0, 50.2, 45.9, 43.1, 42.4; *R*_*f*_ HPLC: 7.7 min (13 min from 10 to 95% MeCN in water (0.1% formic acid), then 7 min 95% MeCN). 96.2% purity; HRMS (MALDI): *m*/*z* found, 581.0507 [M + H]^+^ (calcd C_25_H_27_BrClN_2_O_5_S^+^ 581.0507).

##### *N*-(3-((4-Bromo-2-(((2-hydroxyethyl)­amino)­methyl)­phenoxy)­methyl)­phenethyl)-2-chloro-4-(methylsulfonyl)­benzamide (**16**)

The synthesis was performed according to GP6 using **13** (150 mg, 0.272 mmol), ethanolamine (50.3 μL, 0.816 mmol), acetic acid (92.3 μL, 1.63 mmol), and sodium cyanoborohydride (54.0 mg, 0.816 mmol) in DMF (8.0 mL). The crude was purified by preparative HPLC to yield 79.7 mg (46%) of the desired product **16** as a formate salt. ^1^H NMR (300 MHz, DMSO-*d*_6_): δ 8.77 (t, *J* = 5.3 Hz, 1H), 8.28 (br s, 1H), 8.03 (d, *J* = 1.3 Hz, 1H), 7.91 (dd, *J* = 8.0, 1.4 Hz, 1H), 7.61–7.58 (m, 2H), 7.44 (dd, *J* = 8.8, 2.4 Hz, 1H), 7.39–7.32 (m, 3H), 7.27–7.23 (m, 1H), 7.07 (d, *J* = 8.8 Hz, 1H), 6.27 (br s, 2H), 5.14 (s, 2H), 3.92 (s, 2H), 3.57–3.48 (m, 4H), 3.30 (s, 3H), 2.88 (t, *J* = 7.1 Hz, 2H), 2.74 (t, *J* = 5.1 Hz, 2H); ^13^C NMR (75 MHz, CDCl_3_): δ 165.1, 164.8, 155.6, 142.6, 141.5, 139.4, 136.7, 132.3, 131.3, 130.9, 129.7, 128.5, 128.4, 128.0, 127.8, 125.7, 125.3, 114.4, 112.0, 69.8, 58.6, 50.0, 45.6, 43.1, 40.4, 34.7; *R*_*f*_ HPLC: 7.7 min (13 min from 10 to 95% MeCN in water (0.1% formic acid), then 7 min 95% MeCN). 95.8% purity; HRMS (MALDI): *m*/*z* found, 595.0646 [M + H]^+^ (calcd C_26_H_29_BrClN_2_O_5_S^+^, 595.0664).

##### *N*-(3-((4-Bromo-2-(((2-hydroxyethyl)­amino)­methyl)­phenoxy)­methyl)-2-fluorophenyl)-2-chloro-4-(methylsulfonyl)­benzamide (**17**)

The synthesis was performed according to GP6 using **11b** (85.0 mg, 0.157 mmol), ethanolamine (38.3 μL, 0.628 mmol), acetic acid (53.9 μL, 0.942 mmol), and sodium cyanoborohydride (41.5 mg, 0.628 mmol) in DMF (8.0 mL). The crude was purified by preparative HPLC to yield 47.0 mg (51%) of the desired product **17** as a formate salt. ^1^H NMR (500 MHz, DMSO-*d*_6_): δ 10.64 (s, 1H), 8.26 (s, 1H), 8.12 (d, *J* = 1.6 Hz, 1H), 8.00 (dd, *J* = 8.0, 1.6 Hz, 1H), 7.90–7.87 (m, 2H), 7.57 (d, *J* = 2.6 Hz, 1H), 7.46–7.42 (m, 2H), 7.28 (t, *J* = 7.9 Hz, 1H), 7.13 (d, *J* = 8.8 Hz, 1H), 5.23 (s, 2H), 3.82 (s, 2H), 3.50 (t, *J* = 5.7 Hz, 2H), 3.35 (s, 3H), 2.66 (t, *J* = 5.7 Hz, 2H); ^13^C NMR (125 MHz, DMSO-*d*_6_): δ 164.4, 164.2, 155.2, 152.5 (d, *J* = 248.0 Hz), 143.0, 140.9, 131.9, 131.0, 130.8, 130.0, 129.5, 128.0, 128.0, 126.6 (d, *J* = 2.6 Hz), 125.8, 125.2 (d, *J* = 12.1 Hz), 125.1, 124.3 (d, *J* = 13.1 Hz), 124.2 (d, *J* = 4.1 Hz), 114.4, 112.4, 64.1, 59.3 50.4, 46.0, 43.1; ^19^F NMR (471 MHz, DMSO-*d*_6_): δ −128.0; *R*_*f*_ HPLC: 7.7 min (13 min from 10 to 95% MeCN in water (0.1% formic acid), then 7 min 95% MeCN). 98.6% purity; HRMS (MALDI): *m*/*z* found, 585.0258 [M + H]^+^ (calcd C_24_H_24_BrClFN_2_O_5_S^+^ 585.0256).

##### *N*-(5-((4-Bromo-2-(((2-hydroxyethyl)­amino)­methyl)­phenoxy)­methyl)-2-fluorophenyl)-2-chloro-4-(methylsulfonyl)­benzamide (**18**)

The synthesis was performed according to GP6 using **11c** (200 mg, 0.370 mmol), ethanolamine (90.2 μL, 1.48 mmol), acetic acid (127 μL, 2.22 mmol), and sodium cyanoborohydride (97.9 mg, 1.48 mmol) in DMF (12.0 mL). The crude was purified by preparative HPLC to yield 72.1 mg (33%) of the desired product **18** as a formate salt. ^1^H NMR (500 MHz, DMSO-*d*_6_): δ 10.66 (s, 1H), 8.26 (s, 1H), 8.12 (d, *J* = 1.6 Hz, 1H), 8.04 (d, *J* = 7.3, 1.6 Hz, 1H), 8.00 (dd, *J* = 8.0, 1.6 Hz, 1H), 7.87 (d, *J* = 8.0 Hz, 1H), 7.58 (d, *J* = 2.5 Hz, 1H), 7.45 (dd, *J* = 8.7, 2.5 Hz, 1H), 7.39–7.32 (m, 2H), 7.08 (d, *J* = 8.8 Hz, 1H), 5.17 (s, 2H), 3.89 (s, 2H), 3.53 (t, *J* = 5.7 Hz, 2H), 3.35 (s, 3H), 2.72 (t, *J* = 5.7 Hz, 2H); ^13^C NMR (125 MHz, DMSO-*d*_6_): δ 164.6, 164.2, 155.3, 153.8 (d, *J* = 245.8 Hz), 143.0, 140.8, 133.1 (d, *J* = 3.2 Hz), 132.1, 131.04, 131.03, 130.0, 128.8, 128.0, 125.8, 125.7 (d, *J* = 7.6 Hz), 125.2 (d, *J* = 12.4 Hz), 124.1, 115.9 (d, *J* = 19.7 Hz), 114.4, 112.2, 68.9, 59.0 50.3, 45.9, 43.1; ^19^F NMR (471 MHz, DMSO-*d*_6_): δ −123.9; *R*_*f*_ HPLC: 7.8 min (13 min from 10 to 95% MeCN in water (0.1% formic acid), then 7 min 95% MeCN). 95.5% purity; HRMS (MALDI): *m*/*z* found, 585.0267 [M + H]^+^ (cal. C_24_H_24_BrClFN_2_O_5_S^+^ 585.0256).

##### *N*-(3-((4-Bromo-2-(((2-hydroxyethyl)­amino)­methyl)­phenoxy)­methyl)-5-fluorophenyl)-2-chloro-4-(methylsulfonyl)­benzamide (**19**)

The synthesis was performed according to GP6 using **11d** (35.0 mg, 0.0647 mmol), ethanolamine (11.8 μL, 0.194 mmol), acetic acid (23.3 μL, 0.388 mmol), and sodium cyanoborohydride (12.8 mg, 0.194 mmol) in DMF (3.0 mL). The crude was purified by preparative HPLC to yield 10.8 mg (29%) of the desired product **19** as a formate salt. ^1^H NMR (500 MHz, DMSO-*d*_6_): δ 10.97 (s, 1H), 8.18 (s, 1H), 8.13 (d, *J* = 1.6 Hz, 1H), 8.01 (dd, *J* = 8.0, 1.6 Hz, 1H), 7.89 (d, *J* = 8.0 Hz, 1H), 7.61–7.53 (m, 3H), 7.39 (dd, *J* = 8.7, 2.6 Hz, 1H), 7.08 (d, *J* = 9.3 Hz, 1H), 7.01 (d, *J* = 8.8 Hz, 1H), 5.18 (s, 2H), 3.82 (s, 2H), 3.49 (t, *J* = 5.7 Hz, 2H), 3.34 (s, 3H), 2.63 (t, *J* = 5.7 Hz, 2H); ^13^C NMR (125 MHz, DMSO-*d*_6_): δ 164.0, 163.7, 162.1 (d, *J* = 240.6 Hz), 155.0, 143.2, 140.8, 140.3 (d, *J* = 9.0 Hz), 140.2 (d, *J* = 11.4 Hz), 131.5, 130.9, 130.8, 130.4, 129.9, 128.1, 125.9, 114.2, 113.7, 112.3, 109.3 (d, *J* = 22.3 Hz), 105.7 (d, *J* = 26.1 Hz), 68.7, 59.9, 50.8, 46.5, 43.1; ^19^F NMR (471 MHz, DMSO-*d*_6_): δ −111.7; *R*_*f*_ HPLC: 8.0 min (13 min from 10 to 95% MeCN in water (0.1% formic acid), then 7 min 95% MeCN). 95.4% purity; HRMS (MALDI): *m*/*z* found, 585.0259 [M + H]^+^ (calcd C_24_H_24_BrClFN_2_O_5_S^+^ 585.0256).

##### *N*-(3-((4-Bromo-2-(((2-hydroxyethyl)­amino)­methyl)­phenoxy)­methyl)-4-fluorophenyl)-2-chloro-4-(methylsulfonyl)­benzamide (**20**)

The synthesis was performed according to GP6 using **11e** (100 mg, 0.185 mmol), ethanolamine (33.8 μL, 0.555 mmol), acetic acid (63.5 μL, 1.11 mmol), and sodium cyanoborohydride (36.7 mg, 0.555 mmol) in DMF (8.0 mL). The crude was purified by preparative HPLC to yield 67.0 mg (62%) of the desired product **20** as a formate salt. ^1^H NMR (500 MHz, DMSO-*d*_6_): δ 10.93 (s, 1H), 8.19 (s, 1H), 8.12 (s, 1H), 8.00 (d, *J* = 8.0 Hz, 1H), 7.93 (dd, *J* = 6.3, 2.1 Hz, 1H), 7.88 (d, *J* = 8.0 Hz, 1H), 7.76–7.72 (m, 1H), 7.59 (d, *J* = 2.2 Hz, 1H), 7.45 (dd, *J* = 8.7, 2.2 Hz, 1H), 7.28 (t, *J* = 9.3 Hz, 1H), 7.12 (d, *J* = 8.8 Hz, 1H), 5.21 (s, 2H), 3.87 (s, 2H), 3.50 (t, *J* = 5.7 Hz, 2H), 3.34 (s, 3H), 2.69 (t, *J* = 5.7 Hz, 2H); ^13^C NMR (125 MHz, DMSO-*d*_6_): δ 164.8, 163.7, 156.3 (d, *J* = 241.8 Hz), 155.2, 143.0, 141.0, 135.0 (d, *J* = 2.5 Hz), 132.0, 131.1, 131.0, 129.9, 128.9, 128.1, 125.9, 124.0 (d, *J* = 15.5 Hz), 121.1 (d, *J* = 7.7 Hz), 120.8 (d, *J* = 3.4 Hz), 115.7 (d, *J* = 22.1 Hz), 114.4, 112.5, 64.0, 59.1, 50.1, 45.6, 43.1; ^19^F NMR (471 MHz, DMSO-*d*_6_): δ −123.2; *R*_*f*_ HPLC: 8.0 min (13 min from 10 to 95% MeCN in water (0.1% formic acid), then 7 min 95% MeCN). 98.1% purity; HRMS (MALDI): *m*/*z* found, 585.0253 [M + H]^+^ (calcd C_24_H_24_BrClFN_2_O_5_S^+^ 585.0256).

##### *N*-(3-((4-Bromo-2-(((2-hydroxyethyl)­amino)­methyl)­phenoxy)­methyl)-4-chlorophenyl)-2-chloro-4-(methylsulfonyl)­benzamide (**21**)

The synthesis was performed according to GP6 using **11f** (27.9 mg, 0.050 mmol), ethanolamine (9.20 μL, 0.150 mmol), acetic acid (17.2 μL, 0.30 mmol), and sodium cyanoborohydride (9.92 mg, 0.150 mmol) in DMF (3.0 mL). The crude was purified by preparative HPLC to yield 4.9 mg (16%) of the desired product **21** as a formate salt. ^1^H NMR (500 MHz, DMSO-*d*_6_): δ 10.95 (s, 1H), 8.17 (s, 1H), 8.12 (d, *J* = 1.6 Hz, 1H), 8.00 (dd, *J* = 8.0, 1.6 Hz, 1H), 7.98 (d, *J* = 8.0 Hz, 1H), 7.89 (d, *J* = 8.0 Hz, 1H), 7.75 (dd, *J* = 8.7, 2.6 Hz, 1H), 7.56 (d, *J* = 2.4 Hz, 1H), 7.53 (d, *J* = 8.7 Hz, 1H), 7.43 (dd, *J* = 8.7, 2.5 Hz, 1H), 7.06 (d, *J* = 8.8 Hz, 1H), 5.19 (s, 2H), 3.83 (s, 2H), 3.47 (t, *J* = 5.7 Hz, 2H), 3.34 (s, 3H), 2.63 (t, *J* = 5.7 Hz, 2H); ^13^C NMR (125 MHz, DMSO-*d*_6_): δ 163.9, 163.8, 155.1, 143.1, 140.9, 137.8, 134.7, 131.6, 130.9, 130.6, 130.5, 129.9, 129.8, 128.1, 126.7, 125.9, 120.6, 120.2, 114.2, 112.5, 67.2, 59.7, 50.6, 46.2, 43.1; *R*_*f*_ HPLC: 8.3 min (13 min from 10 to 95% MeCN in water (0.1% formic acid), then 7 min 95% MeCN). 99.4% purity; HRMS (MALDI): *m*/*z* found, 600.9962 [M + H]^+^ (calcd C_24_H_24_BrCl_2_N_2_O_5_S^+^ 600.9961).

##### *N*-(4-Bromo-3-((4-bromo-2-(((2-hydroxyethyl)­amino)­methyl)­phenoxy)­methyl)­phenyl)-2-chloro-4-(methylsulfonyl)­benzamide (**22**)

The synthesis was performed according to GP6 using **11g** (36.1 mg, 0.060 mmol), ethanolamine (11.0 μL, 0.180 mmol), acetic acid (20.6 μL, 0.36 mmol), and sodium cyanoborohydride (11.9 mg, 0.180 mmol) in DMF (3.0 mL). The crude was purified by preparative HPLC to yield 8.7 mg (22%) of the desired product **22** as a formate salt. ^1^H NMR (500 MHz, DMSO-*d*_6_): δ 11.00 (s, 1H), 8.16 (s, 1H), 8.12 (d, *J* = 1.6 Hz, 1H), 8.00 (dd, *J* = 8.0, 1.6 Hz, 1H), 7.97 (d, *J* = 8.0 Hz, 1H), 7.89 (d, *J* = 8.0 Hz, 1H), 7.72–7.68 (m, 2H), 7.58 (d, *J* = 2.5 Hz, 1H), 7.45 (dd, *J* = 8.7, 2.5 Hz, 1H), 7.05 (d, *J* = 8.8 Hz, 1H), 5.14 (s, 2H), 3.88 (s, 2H), 3.48 (t, *J* = 5.7 Hz, 2H), 3.34 (s, 3H), 2.66 (t, *J* = 5.7 Hz, 2H); ^13^C NMR (125 MHz, DMSO-*d*_6_): δ 164.1, 163.9, 155.1, 143.1, 140.9, 138.4, 136.3, 133.0, 131.8, 130.9, 130.8, 129.9, 129.7, 128.1, 125.9, 120.8, 120.4, 116.1, 114.2, 112.5, 69.4, 59.4, 50.3, 45.9, 43.1; *R*_*f*_ HPLC: 8.4 min (13 min from 10 to 95% MeCN in water (0.1% formic acid), then 7 min 95% MeCN). 97.8% purity; HRMS (MALDI): *m*/*z* found, 646.9437 [M + H]^+^ (calcd C_24_H_24_Br_2_ClN_2_O_5_S^+^ 646.9435).

##### (*S*)-*N*-(3-((4-Bromo-2-((3-hydroxypyrrolidin-1-yl)­methyl)­phenoxy)­methyl)­phenyl)-2-chloro-4-(methylsulfonyl)­benzamide (**23**)

The synthesis was performed according to GP6 using **11a** (52.3 mg, 0.10 mmol), (*S*)-hydroxypyrrolidine (33.3 μL, 0.40 mmol), acetic acid (34.3 μL, 0.60 mmol), and sodium cyanoborohydride (26.5 mg, 0.40 mmol) in DMF (4.0 mL). The crude was purified by preparative HPLC to yield 39.4 mg (69%) of the desired product **23** as a formate salt. ^1^H NMR (500 MHz, DMSO-*d*_6_): δ 10.76 (s, 1H), 8.20 (s, 1H), 8.13 (d, *J* = 1.6 Hz, 1H), 8.01 (dd, *J* = 8.0, 1.6 Hz, 1H), 7.90–7.87 (m, 2H), 7.60 (d, *J* = 8.2 Hz, 1H), 7.52 (d, *J* = 2.5 Hz, 1H), 7.42–7.37 (m, 2H), 7.23 (d, *J* = 7.6 Hz, 1H), 7.05 (d, *J* = 8.9 Hz, 1H), 5.16 (s, 2H), 4.24–4.21 (m, 1H), 3.77 (d, *J* = 14.2 Hz, 1H), 3.73 (d, *J* = 14.2 Hz, 1H), 3.35 (s, 3H), 2.82–2.74 (m, 2H), 2.60–2.51 (m, 2H), 2.05–1.98 (m, 1H), 1.62–1.56 (m, 1H); ^13^C NMR (125 MHz, DMSO-*d*_6_): δ 163.9, 163.8, 155.3, 143.0, 141.2, 138.7, 137.8, 132.3, 131.0, 130.7, 129.9, 129.1, 128.9, 128.1, 125.9, 123.0, 119.1, 118.3, 114.6, 112.2, 69.5, 69.3, 62.3 52.5, 52.4, 43.1, 34.2; *R*_*f*_ HPLC: 8.1 min (13 min from 10 to 95% MeCN in water (0.1% formic acid), then 7 min 95% MeCN). 99.1% purity; HRMS (MALDI): *m*/*z* found, 593.0532 [M + H]^+^ (calcd C_26_H_27_BrClN_2_O_5_S^+^ 593.0507).

##### (*R*)-*N*-(3-((4-Bromo-2-((3-hydroxypyrrolidin-1-yl)­methyl)­phenoxy)­methyl)­phenyl)-2-chloro-4-(methylsulfonyl)­benzamide (**24**)

The synthesis was performed according to GP6 using **11a** (52.3 mg, 0.10 mmol), (*R*)-hydroxypyrrolidine (33.3 μL, 0.40 mmol), acetic acid (34.3 μL, 0.60 mmol), and sodium cyanoborohydride (26.5 mg, 0.40 mmol) in DMF (4.0 mL). The crude was purified by preparative HPLC to yield 47.1 mg (79%) of the desired product **24** as a formate salt. ^1^H NMR (500 MHz, DMSO-*d*_6_): δ 10.77 (s, 1H), 8.21 (s, 1H), 8.13 (d, *J* = 1.6 Hz, 1H), 8.01 (dd, *J* = 8.0, 1.6 Hz, 1H), 7.90–7.87 (m, 2H), 7.60 (d, *J* = 8.2 Hz, 1H), 7.54 (d, *J* = 2.5 Hz, 1H), 7.42–7.38 (m, 2H), 7.23 (d, *J* = 7.6 Hz, 1H), 7.05 (d, *J* = 8.9 Hz, 1H), 5.16 (s, 2H), 4.25–4.21 (m, 1H), 3.80 (d, *J* = 14.2 Hz, 1H), 3.76 (d, *J* = 14.2 Hz, 1H), 3.35 (s, 3H), 2.85–2.77 (m, 2H), 2.63–2.59 (m, 1H), 2.56–2.53 (m, 1H), 2.06–1.99 (m, 1H), 1.64–1.57 (m, 1H); ^13^C NMR (125 MHz, DMSO-*d*_6_): δ 163.9, 163.8, 155.3, 143.0, 141.2, 138.7, 137.8, 132.4, 131.0, 130.8, 129.9, 129.0, 128.5, 128.1, 125.9, 123.0, 119.1, 118.3, 114.6, 112.2, 69.5, 69.2, 62.2, 52.4, 52.3, 43.1, 34.2; *R*_*f*_ HPLC: 7.9 min (13 min from 10 to 95% MeCN in water (0.1% formic acid), then 7 min 95% MeCN). 99.4% purity; HRMS (MALDI): *m*/*z* found, 593.0506 [M + H]^+^ (calcd C_26_H_27_BrClN_2_O_5_S^+^ 593.0507).

##### (*S*)-*N*-(3-((4-Bromo-2-((3-hydroxypiperidin-1-yl)­methyl)­phenoxy)­methyl)­phenyl)-2-chloro-4-(methylsulfonyl)­benzamide (**25**)

The synthesis was performed according to GP6 using **11a** (52.3 mg, 0.10 mmol), (*S*)-3-hydroxypiperidine (39.8 μL, 0.40 mmol), acetic acid (34.3 μL, 0.60 mmol), and sodium cyanoborohydride (26.5 mg, 0.40 mmol) in DMF (4.0 mL). The crude was purified by preparative HPLC to yield 49.9 mg (82%) of the desired product **25** as a formate salt. ^1^H NMR (500 MHz, DMSO-*d*_6_): δ 10.74 (s, 1H), 8.17 (s, 1H), 8.13 (d, *J* = 1.6 Hz, 1H), 8.01 (dd, *J* = 8.0, 1.6 Hz, 1H), 7.89–7.87 (m, 2H), 7.59 (d, *J* = 8.2 Hz, 1H), 7.48 (d, *J* = 2.5 Hz, 1H), 7.41–7.37 (m, 2H), 7.23 (d, *J* = 7.6 Hz, 1H), 7.05 (d, *J* = 8.9 Hz, 1H), 5.15 (s, 2H), 3.60 (d, *J* = 14.2 Hz, 1H), 3.55 (d, *J* = 14.2 Hz, 1H), 3.54–3.48 (m, 1H), 3.35 (s, 3H), 2.84–2.81 (m, 1H), 2.68–2.66 (m, 1H), 2.02 (t, *J* = 9.6 Hz, 1H), 1.88 (t, *J* = 9.6 Hz, 1H), 1.79–1.75 (m, 1H), 1.66–1.62 (m, 1H), 1.48–1.39 (m, 1H), 1.13–1.05 (m, 1H); ^13^C NMR (125 MHz, DMSO-*d*_6_): δ 163.7, 163.4, 155.6, 143.0, 141.2, 138.7, 137.8, 132.2, 131.0, 130.5, 129.9, 129.02, 128.95, 128.1, 125.9, 123.0, 119.1, 118.3, 114.7, 112.2, 69.5, 65.8, 60.8, 54.9, 53.0, 43.1, 32.8, 22.9; *R*_*f*_ HPLC: 8.0 min (13 min from 10 to 95% MeCN in water (0.1% formic acid), then 7 min 95% MeCN). 100.0% purity; HRMS (MALDI): *m*/*z* found, 607.0671 [M + H]^+^ (calcd C_27_H_29_BrClN_2_O_5_S^+^ 607.0664).

##### (*R*)-*N*-(3-((4-Bromo-2-((3-hydroxypiperidin-1-yl)­methyl)­phenoxy)­methyl)­phenyl)-2-chloro-4-(methylsulfonyl)­benzamide (**26**)

The synthesis was performed according to GP6 using **11a** (52.3 mg, 0.10 mmol), (*R*)-3-hydroxypiperidine hydrochloride (55.0 mg, 0.40 mmol), acetic acid (34.3 μL, 0.60 mmol), and sodium cyanoborohydride (26.5 mg, 0.40 mmol) in DMF (4.0 mL). The crude was purified by preparative HPLC to yield 45.8 mg (75%) of the desired product **26** as a formate salt. ^1^H NMR (500 MHz, DMSO-*d*_6_): δ 10.75 (s, 1H), 8.18 (s, 1H), 8.13 (d, *J* = 1.6 Hz, 1H), 8.01 (dd, *J* = 8.0, 1.6 Hz, 1H), 7.90–7.87 (m, 2H), 7.59 (d, *J* = 8.2 Hz, 1H), 7.49 (d, *J* = 2.5 Hz, 1H), 7.41–7.37 (m, 2H), 7.23 (d, *J* = 7.6 Hz, 1H), 7.05 (d, *J* = 8.9 Hz, 1H), 5.15 (s, 2H), 3.61 (d, *J* = 14.2 Hz, 1H), 3.56 (d, *J* = 14.2 Hz, 1H), 3.54–3.48 (m, 1H), 3.35 (s, 3H), 2.85–2.82 (m, 1H), 2.69–2.66 (m, 1H), 2.04 (t, *J* = 9.6 Hz, 1H), 1.90 (t, *J* = 9.6 Hz, 1H), 1.79–1.75 (m, 1H), 1.66–1.61 (m, 1H), 1.48–1.39 (m, 1H), 1.13–1.05 (m, 1H); ^13^C NMR (125 MHz, DMSO-*d*_6_): δ 163.7, 163.5, 155.6, 143.0, 141.2, 138.7, 137.8, 132.3, 131.0, 130.6, 129.9, 129.0, 128.8, 128.1, 125.9, 123.0, 119.1, 118.3, 114.7, 112.2, 69.5, 65.8, 60.7 54.9, 53.0, 43.1, 32.8, 22.9; *R*_*f*_ HPLC: 8.1 min (13 min from 10 to 95% MeCN in water (0.1% formic acid), then 7 min 95% MeCN). 100.0% purity; HRMS (MALDI): *m*/*z* found, 607.0656 [M + H]^+^ (calcd C_27_H_29_BrClN_2_O_5_S^+^ 607.0664).

##### *N*-(3-((4-Bromo-2-(((*trans*-2-hydroxycyclopentyl)­amino)­methyl)­phenoxy)­methyl)­phenyl)-2-chloro-4-(methylsulfonyl)­benzamide (**27**)

The synthesis was performed according to GP6 using **11a** (52.3 mg, 0.10 mmol), *trans*-2-aminocyclopentanol (37.3 μL, 0.40 mmol), acetic acid (34.3 μL, 0.60 mmol), and sodium cyanoborohydride (26.5 mg, 0.40 mmol) in DMF (4.0 mL). The crude was purified by preparative HPLC to yield 17.4 mg (29%) of the desired product **27** as a formate salt. ^1^H NMR (500 MHz, DMSO-*d*_6_): δ 10.78 (s, 1H), 8.23 (s, 1H), 8.12 (d, *J* = 1.6 Hz, 1H), 8.00 (dd, *J* = 8.0, 1.6 Hz, 1H), 7.89–7.86 (m, 2H), 7.64 (d, *J* = 8.2 Hz, 1H), 7.57 (d, *J* = 2.5 Hz, 1H), 7.43–7.38 (m, 2H), 7.24 (d, *J* = 7.7 Hz, 1H), 7.05 (d, *J* = 8.8 Hz, 1H), 5.16 (s, 2H), 3.89–3.82 (m, 4H), 3.34 (s, 3H), 2.86 (q, *J* = 4.7 Hz, 1H), 1.90–1.83 (m, 1H), 1.82–1.75 (m, 1H), 1.60–1.50 (m, 2H), 1.42–1.30 (m, 2H); ^13^C NMR (125 MHz, DMSO-*d*_6_): δ 164.2, 163.7, 155.4, 143.0, 141.2, 138.7, 137.6, 132.0, 131.0, 130.8, 129.9, 129.7, 129.1, 128.1, 125.9, 123.1, 119.1, 118.4, 114.3, 112.1, 75.9, 69.5, 65.5, 44.9, 43.1, 32.8, 29.1, 20.7; *R*_*f*_ HPLC: 8.2 min (13 min from 10 to 95% MeCN in water (0.1% formic acid), then 7 min 95% MeCN). 100.0% purity; HRMS (MALDI): *m*/*z* found, 607.0666 [M + H]^+^ (calcd C_27_H_29_BrClN_2_O_5_S^+^ 607.0664).

##### (*S*)-*N*-(3-((4-Bromo-2-((3-hydroxypyrrolidin-1-yl)­methyl)­phenoxy)­methyl)-2-fluorophenyl)-2-chloro-4-(methylsulfonyl)­benzamide (**28**)

The synthesis was performed according to GP6 using **11b** (130 mg, 0.240 mmol), (*S*)-hydroxypyrrolidine (79.8 μL, 0.960 mmol), acetic acid (86.5 μL, 1.44 mmol), and sodium cyanoborohydride (63.5 mg, 0.960 mmol) in DMF (7.0 mL). The crude was purified by preparative HPLC to yield 101 mg (69%) of the desired product **28**. ^1^H NMR (500 MHz, DMSO-*d*_6_): δ 10.62 (s, 1H), 8.12 (d, *J* = 1.7 Hz, 1H), 8.00 (dd, *J* = 8.0, 1.7 Hz, 1H), 7.90–7.87 (m, 2H), 7.49 (d, *J* = 2.6 Hz, 1H), 7.43 (d, *J* = 6.4 Hz, 1H), 7.40 (dd, *J* = 8.7, 2.5 Hz, 1H), 7.28 (t, *J* = 7.9 Hz, 1H), 7.10 (d, *J* = 8.8 Hz, 1H), 5.21 (s, 2H), 4.70 (br s, 1H), 4.21–4.17 (m, 1H), 3.61 (d, *J* = 14.4 Hz, 1H), 3.56 (d, *J* = 14.4 Hz, 1H), 3.35 (s, 3H), 2.68–2.61 (m, 2H), 2.44–2.41 (m, 1H), 2.37 (dd, *J* = 9.6, 3.5 Hz, 1H), 2.03–1.96 (m, 1H), 1.57–1.52 (m, 1H); ^13^C NMR (125 MHz, DMSO-*d*_6_): δ 164.2, 155.1, 152.5 (d, *J* = 248.0 Hz), 143.0, 140.9, 131.9, 131.0, 130.2, 130.0, 128.0, 126.5 (d, *J* = 2.2 Hz), 125.8, 125.2 (d, *J* = 11.8 Hz), 125.0, 124.4 (d, *J* = 13.0 Hz), 124.2 (d, *J* = 3.9 Hz), 114.6, 112.5, 69.4, 64.1, 62.6, 52.6, 52.4, 43.1, 34.4; ^19^F NMR (471 MHz, DMSO-*d*_6_): δ −128.0; *R*_*f*_ HPLC: 7.9 min (13 min from 10 to 95% MeCN in water (0.1% formic acid), then 7 min 95% MeCN). 99.3% purity; HRMS (MALDI): *m*/*z* found, 611.0396 [M + H]^+^ (calcd C_26_H_26_BrClFN_2_O_5_S^+^ 611.0413).

##### (*S*)-*N*-(3-((4-Bromo-2-((3-hydroxypyrrolidin-1-yl)­methyl)­phenoxy)­methyl)-5-fluorophenyl)-2-chloro-4-(methylsulfonyl)­benzamide (**29**)

The synthesis was performed according to GP6 using **11d** (100 mg, 0.185 mmol), (*S*)-hydroxypyrrolidine (61.5 μL, 0.740 mmol), acetic acid (63.5 μL, 1.11 mmol), and sodium cyanoborohydride (48.9 mg, 0.740 mmol) in DMF (6.0 mL). The crude was purified by preparative HPLC to yield 65.7 mg (58%) of the desired product **29** as a formate salt. ^1^H NMR (500 MHz, DMSO-*d*_6_): δ 10.96 (s, 1H), 8.18 (s, 1H), 8.14 (d, *J* = 1.7 Hz, 1H), 8.01 (dd, *J* = 8.0, 1.7 Hz, 1H), 7.90 (d, *J* = 8.0 Hz, 1H), 7.60 (s, 1H), 7.56 (d, *J* = 10.8 Hz, 1H), 7.51 (s, 1H), 7.40 (dd, *J* = 8.7, 2.5 Hz, 1H), 7.09 (d, *J* = 9.2 Hz, 1H), 7.02 (d, *J* = 8.8 Hz, 1H), 5.17 (s, 2H), 4.23–4.19 (m, 1H), 3.74 (d, *J* = 14.3 Hz, 1H), 3.69 (d, *J* = 14.3 Hz, 1H), 3.35 (s, 3H), 2.79–2.69 (m, 2H), 2.56–2.50 (m, 1H), 2.47–2.44 (m, 1H), 2.04–1.97 (m, 1H), 1.59–1.55 (m, 1H); ^13^C NMR (125 MHz, DMSO-*d*_6_): δ 164.0, 163.5, 162.1 (d, *J* = 240.6 Hz) 155.0, 143.2, 140.8, 140.4 (d, *J* = 8.9 Hz), 140.2 (d, *J* = 11.4 Hz), 132.2, 130.9, 130.6, 129.9, 129.4, 128.1, 114.5, 113.7 (d, *J* = 2.0 Hz), 112.3, 109.3 (d, *J* = 22.3 Hz), 105.7 (d, *J* = 26.2 Hz), 69.3, 68.7, 62.4, 52.7, 52.4, 43.1, 34.3; ^19^F NMR (471 MHz, DMSO-*d*_6_): δ −111.7; *R*_*f*_ HPLC: 8.2 min (13 min from 10 to 95% MeCN in water (0.1% formic acid), then 7 min 95% MeCN). 98.2% purity; HRMS (MALDI): *m*/*z* found, 611.0413 [M + H]^+^ (calcd C_26_H_26_BrClFN_2_O_5_S^+^ 611.0413).

##### (*S*)-*N*-(3-((4-Bromo-2-((3-hydroxypyrrolidin-1-yl)­methyl)­phenoxy)­methyl)-4-fluorophenyl)-2-chloro-4-(methylsulfonyl)­benzamide (**30**)

The synthesis was performed according to GP6 using **11e** (100 mg, 0.185 mmol), (*S*)-hydroxypyrrolidine (61.5 μL, 0.740 mmol), acetic acid (63.5 μL, 1.11 mmol), and sodium cyanoborohydride (48.9 mg, 0.740 mmol) in DMF (6.0 mL). The crude was purified by preparative HPLC to yield 70.5 mg (62%) of the desired product **30** as a formate salt. ^1^H NMR (500 MHz, DMSO-*d*_6_): δ 10.78 (s, 1H), 8.16 (s, 1H), 8.12 (d, *J* = 1.7 Hz, 1H), 8.00 (dd, *J* = 8.0, 1.7 Hz, 1H), 7.95 (dd, *J* = 6.6, 2.7 Hz, 1H), 7.88 (d, *J* = 8.0 Hz, 1H), 7.68–7.64 (m, 1H), 7.49 (d, *J* = 2.6 Hz, 1H), 7.40 (dd, *J* = 8.7, 2.6 Hz, 1H), 7.28 (t, *J* = 9.2 Hz, 1H), 7.08 (d, *J* = 8.8 Hz, 1H), 5.18 (s, 2H), 4.20–4.15 (m, 1H), 3.65 (d, *J* = 14.5 Hz, 1H), 3.61 (d, *J* = 14.5 Hz, 1H), 3.34 (s, 3H), 2.70–2.62 (m, 2H), 2.47–2.42 (m, 1H), 2.40 (dd, *J* = 9.8, 3.5 Hz, 1H), 2.02–1.95 (m, 1H), 1.56–1.50 (m, 1H); ^13^C NMR (125 MHz, DMSO-*d*_6_): δ 163.6, 163.3, 156.3 (d, *J* = 241.9 Hz), 155.0, 143.0, 141.0, 134.9 (d, *J* = 2.5 Hz), 131.9, 131.0, 130.3, 130.0, 129.9, 128.1, 125.9, 124.1 (d, *J* = 15.5 Hz), 121.2 (d, *J* = 8.0 Hz), 121.0 (d, *J* = 3.5 Hz), 115.7 (d, *J* = 22.2 Hz), 114.6, 112.5, 69.4, 64.0, 62.5, 52.44, 52.37, 43.1, 34.4; ^19^F NMR (471 MHz, DMSO-*d*_6_): δ −123.1; *R*_*f*_ HPLC: 8.1 min (13 min from 10 to 95% MeCN in water (0.1% formic acid), then 7 min 95% MeCN). 96.4% purity; HRMS (MALDI): *m*/*z* found, 611.0413 [M + H]^+^ (calcd C_26_H_26_BrClFN_2_O_5_S^+^ 611.0413).

##### (*S*)-*N*-(3-((4-Bromo-5-fluoro-2-((3-hydroxypyrrolidin-1-yl)­methyl)­phenoxy)­methyl)­phenyl)-2-chloro-4-(methylsulfonyl)­benzamide (**31**)

The synthesis was performed according to GP6 using **11n** (115 mg, 0.213 mmol), (*S*)-hydroxypyrrolidine (70.8 μL, 0.852 mmol), acetic acid (73.1 μL, 1.28 mmol), and sodium cyanoborohydride (56.4 mg, 0.852 mmol) in DMF (7.0 mL). The crude was purified by preparative HPLC to yield 68.3 mg (52%) of the desired product **31** as a formate salt. ^1^H NMR (500 MHz, DMSO-*d*_6_): δ 10.77 (s, 1H), 8.19 (s, 1H), 8.13 (d, *J* = 1.7 Hz, 1H), 8.01 (dd, *J* = 8.0, 1.7 Hz, 1H), 7.90–7.88 (m, 2H), 7.61 (d, *J* = 8.4 Hz, 1H), 7.41 (t, *J* = 7.9 Hz, 1H), 7.25 (d, *J* = 7.8 Hz, 1H), 7.22 (d, *J* = 11.1 Hz, 1H), 5.19 (s, 2H), 4.23–4.19 (m, 1H), 3.73 (d, *J* = 14.1 Hz, 1H), 3.69 (d, *J* = 14.1 Hz, 1H), 3.35 (s, 3H), 2.78 (dd, *J* = 10.0, 6.0 Hz, 1H), 2.76–2.72 (m, 1H), 2.57–2.50 (m, 1H), 2.49 (dd, *J* = 10.0, 3.3 Hz, 1H), 2.05–1.98 (m, 1H), 1.61–1.55 (m, 1H); ^13^C NMR (125 MHz, DMSO-*d*_6_): δ 163.8, 163.6, 157.8 (d, *J* = 241.7 Hz), 156.6 (d, *J* = 9.1 Hz), 143.0, 141.2, 138.7, 137.3, 133.4, 131.0, 129.9, 129.1, 128.1, 125.9, 124.6 (d, *J* = 3.1 Hz), 123.1, 119.2, 118.4, 102.2 (d, *J* = 26.1 Hz), 97.7 (d, *J* = 20.9 Hz), 69.9, 69.3, 62.2, 52.3, 52.0, 43.1, 34.2; ^19^F NMR (471 MHz, DMSO-*d*_6_): δ −108.2; *R*_*f*_ HPLC: 8.2 min (13 min from 10 to 95% MeCN in water (0.1% formic acid), then 7 min 95% MeCN). 99.4% purity; HRMS (MALDI): *m*/*z* found, 611.0409 [M + H]^+^ (calcd C_26_H_26_BrClFN_2_O_5_S^+^ 611.0413).

##### (*S*)-*N*-(3-((4-Bromo-2-((3-hydroxypyrrolidin-1-yl)­methyl)­phenoxy)­methyl)­phenyl)-4-(methylsulfonyl)­benzamide (**32**)

The synthesis was performed according to GP6 using **11m** (40.0 mg, 0.0819 mmol), (*S*)-hydroxypyrrolidine (27.2 μL, 0.328 mmol), acetic acid (28.1 μL, 0.491 mmol), and sodium cyanoborohydride (21.7 mg, 0.328 mmol) in DMF (3.0 mL). The crude was purified by preparative HPLC to yield 28.9 mg (63%) of the desired product **32** as a formate salt. ^1^H NMR (500 MHz, DMSO-*d*_6_): δ 10.56 (s, 1H), 8.20 (s, 1H), 8.17 (d, *J* = 8.5 Hz, 2H), 8.09 (d, *J* = 8.5 Hz, 2H), 7.95 (s, 1H), 7.70 (d, *J* = 8.1 Hz, 1H), 7.52 (d, *J* = 2.6 Hz, 1H), 7.41–7.38 (m, 2H), 7.23 (d, *J* = 7.7 Hz, 1H), 7.05 (d, *J* = 8.8 Hz, 1H), 5.16 (s, 2H), 4.24–4.20 (m, 1H), 3.76 (d, *J* = 14.3 Hz, 1H), 3.72 (d, *J* = 14.3 Hz, 1H), 3.29 (s, 3H), 2.80 (dd, *J* = 10.0, 6.0 Hz, 1H), 2.77–2.73 (m, 1H), 2.59–2.54 (m, 1H), 2.51–2.48 (m, 1H), 2.05–1.98 (m, 1H), 1.62–1.55 (m, 1H); ^13^C NMR (125 MHz, DMSO-*d*_6_): δ 164.4, 163.7, 155.3, 143.2, 139.4, 139.0, 137.5, 132.2, 130.6, 129.0, 128.9, 128.8, 128.7, 127.1, 123.0, 119.9, 119.2, 114.6, 112.2, 69.6, 69.3, 62.3, 52.5, 52.4, 43.3, 34.3; *R*_*f*_ HPLC: 7.9 min (13 min from 10 to 95% MeCN in water (0.1% formic acid), then 7 min 95% MeCN). 99.0% purity; HRMS (MALDI): *m*/*z* found, 559.0880 [M + H]^+^ (calcd C_26_H_28_BrN_2_O_5_S^+^ 559.0897).

##### (*S*)-*N*-(3-((4-Bromo-2-((3-hydroxypyrrolidin-1-yl)­methyl)-5-methoxyphenoxy)­methyl)­phenyl)-2-chloro-4-(methylsulfonyl)­benzamide (**33**)

The synthesis was performed according to GP6 using **11q** (140 mg, 0.253 mmol), (*S*)-hydroxypyrrolidine (84.1 μL, 1.01 mmol), acetic acid (86.8 μL, 1.52 mmol), and sodium cyanoborohydride (66.9 mg, 1.01 mmol) in DMF (6.0 mL). The crude was purified by preparative HPLC to yield 72.6 mg (46%) of the desired product **33** as a formate salt. ^1^H NMR (500 MHz, DMSO-*d*_6_): δ 10.75 (s, 1H), 8.19 (s, 1H), 8.13 (d, *J* = 1.7 Hz, 1H), 8.00 (dd, *J* = 8.0, 1.7 Hz, 1H), 7.94 (s, 1H), 7.88 (d, *J* = 8.0 Hz, 1H), 7.58 (d, *J* = 8.1 Hz, 1H), 7.44 (s, 1H), 7.40 (t, *J* = 7.9 Hz, 1H), 7.27 (d, *J* = 7.7 Hz, 1H), 6.88 (s, 1H), 5.21 (s, 2H), 4.20–4.16 (m, 1H), 3.86 (s, 3H), 3.63 (d, *J* = 13.7 Hz, 1H), 3.58 (d, *J* = 13.7 Hz, 1H), 3.34 (s, 3H), 2.70 (dd, *J* = 9.9, 6.2 Hz, 1H), 2.65 (q, *J* = 7.9 Hz, 1H), 2.48–2.44 (m, 1H), 2.39 (dd, *J* = 9.8, 3.6 Hz, 1H), 2.01–1.95 (m, 1H), 1.56–1.50 (m, 1H); ^13^C NMR (125 MHz, DMSO-*d*_6_): δ 163.74, 163.68, 156.5, 155.0, 143.0, 141.2, 138.7, 137.8, 133.2, 130.9, 129.9, 129.0, 128.1, 125.9, 123.2, 120.4, 119.1, 118.6, 100.7, 99.1, 69.7, 69.3, 62.3, 56.4, 52.2, 52.0, 43.1, 34.3; *R*_*f*_ HPLC: 8.1 min (13 min from 10 to 95% MeCN in water (0.1% formic acid), then 7 min 95% MeCN). 100.0% purity; HRMS (MALDI): *m*/*z* found, 623.0611 [M + H]^+^ (calcd C_27_H_28_BrClN_2_O_6_S^+^ 623.0613).

##### (*S*)-1-(5-Bromo-2-((3-(2-chloro-4-(methylsulfonyl)­benzamido)­benzyl)­oxy)­benzyl)­pyrrolidine-3-carboxamide (**34**)

The synthesis was performed according to GP6 using **11a** (86.8 mg, 0.166 mmol), (*S*)- pyrrolidine-3-carboxamide hydrochloride (100 mg, 0.664 mmol), acetic acid (57.0 μL, 0.996 mmol), and sodium cyanoborohydride (43.9 mg, 0.664 mmol) in DMF (6.0 mL). The crude was purified by preparative HPLC to yield 46.5 mg (44%) of the desired product **34**. ^1^H NMR (500 MHz, DMSO-*d*_6_): δ 10.73 (s, 1H), 8.12 (d, *J* = 1.7 Hz, 1H), 8.00 (dd, *J* = 8.0, 1.7 Hz, 1H), 7.88 (d, *J* = 8.0 Hz, 1H), 7.85 (s, 1H), 7.61 (d, *J* = 8.2 Hz, 1H), 7.46 (d, *J* = 2.6 Hz, 1H), 7.41–7.36 (m, 2H), 7.24–7.21 (m, 2H), 7.03 (d, *J* = 7.5 Hz, 1H), 6.74 (br s, 1H), 5.15 (s, 2H), 3.64 (s, 2H), 3.34 (s, 3H), 2.83–2.77 (m, 2H), 2.66–2.62 (m, 1H), 2.52–2.50 (m, 1H), 2.50–2.45 (m, 1H), 1.91–1.87 (m, 2H); ^13^C NMR (125 MHz, DMSO-*d*_6_): δ 175.6, 163.7, 155.2, 143.0, 141.2, 138.7, 137.8, 131.8, 130.9, 130.3, 130.1, 129.9, 129.0, 128.0, 125.9, 122.9, 119.0, 118.2, 114.5, 112.2, 69.4, 57.0, 53.6, 52.3, 43.1, 42.3, 27.5; *R*_*f*_ HPLC: 8.2 min (13 min from 10 to 95% MeCN in water (0.1% formic acid), then 7 min 95% MeCN). 98.7% purity; HRMS (MALDI): *m*/*z* found, 620.0626 [M + H]^+^ (calcd C_27_H_28_BrClN_3_O_5_S^+^ 620.0616).

##### (*S*)-*N*-(3-((4-Bromo-2-((3-hydroxypyrrolidin-1-yl)­methyl)­phenoxy)­methyl)-2,4-difluorophenyl)-2-chloro-4-(methylsulfonyl)­benzamide (**35**)

The synthesis was performed according to GP6 using **11h** (90.0 mg, 0.161 mmol), (*S*)-hydroxypyrrolidine (53.5 μL, 0.644 mmol), acetic acid (55.4 μL, 0.966 mmol), and sodium cyanoborohydride (42.6 mg, 0.664 mmol) in DMF (6.0 mL). The crude was purified by preparative HPLC to yield 16.8 mg (17%) of the desired product **35** as a formate salt. ^1^H NMR (500 MHz, DMSO-*d*_6_): δ 10.63 (s, 1H), 8.18 (s, 1H), 8.12 (d, *J* = 1.7 Hz, 1H), 8.00 (dd, *J* = 8.0, 1.7 Hz, 1H), 7.93–7.87 (m, 2H), 7.48 (d, *J* = 2.6 Hz, 1H), 7.43 (dd, *J* = 8.7, 2.6 Hz, 1H), 7.24 (t, *J* = 8.9 Hz, 1H), 7.17 (d, *J* = 8.8 Hz, 1H), 5.18 (s, 2H), 4.18–4.14 (m, 1H), 3.53 (d, *J* = 14.5 Hz, 1H), 3.48 (d, *J* = 14.5 Hz, 1H), 3.34 (s, 3H), 2.62 (dd, *J* = 9.8, 6.2 Hz, 1H), 2.58 (q, *J* = 7.8 Hz, 1H), 2.39–2.35 (m, 1H), 2.33 (dd, *J* = 10.0, 3.5 Hz, 1H), 2.00–1.93 (m, 1H), 1.55–1.49 (m, 1H); ^13^C NMR (125 MHz, DMSO-*d*_6_): δ 164.3, 163.5, 158.2 (dd, *J* = 246.4, 6.4 Hz) 155.1, 153.7 (dd, *J* = 251.2, 8.0 Hz), 143.1, 140.7, 131.9, 131.0, 130.3, 130.0, 128.0, 127.0 (dd, *J* = 10.4, 3.0 Hz), 125.8, 121.7 (dd, *J* = 13.2, 3.6 Hz), 115.0, 113.0, 112.6 (dd, *J* = 20.2, 17.3 Hz), 111.3 (dd, *J* = 21.9, 3.0 Hz), 69.4, 62.4, 58.8, 52.3, 52.2, 43.1, 34.4; ^19^F NMR (471 MHz, DMSO-*d*_6_): δ −117.8, −122.2; *R*_*f*_ HPLC: 8.0 min (13 min from 10 to 95% MeCN in water (0.1% formic acid), then 7 min 95% MeCN). 99.4% purity; HRMS (MALDI): *m*/*z* found, 629.0311 [M + H]^+^ (calcd C_26_H_25_BrClF_2_N_2_O_5_S^+^ 629.0319).

##### (*S*)-*N*-(3-((4-Bromo-5-fluoro-2-((3-hydroxypyrrolidin-1-yl)­methyl)­phenoxy)­methyl)-2,4-difluorophenyl)-2-chloro-4-(methylsulfonyl)­benzamide (**36**)

The synthesis was performed according to GP6 using **11p** (100 mg, 0.173 mmol), (*S*)-hydroxypyrrolidine (57.5 μL, 0.692 mmol), acetic acid (59.5 μL, 1.04 mmol), and sodium cyanoborohydride (45.8 mg, 0.692 mmol) in DMF (6.0 mL). The crude was purified by preparative HPLC to yield 50.2 mg (46%) of the desired product **36** as a formate salt. ^1^H NMR (500 MHz, DMSO-*d*_6_): δ 10.64 (s, 1H), 8.16 (s, 1H), 8.12 (d, *J* = 1.7 Hz, 1H), 8.01 (dd, *J* = 8.0, 1.7 Hz, 1H), 7.93–7.89 (m, 1H), 7.88 (d, *J* = 8.0 Hz, 1H), 7.58 (d, *J* = 8.3 Hz, 1H), 7.36 (d, *J* = 10.9 Hz, 1H), 7.26 (t, *J* = 8.9 Hz, 1H), 5.22 (s, 2H), 4.18–4.14 (m, 1H), 3.53 (d, *J* = 13.9 Hz, 1H), 3.48 (d, *J* = 13.9 Hz, 1H), 3.34 (s, 3H), 2.65–2.58 (m, 2H), 2.42–2.33 (m, 2H), 1.99–1.92 (m, 1H), 1.55–1.49 (m, 1H); ^13^C NMR (125 MHz, DMSO-*d*_6_): δ 164.3, 163.3, 158.2 (dd, *J* = 247.1, 6.6 Hz), 157.6 (d, *J* = 241.2 Hz), 156.2 (d, *J* = 9.0 Hz), 153.7 (dd, *J* = 251.6, 7.9 Hz), 143.1, 140.7, 133.2, 131.0, 130.0, 128.0, 127.2 (d, *J* = 9.4 Hz), 125.8, 125.3, 121.7 (dd, *J* = 12.8, 3.5 Hz), 112.2 (dd, *J* = 20.2, 17.6 Hz), 111.4 (dd, *J* = 22.4, 3.1 Hz), 102.4 (d, *J* = 26.0 Hz), 98.4 (d, *J* = 20.9 Hz), 69.3, 62.2, 59.1, 52.1, 51.6, 43.1, 34.3; ^19^F NMR (471 MHz, DMSO-*d*_6_): δ −108.1, −117.6, −122.1; *R*_*f*_ HPLC: 8.1 min (13 min from 10 to 95% MeCN in water (0.1% formic acid), then 7 min 95% MeCN). 98.3% purity; HRMS (MALDI): *m*/*z* found, 647.0218 [M + H]^+^ (calcd C_26_H_24_BrClF_3_N_2_O_5_S^+^ 647.0224).

##### (*S*)-*N*-(3-((4-Bromo-5-fluoro-2-((3-hydroxypyrrolidin-1-yl)­methyl)­phenoxy)­methyl)-5-fluorophenyl)-2-chloro-4-(methylsulfonyl)­benzamide (**37**)

The synthesis was performed according to GP6 using **11o** (200 mg, 0.173 mmol), (*S*)-hydroxypyrrolidine (119 μL, 1.43 mmol), acetic acid (123 μL, 2.15 mmol), and sodium triacetoxyborohydride (379 mg, 1.43 mmol) in DMF (6.0 mL). The crude was purified by preparative HPLC, followed by flash chromatography eluting with DCM/MeOH (95:5 to 90:10) to yield 60.0 mg (27%) of the desired product **37**. ^1^H NMR (500 MHz, DMSO-*d*_6_): δ 10.95 (s, 1H), 8.14 (d, *J* = 1.7 Hz, 1H), 8.02 (dd, *J* = 8.0, 1.7 Hz, 1H), 7.90 (d, *J* = 8.0 Hz, 1H), 7.61–7.54 (m, 3H), 7.18 (d, *J* = 11.1 Hz, 1H), 7.09 (d, *J* = 8.9 Hz, 1H), 5.19 (s, 2H), 4.69 (br s, 1H), 4.21–4.16 (m, 1H), 3.63 (d, *J* = 14.1 Hz, 1H), 3.58 (d, *J* = 14.1 Hz, 1H), 3.35 (s, 3H), 2.69 (dd, *J* = 9.7, 6.2 Hz, 1H), 2.63 (q, *J* = 7.9 Hz, 1H), 2.45–2.41 (m, 1H), 2.37 (dd, *J* = 9.6, 3.6 Hz, 1H), 2.02–1.95 (m, 1H), 1.57–1.51 (m, 1H); ^13^C NMR (125 MHz, DMSO-*d*_6_): δ 164.0, 162.1 (d, *J* = 240.6 Hz), 157.5 (d, *J* = 241.3 Hz), 156.2 (d, *J* = 9.0 Hz), 143.2, 140.8, 140.2 (d, *J* = 11.3 Hz), 139.9 (d, *J* = 9.0 Hz), 133.0, 131.0, 129.9, 128.1, 126.0, 125.7 (d, *J* = 3.0 Hz), 113.9 (d, *J* = 2.0 Hz), 109.4 (d, *J* = 22.3 Hz), 105.8 (d, *J* = 26.1 Hz), 102.1 (d, *J* = 26.1 Hz), 97.8 (d, *J* = 20.8 Hz), 102.4 (d, *J* = 26.0 Hz), 98.4 (d, *J* = 20.9 Hz), 69.4, 69.1, 62.5, 52.34, 52.30, 43.1, 34.4; ^19^F NMR (471 MHz, DMSO-*d*_6_): δ −108.3, −111.6; *R*_*f*_ HPLC: 8.3 min (13 min from 10 to 95% MeCN in water (0.1% formic acid), then 7 min 95% MeCN). 97.9% purity; HRMS (MALDI): *m*/*z* found, 651.0133 [M + Na]^+^ (calcd C_26_H_24_BrClF_2_N_2_NaO_5_S^+^ 651.0138).

##### (*S*)-*N*-(3-((4-Bromo-5-fluoro-2-((3-hydroxypyrrolidin-1-yl)­methyl)­phenoxy)­methyl)-4,5-difluorophenyl)-2-chloro-4-(methylsulfonyl)­benzamide (**38**)

The synthesis was performed according to GP6 using **11i** (90.0 mg, 0.156 mmol), (*S*)-hydroxypyrrolidine (51.9 μL, 0.624 mmol), acetic acid (53.6 μL, 0.936 mmol), and sodium triacetoxyborohydride (165 mg, 0.624 mmol) in DMF (6.0 mL). The crude was purified by preparative HPLC to yield 59.0 mg (58%) of the desired product **38** as a formate salt. ^1^H NMR (500 MHz, DMSO-*d*_6_): δ 10.97 (s, 1H), 8.16 (s, 1H), 8.13 (d, *J* = 1.7 Hz, 1H), 8.01 (dd, *J* = 8.0, 1.7 Hz, 1H), 7.90 (d, *J* = 8.0 Hz, 1H), 7.80 (ddd, *J* = 12.3, 7.0, 2.5 Hz, 1H), 7.68–7.66 (m, 1H), 7.60 (d, *J* = 8.4 Hz, 1H), 7.30 (t, *J* = 11.0 Hz, 1H), 5.27 (s, 2H), 4.19–4.15 (m, 1H), 3.64 (d, *J* = 13.9 Hz, 1H), 3.60 (d, *J* = 13.9 Hz, 1H), 3.34 (s, 3H), 2.70 (dd, *J* = 9.9, 6.1 Hz, 1H), 2.66 (q, *J* = 7.8 Hz, 1H), 2.50–2.43 (m, 1H), 2.40 (dd, *J* = 9.8, 3.4 Hz, 1H), 2.01–1.94 (m, 1H), 1.56–1.50 (m, 1H); ^13^C NMR (125 MHz, DMSO-*d*_6_): δ 163.9, 163.3, 157.7 (d, *J* = 241.8 Hz), 157.6 (d, *J* = 241.2 Hz), 156.1 (d, *J* = 9.1 Hz), 149.1 (dd, *J* = 242.6, 12.8 Hz), 144.0 (dd, *J* = 244.1, 13.2 Hz), 143.2, 140.6, 135.0 (dd, *J* = 9.5, 2.8 Hz), 133.3, 131.0, 130.0, 128.1, 126.1 (d, *J* = 11.9 Hz), 126.0, 125.1 (d, *J* = 3.0 Hz), 115.6, 108.5 (d, *J* = 21.5 Hz), 102.3 (d, *J* = 26.2 Hz), 98.3 (d, *J* = 20.9 Hz), 69.3, 64.2, 62.2, 52.2, 51.9, 43.1, 34.3; ^19^F NMR (471 MHz, DMSO-*d*_6_): δ −108.1, −136.9, −147.8; *R*_*f*_ HPLC: 8.5 min (13 min from 10 to 95% MeCN in water (0.1% formic acid), then 7 min 95% MeCN). 98.9% purity; HRMS (MALDI): *m*/*z* found, 669.0041 [M + Na]^+^ (calcd C_26_H_23_BrClF_3_N_2_NaO_5_S^+^ 669.0044).

##### (*S*)-1-(5-Bromo-2-((3-(2-chloro-4-(methylsulfonyl)­benzamido)-5-fluorobenzyl)­oxy)-4-fluorobenzyl)­pyrrolidine-3-carboxamide (**39**)

The synthesis was performed according to GP6 using **11o** (115 mg, 0.206 mmol), (*S*)- pyrrolidine-3-carboxamide hydrochloride (124 mg, 0.824 mmol), acetic acid (70.8 μL, 1.24 mmol), and sodium triacetoxyborohydride (218 mg, 0.824 mmol) in DMF (6.0 mL). The crude was purified by preparative HPLC to yield 59.0 mg (44%) of the desired product **39**. ^1^H NMR (500 MHz, DMSO-*d*_6_): δ 10.95 (s, 1H), 8.13 (s, 1H), 8.01 (d, *J* = 6.9 Hz, 1H), 7.90 (d, *J* = 7.2 Hz, 1H), 7.59–7.57 (m, 3H), 7.23 (br s, 1H), 7.19 (d, *J* = 10.7 Hz, 1H), 7.10 (d, *J* = 7.6 Hz, 1H), 6.73 (br s, 1H), 5.20 (s, 2H), 3.61 (s, 2H), 3.35 (s, 3H), 2.83–2.75 (m, 2H), 2.65–2.60 (m, 1H), 2.51–2.43 (m, 2H), 1.92–1.85 (m, 2H); ^13^C NMR (125 MHz, DMSO-*d*_6_): δ 175.7, 164.0, 162.1 (d, *J* = 240.8 Hz), 157.6 (d, *J* = 241.4 Hz), 156.2 (d, *J* = 9.0 Hz), 143.2, 140.8, 140.2 (d, *J* = 11.3 Hz), 139.9 (d, *J* = 8.9 Hz), 133.1, 131.0, 130.0, 128.1, 126.0, 125.7 (d, *J* = 2.5 Hz), 113.8, 109.4 (d, *J* = 22.3 Hz), 105.8 (d, *J* = 26.3 Hz), 102.1 (d, *J* = 26.2 Hz), 97.8 (d, *J* = 20.8 Hz), 69.1, 56.9, 53.5, 51.9, 43.1, 42.3, 27.6; ^19^F NMR (471 MHz, DMSO-*d*_6_): δ −108.2, −111.5; *R*_*f*_ HPLC: 8.2 min (13 min from 10 to 95% MeCN in water (0.1% formic acid), then 7 min 95% MeCN). 98.9% purity; HRMS (MALDI): *m*/*z* found, 678.0242 [M + Na]^+^ (calcd C_27_H_25_BrClF_2_N_3_NaO_5_S^+^ 678.0247).

##### (*S*)-1-(5-Bromo-2-((3-(2-chloro-4-(methylsulfonyl)­benzamido)-2,6-difluorobenzyl)­oxy)-4-fluorobenzyl)­pyrrolidine-3-carboxamide (**40**)

The synthesis was performed according to GP6 using **11p** (61 mg, 0.106 mmol), (*S*)- pyrrolidine-3-carboxamide hydrochloride (63.9 mg, 0.424 mmol), acetic acid (36.4 μL, 0.636 mmol), and sodium triacetoxyborohydride (112 mg, 0.424 mmol) in DMF (4.0 mL). The crude was purified by preparative HPLC to yield 29.8 mg (42%) of the desired product **40** as a formate salt. ^1^H NMR (500 MHz, DMSO-*d*_6_): δ 10.65 (s, 1H), 8.16 (s, 1H), 8.12 (d, *J* = 1.7 Hz, 1H), 8.01 (dd, *J* = 8.0, 1.7 Hz, 1H), 7.94–7.89 (m, 1H), 7.89 (d, *J* = 8.0 Hz, 1H), 7.56 (d, *J* = 8.4 Hz, 1H), 7.36 (d *J* = 11.0 Hz, 1H), 7.25 (t, *J* = 9.0 Hz, 1H), 7.22 (br s, 1H), 6.75 (br s, 1H), 5.22 (s, 2H), 3.47 (s, 2H), 3.35 (s, 3H), 2.80–2.69 (m, 2H), 2.57–2.53 (m, 1H), 2.43–2.40 (m, 1H), 2.37 (q, *J* = 8.4 Hz, 1H), 1.87–1.82 (m, 2H); ^13^C NMR (125 MHz, DMSO-*d*_6_): δ 175.6, 164.3, 158.2 (dd, *J* = 247.1, 6.6 Hz), 157.6 (d, *J* = 241.8 Hz), 156.2 (d, *J* = 9.0 Hz), 153.7 (dd, *J* = 251.6, 7.8 Hz), 143.1, 140.7, 133.1 131.0, 130.1, 128.1, 127.2 (dd, *J* = 10.0, 2.2 Hz), 125.9, 125.7 (d, *J* = 2.7 Hz), 121.7 (dd, *J* = 12.7, 3.2 Hz), 112.2 (dd, *J* = 20.0, 17.8 Hz), 111.4 (dd, *J* = 22.5, 2.6 Hz), 102.5 (d, *J* = 26.0 Hz), 98.5 (d, *J* = 20.9 Hz), 59.1, 56.8, 53.3, 51.3, 43.1, 42.3, 27.5; ^19^F NMR (471 MHz, DMSO-*d*_6_): δ −107.9, −117.6, −122.1; *R*_*f*_ HPLC: 7.9 min (13 min from 10 to 95% MeCN in water (0.1% formic acid), then 7 min 95% MeCN). 95.1% purity; HRMS (MALDI): *m*/*z* found, 674.0332 [M + H]^+^ (calcd C_27_H_25_BrClF_3_N_3_O_5_S^+^ 674.0333).

##### (*S*)-1-(5-Bromo-2-((3-(2-chloro-4-(methylsulfonyl)­benzamido)-2,5,6-trifluorobenzyl)­oxy)-4-fluorobenzyl)­pyrrolidine-3-carboxamide (**41**)

The synthesis was performed according to GP6 using **11k** (37.8 mg, 0.0636 mmol), (*S*)- pyrrolidine-3-carboxamide hydrochloride (38.3 mg, 0.254 mmol), acetic acid (21.9 μL, 0.382 mmol), and sodium triacetoxyborohydride (67.4 mg, 0.254 mmol) in DMF (4.0 mL). The crude was purified by preparative HPLC to yield 18.0 mg (41%) of the desired product **41** as a formate salt. ^1^H NMR (500 MHz, DMSO-*d*_6_): δ 10.85 (s, 1H), 8.15 (s, 1H), 8.14–8.10 (m, 1H), 8.12 (d, *J* = 1.7 Hz, 1H), 8.01 (dd, *J* = 8.0, 1.7 Hz, 1H), 7.89 (d, *J* = 8.0 Hz, 1H), 7.58 (d, *J* = 8.3 Hz, 1H), 7.38 (d *J* = 10.9 Hz, 1H), 7.22 (br s, 1H), 6.75 (br s, 1H), 5.28 (s, 2H), 3.51 (s, 2H), 3.34 (s, 3H), 2.81–2.71 (m, 2H), 2.60–2.55 (m, 1H), 2.45 (dd, *J* = 8.4, 6.9 Hz, 1H), 2.40 (q, *J* = 8.6 Hz, 1H), 1.86 (q, *J* = 7.1 Hz, 2H); ^13^C NMR (125 MHz, DMSO-*d*_6_): δ 175.5, 164.5, 163.3, 157.6 (d, *J* = 242.1 Hz), 156.1 (d, *J* = 9.0 Hz), 149.8–149.7 (m), 147.8–147.7 (m), 146.4–146.2 (m), 144.5–144.2 (m), 143.2, 140.4, 133.3 131.0, 130.1, 128.0, 125.8, 125.5 (d, *J* = 3.0 Hz), 122.0–121.7 (m), 113.9 (dd, *J* = 19.5, 16.4 Hz), 113.5 (d, *J* = 21.9 Hz), 102.6 (d, *J* = 26.0 Hz), 98.7 (d, *J* = 20.9 Hz), 59.1, 56.7, 53.3, 51.3, 43.1, 42.2, 27.5; ^19^F NMR (471 MHz, DMSO-*d*_6_): δ −107.7, −127.6, −140.9, −141.7; *R*_*f*_ HPLC: 8.1 min (13 min from 10 to 95% MeCN in water (0.1% formic acid), then 7 min 95% MeCN). 99.6% purity; HRMS (MALDI): *m*/*z* found, 692.0233 [M + H]^+^ (calcd C_27_H_24_BrClF_4_N_3_O_5_S^+^ 692.0239).

##### (*S*)-*N*-(3-((4-Bromo-5-fluoro-2-((3-hydroxypyrrolidin-1-yl)­methyl)­phenoxy)­methyl)-2,4,5-trifluorophenyl)-2-chloro-4-(methylsulfonyl)­benzamide (**42**)

The synthesis was performed according to GP6 using **11k** (37.8 mg, 0.0636 mmol), (*S*)- hydroxypyrrolidine (21.1 μL, 0.254 mmol), acetic acid (21.9 μL, 0.382 mmol), and sodium cyanoborohydride (16.8 mg, 0.254 mmol) in DMF (4.0 mL). The crude was purified by preparative HPLC to yield 15.0 mg (35%) of the desired product **42** as a formate salt. ^1^H NMR (500 MHz, DMSO-*d*_6_): δ 10.85 (s, 1H), 8.17 (s, 1H), 8.15–8.10 (m, 1H), 8.12 (d, *J* = 1.7 Hz, 1H), 8.01 (dd, *J* = 8.0, 1.7 Hz, 1H), 7.88 (d, *J* = 8.0 Hz, 1H), 7.58 (d, *J* = 8.4 Hz, 1H), 7.37 (d *J* = 10.9 Hz, 1H), 5.27 (s, 2H), 4.17–4.13 (m, 1H), 3.51 (d, *J* = 14.3 Hz, 1H), 3.46 (d, *J* = 14.3 Hz, 1H), 3.34 (s, 3H), 2.62 (dd, *J* = 9.8, 6.1 Hz, 1H), 2.57 (q, *J* = 7.5 Hz, 1H), 2.39–2.35 (m, 1H), 2.32 (dd, *J* = 9.8, 3.5 Hz, 1H), 1.99–1.92 (m, 1H), 1.54–1.48 (m, 1H); ^13^C NMR (125 MHz, DMSO-*d*_6_): δ 164.4, 163.6, 157.5 (d, *J* = 242.0 Hz), 156.1 (d, *J* = 9.0 Hz), 149.8–149.7 (m), 147.8–147.6 (m), 146.4–146.1 (m), 144.5–144.2 (m), 143.2, 140.4, 133.2 131.0, 130.1, 128.0, 125.8, 125.7 (d, *J* = 3.2 Hz), 122.0–121.7 (m), 113.9 (dd, *J* = 19.0, 16.3 Hz), 113.5 (d, *J* = 22.1 Hz), 102.6 (d, *J* = 26.1 Hz), 98.7 (d, *J* = 20.9 Hz), 69.4, 62.3, 59.1, 52.2, 51.7, 43.1, 34.4; ^19^F NMR (471 MHz, DMSO-*d*_6_): δ −107.9, −127.6, −141.0, −141.8; *R*_*f*_ HPLC: 8.0 min (13 min from 10 to 95% MeCN in water (0.1% formic acid), then 7 min 95% MeCN) > 99.9% purity; HRMS (MALDI): *m*/*z* found, 665.0131 [M + H]^+^ (calcd C_26_H_23_BrClF_4_N_2_O_5_S^+^ 665.0130).

##### (*S*)-1-(5-Bromo-2-((6-bromo-3-(2-chloro-4-(methylsulfonyl)­benzamido)-2-fluorobenzyl)­oxy)-4-fluorobenzyl)­pyrrolidine-3-carboxamide (**43**)

The synthesis was performed according to GP6 using **11m** (45.9 mg, 0.0720 mmol), (*S*)- pyrrolidine-3-carboxamide hydrochloride (43.4 mg, 0.288 mmol), acetic acid (24.8 μL, 0.432 mmol), and sodium triacetoxyborohydride (76.3 mg, 0.288 mmol) in DMF (4.0 mL). The crude was purified by preparative HPLC to yield 36.7 mg (69%) of the desired product **43** as a formate salt. ^1^H NMR (500 MHz, DMSO-*d*_6_): δ 10.75 (s, 1H), 8.15 (s, 1H), 8.12 (d, *J* = 1.7 Hz, 1H), 8.01 (dd, *J* = 8.0, 1.7 Hz, 1H), 7.97 (t, *J* = 8.4 Hz, 1H), 7.89 (d, *J* = 8.0 Hz, 1H), 7.63 (d, *J* = 8.8 Hz, 1H), 7.59 (d *J* = 8.3 Hz, 1H), 7.40 (d, *J* = 10.9 Hz, 1H), 7.22 (br s, 1H), 6.75 (br s, 1H), 5.22 (s, 2H), 3.54 (s, 2H), 3.34 (s, 3H), 2.83–2.74 (m, 2H), 2.62–2.53 (m, 1H), 2.50–2.48 (m, 1H), 2.44 (q, *J* = 8.4 Hz, 1H), 1.89–1.84 (m, 2H); ^13^C NMR (125 MHz, DMSO-*d*_6_): δ 175.4, 164.3, 163.2, 157.7 (d, *J* = 242.0 Hz), 156.4 (dd, *J* = 9.0 Hz), 153.3 (d, *J* = 252.8 Hz), 143.1, 140.6, 133.2, 131.0, 130.0, 128.6 (d, *J* = 3.5 Hz), 128.0, 126.6, 125.8, 125.23 (d, *J* = 9.6 Hz), 125.16, 123.4 (d, *J* = 15.5 Hz), 121.0 (d, *J* = 2.9 Hz), 102.4 (d, *J* = 26.1 Hz), 98.4 (d, *J* = 20.9 Hz), 64.5, 56.7, 53.3, 51.3, 43.1, 42.2, 27.5; ^19^F NMR (471 MHz, DMSO-*d*_6_): δ −107.6, −121.4; *R*_*f*_ HPLC: 8.3 min (13 min from 10 to 95% MeCN in water (0.1% formic acid), then 7 min 95% MeCN). 95.2% purity; HRMS (MALDI): *m*/*z* found, 733.9557 [M + H]^+^ (calcd C_27_H_25_Br_2_ClF_2_N_3_O_5_S^+^ 733.9533).

##### Methyl (*S*)-1-(5-Bromo-2-((3-(2-chloro-4-(methylsulfonyl)­benzamido)-2,6-difluorobenzyl)­oxy)-4-fluorobenzyl)­pyrrolidine-3-carboxylate (**44**)

The synthesis was performed according to GP6 using **11p** (159 mg, 0.275 mmol), (*S*)-methyl pyrrolidine-3-carboxylate hydrochloride (182 mg, 1.10 mmol), acetic acid (94.6 μL, 1.65 mmol), and sodium cyanoborohydride (72.8 mg, 1.10 mmol) in DMF (10.0 mL). The crude was purified by preparative HPLC to yield 84.1 mg (44%) of the desired product **44**. ^1^H NMR (500 MHz, DMSO-*d*_6_): δ 10.63 (s, 1H), 8.12 (d, *J* = 1.7 Hz, 1H), 8.01 (dd, *J* = 8.0, 1.7 Hz, 1H), 7.94–7.89 (m, 1H), 7.88 (d, *J* = 8.0 Hz, 1H), 7.56 (d, *J* = 8.4 Hz, 1H), 7.36 (d *J* = 10.9 Hz, 1H), 7.25 (t, *J* = 9.0 Hz, 1H), 5.22 (s, 2H), 3.59 (s, 3H), 3.50 (d, *J* = 14.3 Hz, 1H), 3.45 (d, *J* = 14.3 Hz, 1H), 3.35 (s, 3H), 3.01–2.95 (m, 1H), 2.62 (d, *J* = 7.1 Hz, 2H), 2.49–2.43 (m, 2H), 2.00–1.87 (m, 2H); ^13^C NMR (125 MHz, DMSO-*d*_6_): δ 174.7, 164.3, 158.2 (dd, *J* = 246.9, 6.4 Hz), 157.6 (d, *J* = 241.8 Hz), 156.2 (d, *J* = 9.0 Hz), 153.7 (dd. *J* = 251.5, 7.6 Hz), 143.1, 140.7, 133.1, 131.0, 130.0, 128.0, 127.2 (d, *J* = 9.1 Hz), 125.8, 125.5, 121.7 (dd, *J* = 12.8, 3.5 Hz), 112.2 (dd, *J* = 20.1, 17.8 Hz), 111.4 (dd, *J* = 22.7, 2.6 Hz), 102.4 (d, *J* = 26.1 Hz), 98.4 (d, *J* = 20.9 Hz), 59.1, 55.7, 52.9, 51.6, 51.0, 43.1, 41.2, 27.1; ^19^F NMR (471 MHz, DMSO-*d*_6_): δ −107.9, −117.6, −122.1; *R*_*f*_ HPLC: 8.1 min (13 min from 10 to 95% MeCN in water (0.1% formic acid), then 7 min 95% MeCN). 95.5% purity; HRMS (MALDI): *m*/*z* found, 689.0340 [M + H]^+^ (calcd C_28_H_26_BrClF_3_N_2_O_6_S^+^ 689.0330).

##### (*S*)-*N*-(3-((4-Bromo-2-fluoro-6-((3-hydroxypyrrolidin-1-yl)­methyl)­phenoxy)­methyl)-2,4,5-trifluorophenyl)-2-chloro-4-(methylsulfonyl)­benzamide (**45**)

The synthesis was performed according to GP6 using **11r** (128 mg, 0.215 mmol), (*S*)-hydroxypyrrolidine (71.4 μL, 0.859 mmol), acetic acid (73.9 μL, 1.29 mmol), and sodium cyanoborohydride (54.0 mg, 0.859 mmol) in DMF (5.0 mL). The crude was purified by preparative HPLC to yield 109 mg (76%) of the desired product **45** as a formate salt. ^1^H NMR (500 MHz, DMSO-*d*_6_): δ 10.84 (s, 1H), 8.15 (s, 1H), 8.12 (d, *J* = 1.6 Hz, 1H), 8.13–8.10 (m, 1H), 8.01 (dd, *J* = 8.0, 1.6 Hz, 1H), 7.88 (d, *J* = 8.0 Hz, 1H), 7.55 (dd, *J* = 10.5, 2.3 Hz, 1H), 7.41 (s, 1H), 5.22 (s, 2H), 4.20–4.16 (m, 1H), 3.59 (d, *J* = 13.8 Hz, 1H), 3.52 (d, *J* = 13.8 Hz, 1H), 3.35 (s, 3H), 2.65 (dd, *J* = 9.8, 6.1 Hz, 1H), 2.60 (q, *J* = 7.7 Hz, 1H), 2.43–2.38 (m, 1H), 2.35 (dd, *J* = 9.7, 3.5 Hz, 1H), 2.01–1.94 (m, 1H), 1.58–1.52 (m, 1H); ^13^C NMR (125 MHz, DMSO-*d*_6_): δ 164.4, 163.3, 155.3 (d, *J* = 250.4 Hz), 148.9 (d, *J* = 250.1 Hz), 145.4 (ddd, *J* = 249.1, 14.1, 6.8 Hz), 145.2 (ddd, *J* = 242.1, 13.0, 3.0 Hz), 143.2, 142.7 (d, *J* = 11.2 Hz), 140.4, 136.2, 131.0, 130.1, 128.3 (d, *J* = 2.7 Hz), 128.0, 125.8, 121.9–121.7 (m), 118.8 (d, *J* = 22.7 Hz), 115.7 (d, *J* = 9.6 Hz), 114.3 (dd, *J* = 20.1, 16.9 Hz), 113.5 (d, *J* = 22.4 Hz), 69.3, 62.6, 62.3, 52.5, 52.3, 43.1, 34.3; ^19^F NMR (471 MHz, DMSO-*d*_6_): δ −126.7, −127.9, −141.1, −142.2; *R*_*f*_ HPLC: 8.1 min (13 min from 10 to 95% MeCN in water (0.1% formic acid), then 7 min 95% MeCN). 99.0% purity; HRMS (H-ESI): *m*/*z* found, 665.0137 [M + H]^+^ (calcd C_26_H_22_BrClF_4_N_2_O_5_S^+^ 665.01302).

##### *N*-(4-((4-Bromo-2-(((2-hydroxyethyl)­amino)­methyl)­phenoxy)­methyl)­phenyl)-2-chloro-4-(methylsulfonyl)­benzamide (**50**)

The synthesis was performed according to GP6 using **49** (110 mg, 0.210 mmol), ethanolamine (51.2 μL, 0.840 mmol), acetic acid (72.1 μL, 1.26 mmol), and sodium cyanoborohydride (55.6 mg, 0.840 mmol) in DMF (8.0 mL). The crude was purified by preparative HPLC to yield 24.7 mg (21%) of the desired product **50** as a formate salt. ^1^H NMR (500 MHz, DMSO-*d*_6_): δ 10.76 (s, 1H), 8.26 (br s, 1H), 8.12 (d, *J* = 1.7 Hz, 1H), 8.00 (dd, *J* = 8.0, 1.7 Hz, 1H), 7.88 (d, *J* = 8.0 Hz, 1H), 7.72 (d, *J* = 8.6 Hz, 2H), 7.56 (d, *J* = 2.5 Hz, 1H), 7.47 (d, *J* = 8.6 Hz, 2H), 7.42 (dd, *J* = 8.8, 2.6 Hz, 1H), 7.05 (d, *J* = 8.9 Hz, 1H), 5.12 (s, 2H), 3.85 (s, 2H), 3.53 (t, *J* = 5.6 Hz, 2H), 3.34 (s, 3H), 2.69 (t, *J* = 5.5 Hz, 2H); ^13^C NMR (125 MHz, DMSO-*d*_6_): δ 164.4, 163.7, 155.4, 143.0, 141.2, 138.2, 132.4, 131.9, 131.0, 130.9, 129.9, 129.0, 128.3, 128.1, 125.9, 119.6, 114.4, 112.0, 69.4, 59.1, 50.3, 46.0, 43.1; *R*_*f*_ HPLC: 7.9 min (13 min from 10 to 95% MeCN in water (0.1% formic acid), then 7 min 95% MeCN). 95.0% purity; HRMS (MALDI): *m*/*z* found, 567.0344 [M + H]^+^ (calcd C_24_H_25_BrClN_2_O_5_S^+^ 567.0351).

#### Synthesis of Final Compounds **55a**–**55d**

##### *N*-(2-Chloro-4-(methylsulfonyl)­phenyl)-3-(chloromethyl)­benzamide (**53a**)

3-(Chloromethyl)­benzoyl chloride (227 mg, 1.20 mmol) was added to a solution of 2-chloro-4-(methylsulfonyl)­aniline (206 mg, 1.0 mmol) in DCM (12.0 mL) at 0 °C under Ar. Then, triethylamine (169 μL, 1.20 mmol) was added, and the resulting solution was stirred at rt for 2 h. The reaction mixture was diluted with DCM and washed with saturated aqueous sodium bicarbonate solution. The organic phase was washed with brine, filtered, and evaporated. The crude was purified by flash chromatography (hexane/EtOAc, 9:1 to 6:4) to yield 125 mg (35%) of the desired product **53a**. ^1^H NMR (250 MHz, CDCl_3_): δ 8.84 (d, *J* = 8.7 Hz, 1H), 8.64 (br s, 1H), 8.03 (d, *J* = 2.1 Hz, 1H), 7.97 (t, *J* = 1.4 Hz, 1H), 7.90 (dd *J* = 8.8, 2.0 Hz, 1H), 7.86 (dt, *J* = 7.8, 1.4 Hz, 1H), 7.66 (dt, *J* = 7.7, 1.3 Hz, 1H), 7.55 (t, *J* = 7.7 Hz, 1H), 4.67 (s, 2H), 3.08 (s, 3H).

##### *N*-(2-Chloro-4-(methylsulfonyl)­phenyl)-4-(chloromethyl)­benzamide (**53b**)

4-(Chloromethyl)­benzoyl chloride (552 mg, 2.92 mmol) was added to a solution of 2-chloro-4-(methylsulfonyl)­aniline (500 mg, 2.43 mmol) in DCM (15.0 mL) at 0 °C under Ar. Then, triethylamine (410 μL, 2.92 mmol) was added, and the resulting solution was stirred at rt overnight. The reaction mixture was diluted with DCM and washed with saturated aqueous sodium bicarbonate solution. The organic phase was washed with brine, filtered, and evaporated. The crude was purified by flash chromatography (hexane/EtOAc, 7:3 to 5:5) to yield 599 mg (69%) of the desired product **53b**. ^1^H NMR (300 MHz, CDCl_3_): δ 8.86 (d, *J* = 8.8 Hz, 1H), 8.64 (br s, 1H), 8.03 (d, *J* = 2.0 Hz, 1H), 7.93 (d, *J* = 8.1 Hz, 2H), 7.90 (dd *J* = 8.8, 2.0 Hz, 1H), 7.58 (d, *J* = 8.2 Hz, 2H), 4.66 (s, 2H), 3.08 (s, 3H).

##### 3-((4-Bromo-2-formylphenoxy)­methyl)-*N*-(2-chloro-4-(methylsulfonyl)­phenyl)­benzamide (**54a**)

The synthesis was performed according to GP5 using **53a** (140 mg, 0.391 mmol), 5-bromosalicylaldehyde (94.3 mg, 0.469 mmol), and potassium carbonate (109 mg, 0.782 mmol) in DMF (6.0 mL). The crude was purified by flash chromatography (hexane/EtOAc, 8:2 to 5:5) to yield 172 mg (84%) of the desired product **54a**. ^1^H NMR (250 MHz, DMSO-*d*_6_): δ 10.38 (s, 1H), 10.32 (s, 1H), 8.12–8.09 (m, 2H), 8.02–7.91 (m, 3H), 7.87–7.77 (m, 3H), 7.61 (t, *J* = 7.7 Hz, 1H), 7.35 (d, *J* = 8.9 Hz, 1H), 5.42 (s, 2H), 3.31 (s, 3H).

##### 4-((4-Bromo-2-formylphenoxy)­methyl)-*N*-(2-chloro-4-(methylsulfonyl)­phenyl)­benzamide (**54b**)

The synthesis was performed according to GP5 using **53b** (200 mg, 0.558 mmol), 5-bromosalicylaldehyde (135 mg, 0.670 mmol), and potassium carbonate (156 mg, 1.12 mmol) in DMF (10.0 mL). The crude was purified by flash chromatography (hexane/EtOAc, 6:4 to 3:7) to yield 206 mg (71%) of the desired product **54b**. ^1^H NMR (300 MHz, CDCl_3_): δ 10.48 (s, 1H), 8.86 (d, *J* = 8.8 Hz, 1H), 8.66 (br s, 1H), 8.03 (d, *J* = 2.0 Hz, 1H), 7.98 (d, *J* = 8.5 Hz, 2H), 7.96 (dd, *J* = 8.5, 2.0 Hz, 1H), 7.90 (dd, *J* = 8.7, 1.9 Hz, 1H), 7.65–7.60 (m, 3H), 6.93 (d, *J* = 8.9 Hz, 1H), 5.29 (s, 2H), 3.08 (s, 3H).

##### 4-((4-Bromo-2-(((2-hydroxyethyl)­amino)­methyl)­phenoxy)­methyl)-*N*-(2-chloro-4-(methylsulfonyl)­phenyl)­benzamide (**55a**)

The synthesis was performed according to GP6 using **54a** (165 mg, 0.316 mmol), ethanolamine (77.1 μL, 1.26 mmol), acetic acid (108 μL, 1.90 mmol), and sodium cyanoborohydride (83.6 mg, 1.26 mmol) in DMF (10.0 mL). The crude was purified by preparative HPLC to yield 55.5 mg (31%) of the desired product **55a** as a formate salt. ^1^H NMR (500 MHz, DMSO-*d*_6_): δ 10.43 (br s, 1H), 8.29 (br s, 1H), 8.11 (s, 1H), 8.10 (d, *J* = 2.1 Hz, 1H), 7.99 (d, *J* = 8.5 Hz, 1H), 7.98 (dd, *J* = 7.7 Hz, 1H), 7.94 (dd, *J* = 8.5, 2.1 Hz, 1H), 7.75 (d *J* = 7.7 Hz, 1H), 7.61–7.58 (m, 2H), 7.46 (dd, *J* = 8.8, 2.6 Hz, 1H), 7.09 (d, *J* = 8.9 Hz, 1H), 5.26 (s, 2H), 3.94 (s, 2H), 3.54 (t, *J* = 5.6 Hz, 2H), 3.32 (s, 3H), 2.74 (t, *J* = 5.1 Hz, 2H); ^13^C NMR (125 MHz, DMSO-*d*_6_): δ 165.4, 164.9, 155.4, 139.9, 138.7, 137.4, 133.8, 132.3, 131.23, 131.16, 129.0, 128.8, 128.4, 128.2, 127.7, 127.4, 126.9, 126.3, 114.4, 112.2, 69.2, 58.7, 50.1, 45.7, 43.4; *R*_*f*_ HPLC: 7.8 min (13 min from 10 to 95% MeCN in water (0.1% formic acid), then 7 min 95% MeCN). 99.2% purity; HRMS (MALDI): *m*/*z* found, 567.0349 [M + H]^+^ (calcd C_24_H_25_BrClN_2_O_5_S^+^ 567.0351).

##### 4-((4-Bromo-2-(((2-hydroxyethyl)­amino)­methyl)­phenoxy)­methyl)-*N*-(2-chloro-4-(methylsulfonyl)­phenyl)­benzamide (**55b**)

The synthesis was performed according to GP6 using **54b** (36.0 mg, 0.0689 mmol), ethanolamine (16.8 μL, 0.276 mmol), acetic acid (23.6 μL, 0.413 mmol), and sodium cyanoborohydride (18.3 mg, 0.276 mmol) in DMF (4.0 mL). The crude was purified by preparative HPLC to yield 12.8 mg (33%) of the desired product **55b** as a formate salt. ^1^H NMR (500 MHz, DMSO-*d*_6_): δ 10.30 (s, 1H), 8.25 (br s, 1H), 8.09 (d, *J* = 2.1 Hz, 1H), 8.03 (d, *J* = 8.3 Hz, 2H), 7.99 (d, *J* = 8.5 Hz, 1H), 7.93 (dd, *J* = 8.5, 2.1 Hz, 1H), 7.65 (d, *J* = 8.3 Hz, 2H), 7.57 (d *J* = 2.4 Hz, 1H), 7.42 (dd, *J* = 8.7, 2.5 Hz, 1H), 7.04 (d, *J* = 8.9 Hz, 1H), 5.27 (s, 2H), 3.87 (s, 2H), 3.53 (t, *J* = 5.6 Hz, 2H), 3.31 (s, 3H), 2.69 (t, *J* = 5.1 Hz, 2H); ^13^C NMR (125 MHz, DMSO-*d*_6_): δ 165.2, 164.2, 155.2, 141.2, 139.8, 138.7, 132.9, 131.9, 130.7, 129.7, 128.9, 128.3, 128.1, 127.6, 127.3, 126.3, 114.3, 112.2, 69.0, 59.4, 50.6, 46.3, 43.4; *R*_*f*_ HPLC: 7.9 min (13 min from 10 to 95% MeCN in water (0.1% formic acid), then 7 min 95% MeCN). 95.9% purity; HRMS (MALDI): *m*/*z* found, 567.0351 [M + H]^+^ (calcd C_24_H_25_BrClN_2_O_5_S^+^ 567.0351).

#### Synthesis of Final Compounds **58a**–**58j**

##### *N*-(3-((4-Bromo-2-formylphenoxy)­methyl)­phenyl)­acetamide (**57a**)

The synthesis was performed according to GP5 using *N*-[3-(chloromethyl)­phenyl]­acetamide (100 mg, 0.545 mmol), 5-bromosalicylaldehyde (131 mg, 0.654 mmol), and potassium carbonate (152 mg, 1.09 mmol) in DMF (8.0 mL). The crude was purified by flash chromatography (hexane/EtOAc, 5:5 to 1:9) to yield 167 mg (88%) of the desired product **57a**. ^1^H NMR (250 MHz, CDCl_3_): δ 10.45 (s, 1H), 7.92 (d, *J* = 2.6 Hz, 1H), 7.69 (s, 1H), 7.59 (dd, *J* = 8.9, 2.6 Hz, 1H), 7.50 (br s, 1H), 7.42 (d, *J* = 8.3 Hz, 1H), 7.33 (t, *J* = 8.0 Hz, 1H), 7.14 (d, *J* = 7.5 Hz, 1H), 6.91 (d, *J* = 8.9 Hz, 1H), 5.15 (s, 2H), 2.18 (s, 3H).

##### *N*-(3-((4-Bromo-2-formylphenoxy)­methyl)­phenyl)-2,2,2-trifluoroacetamide (**57b**)

The synthesis was performed according to GP5 using **56b/c** (108 mg, 0.456 mmol), 5-bromosalicylaldehyde (111 mg, 0.550 mmol), and potassium carbonate (127 mg, 0.912 mmol) in DMF (8.0 mL). The crude was purified by flash chromatography (hexane/EtOAc, 9:1 to 7:3) to yield 167 mg (88%) of the desired product **57b**. ^1^H NMR (250 MHz, CDCl_3_): δ 11.30 (s, 1H), 10.38 (s, 1H), 7.83 (dd, *J* = 9.5, 2.7 Hz, 1H), 7.81 (s, 1H), 7.78 (d, *J* = 2.7 Hz, 1H), 7.66 (d, *J* = 8.4 Hz, 1H), 7.45 (t, *J* = 7.9 Hz, 1H), 7.35 (d, *J* = 7.8 Hz, 1H), 7.32 (d, *J* = 8.9 Hz, 1H), 5.33 (s, 2H).

##### *N*-(3-((4-Bromo-5-fluoro-2-formylphenoxy)­methyl)­phenyl)-2,2,2-trifluoroacetamide (**57c**)

The synthesis was performed according to GP5 using **56b/c** (245 mg, 1.03 mmol), 5-bromo-4-fluoro-2-hydroxybenzaldehyde (271 mg, 1.24 mmol), and potassium carbonate (288 mg, 2.06 mmol) in DMF (10.0 mL). The crude was purified by flash chromatography (hexane/EtOAc, 3:1 to 2:1) to yield 110 mg (25%) of the desired product **57c**. ^1^H NMR (250 MHz, DMSO-*d*_6_): δ 10.28 (s, 1H), 7.94 (d, *J* = 8.2 Hz, 1H), 7.81 (s, 1H), 7.66 (d, *J* = 8.8 Hz, 1H), 7.51 (d, *J* = 11.1 Hz, 1H), 7.46 (t, *J* = 7.8 Hz, 1H), 7.36 (d, *J* = 7.6 Hz, 1H), 5.34 (s, 2H).

##### *N*-(3-((4-Bromo-5-fluoro-2-formylphenoxy)­methyl)-5-fluorophenyl)-2,2,2-trifluoroacetamide (**57d**)

The synthesis was performed according to GP5 using **56d** (100 mg, 0.391 mmol), 5-bromo-4-fluoro-2-hydroxybenzaldehyde (103 mg, 0.469 mmol), and potassium carbonate (109 mg, 0.782 mmol) in DMF (6.0 mL). The crude was purified by flash chromatography (hexane/EtOAc, 8:2 to 6:4) to yield 41.0 mg (24%) of the desired product **57d**. ^1^H NMR (250 MHz, DMSO-*d*_6_): δ 11.48 (br s, 1H), 10.30 (s, 1H), 7.96 (d, *J* = 8.2 Hz, 1H), 7.64 (s, 1H), 7.66 (d, *J* = 8.8 Hz, 1H), 7.57 (dt, *J* = 10.7, 2.3 Hz, 1H), 7.48 (d, *J* = 11.0 Hz, 1H), 7.25 (d, *J* = 9.3 Hz, 1H), 5.35 (s, 2H).

##### *N*-(3-((4-Bromo-5-fluoro-2-formylphenoxy)­methyl)-2,4-difluorophenyl)-2,2,2-trifluoroacetamide (**57e/f**)

The synthesis was performed according to GP5 using **56e/f** (527 mg, 1.93 mmol), 5-bromo-4-fluoro-2-hydroxybenzaldehyde (507 mg, 2.32 mmol), and potassium carbonate (539 mg, 3.86 mmol) in DMF (30.0 mL). The crude was purified by flash chromatography (hexane/EtOAc, 9:1 to 7:3) to yield 494 mg (56%) of the desired product **57e/f**. ^1^H NMR (300 MHz, DMSO-*d*_6_): δ 11.36 (br s, 1H), 10.07 (s, 1H), 7.93 (d, *J* = 8.2 Hz, 1H), 7.66 (d, *J* = 10.9 Hz, 1H), 7.65 (td, *J* = 8.9, 5.9 Hz, 1H), 7.30 (td, *J* = 9.0, 1.5 Hz, 1H), 5.40 (s, 2H).

##### 2-((1*H*-Benzo­[*d*]­imidazole-2-yl)­methoxy)-5-bromobenzaldehyde (**57g/h**)

The synthesis was performed according to GP5 using 2-(chlormethyl)­benzimidazole (150 mg, 0.90 mmol), 5-bromosalicylaldehyde (217 mg, 1.08 mmol), and potassium carbonate (251 mg, 1.80 mmol) in DMF (8.0 mL). The crude was purified by flash chromatography (hexane/EtOAc, 5:5 to 1:9) to yield 167 mg (88%) of the desired product **57g/h**. ^1^H NMR (250 MHz, DMSO-*d*_6_): δ 12.66 (s, 1H), 10.43 (s, 1H), 7.84 (dd, *J* = 8.8, 2.6 Hz, 1H), 7.78 (d, *J* = 2.6 Hz, 1H), 7.65–7.61 (m, 1H), 7.52–7.48 (m, 1H), 7.40 (d, *J* = 8.9 Hz, 1H), 7.23–7.17 (m, 2H), 5.55 (s, 2H).

##### 2-((1*H*-Benzo­[d]­imidazole-6-yl)­methoxy)-5-bromobenzaldehyde (**57i**)

The synthesis was performed according to GP5 using **56i** (100 mg, 0.60 mmol), 5-bromosalicylaldehyde (145 mg, 0.72 mmol), and potassium carbonate (167 mg, 1.20 mmol) in DMF (8.0 mL). The crude was purified by flash chromatography (EtOAc) to yield 64.3 mg (32%) of the desired product **57i**. ^1^H NMR (250 MHz, DMSO-*d*_6_): δ 12.48 (br s, 1H), 10.32 (s, 1H), 8.23 (s, 1H), 7.81 (dd, *J* = 8.9, 2.7 Hz, 1H), 7.75 (d, *J* = 2.6 Hz, 1H), 7.75–7.73 (m, 1H), 7.38 (d, *J* = 8.9 Hz, 1H), 7.33 (d, *J* = 8.8 Hz, 1H), 5.40 (s, 2H).

##### 5-Bromo-2-(imidazo­[1,2-*a*]­pyridin-7-ylmethoxy)­benzaldehyde (**57j**)

The synthesis was performed according to GP5 using **56j** (112 mg, 0.675 mmol), 5-bromosalicylaldehyde (163 mg, 0.810 mmol), and potassium carbonate (188 mg, 1.35 mmol) in DMF (8.0 mL). The crude was purified by flash chromatography (EtOAc) to yield 64.3 mg (32%) of the desired product **57j**. ^1^H NMR (250 MHz, DMSO-*d*_6_): δ 10.37 (br s, 1H), 8.56 (d, *J* = 7.0 Hz, 1H), 7.94 (s, 1H), 7.82 (dd, *J* = 8.8, 2.7 Hz, 1H), 7.78 (d, *J* = 2.7 Hz, 1H), 7.71 (s, 1H), 7.58 (d, *J* = 1.1 Hz, 1H), 7.33 (d, *J* = 8.8 Hz, 1H), 7.02 (dd, *J* = 7.0, 1.6 Hz, 1H), 5.36 (s, 2H).

##### (*S*)-*N*-(3-((4-Bromo-2-((3-hydroxypyrrolidin-1-yl)­methyl)­phenoxy)­methyl)­phenyl)­acetamide (**58a**)

The synthesis was performed according to GP6 using **57a** (83.0 mg, 0.238 mmol), (*S*)-(−)-hydroxypyrrolidine (79.1 μL, 0.952 mmol), acetic acid (81.7 μL, 1.43 mmol), and sodium cyanoborohydride (63.0 mg, 0.952 mmol) in DMF (6.0 mL). The crude was purified by preparative HPLC to yield 70.0 mg (70%) of the desired product **58a** as a formate salt. ^1^H NMR (500 MHz, DMSO-*d*_6_): δ 9.97 (br s, 1H), 8.20 (br s, 1H), 7.70 (s, 1H), 7.50–7.47 (m, 2H), 7.36 (dd, *J* = 8.7, 2.7 Hz, 1H), 7.29 (t, *J* = 7.9 Hz, 1H), 7.10 (d, *J* = 7.7 Hz, 1H), 7.00 (d *J* = 8.8 Hz, 1H), 5.08 (s, 2H), 4.22–4.19 (m, 1H), 3.67 (d, *J* = 14.4 Hz, 1H), 3.62 (d, *J* = 14.4 Hz, 1H), 2.71 (dd, *J* = 9.8, 6.1 Hz, 1H), 2.67 (q, *J* = 7.8 Hz, 1H), 2.49–2.44 (m, 1H), 2.42 (dd, *J* = 9.8, 3.5 Hz, 1H), 2.04 (s, 3H), 2.04–1.97 (m, 1H), 1.59–1.53 (m, 1H); ^13^C NMR (125 MHz, DMSO-*d*_6_): δ 168.5, 163.8, 155.3, 139.5, 137.5, 132.0, 130.4, 129.8, 128.9, 121.9, 118.5, 117.8, 114.5, 112.2, 69.6, 69.5, 62.6, 52.7, 52.5, 34.4, 24.1; *R*_*f*_ HPLC: 7.1 min (13 min from 10 to 95% MeCN in water (0.1% formic acid), then 7 min 95% MeCN). 96.7% purity; HRMS (MALDI): *m*/*z* found, 419.0982 [M + H]^+^ (calcd C_20_H_24_BrN_2_O_3_^+^ 419.0965).

##### (*S*)-*N*-(3-((4-Bromo-2-((3-hydroxypyrrolidin-1-yl)­methyl)­phenoxy)­methyl)­phenyl)-2,2,2-trifluoroacetamide (**58b**)

The synthesis was performed according to GP6 using **57b** (100 mg, 0.249 mmol), (*S*)-(−)-hydroxypyrrolidine (82.8 μL, 0.996 mmol), acetic acid (85.4 μL, 1.49 mmol), and sodium cyanoborohydride (65.9 mg, 0.996 mmol) in DMF (6.0 mL). The crude was purified by preparative HPLC to yield 52.1 mg (44%) of the desired product **58b** as a formate salt. ^1^H NMR (500 MHz, DMSO-*d*_6_): δ 11.33 (br s, 1H), 8.20 (s, 1H), 7.81 (s, 1H), 7.60 (dd, *J* = 8.1, 1.0 Hz, 1H), 7.50 (d, *J* = 2.6 Hz, 1H), 7.43 (t, *J* = 7.9 Hz, 1H), 7.38 (dd, *J* = 8.7, 2.6 Hz, 1H), 7.31 (d, *J* = 7.7 Hz, 1H), 7.02 (d, *J* = 8.8 Hz, 1H), 5.15 (s, 2H), 4.23–4.19 (m, 1H), 3.72 (d, *J* = 14.3 Hz, 1H), 3.68 (d, *J* = 14.3 Hz, 1H), 2.77–2.69 (m, 2H), 2.54–2.50 (m, 1H), 2.46 (dd, *J* = 10.0, 3.4 Hz, 1H), 2.04–1.97 (m, 1H), 1.61–1.55 (m, 1H); ^13^C NMR (125 MHz, DMSO-*d*_6_): δ 163.8, 155.2, 154.5 (q, *J* = 36.7 Hz), 138.0, 136.5, 132.2, 130.5, 129.3, 129.1, 124.4, 120.5, 119.7, 115.8 (q, *J* = 287.0 Hz), 114.5, 112.2, 69.34, 69.25, 62.4, 52.5, 52.4, 34.3; ^19^F NMR (471 MHz, DMSO-*d*_6_): δ −73.9; *R*_*f*_ HPLC: 8.0 min (13 min from 10 to 95% MeCN in water (0.1% formic acid), then 7 min 95% MeCN). 99.8% purity; HRMS (MALDI): *m*/*z* found, 473.0680 [M + H]^+^ (calcd C_20_H_21_BrF_3_N_2_O_3_^+^ 473.0682).

##### (*S*)-*N*-(3-((4-Bromo-5-fluoro-2-((3-hydroxypyrrolidin-1-yl)­methyl)­phenoxy)­methyl)­phenyl)-2,2,2-trifluoroacetamide (**58c**)

The synthesis was performed according to GP6 using **57c** (90 mg, 0.214 mmol), (*S*)-(−)-hydroxypyrrolidin (71.5 μL, 0.860 mmol), acetic acid (73.4 μL, 1.28 mmol), and sodium triacetoxyborohydride (228 mg, 0.860 mmol) in DMF (8.0 mL). The crude was purified by preparative HPLC to yield 40.0 mg (38%) of the desired product **58c** as a formate salt. ^1^H NMR (500 MHz, DMSO-*d*_6_): δ 11.32 (br s, 1H), 8.18 (s, 1H), 7.81 (s, 1H), 7.61–7.58 (m, 2H), 7.44 (t, *J* = 7.9 Hz, 1H), 7.32 (d, *J* = 7.7 Hz, 1H), 7.20 (d, *J* = 11.1 Hz, 1H), 5.18 (s, 2H), 4.22–4.18 (m, 1H), 3.69 (d, *J* = 14.1 Hz, 1H), 3.64 (d, *J* = 14.1 Hz, 1H), 2.74 (dd, *J* = 9.9, 6.0 Hz, 1H), 2.70 (q, *J* = 7.6 Hz, 1H), 2.52–2.48 (m, 1H), 2.44 (dd, *J* = 10.0, 3.5 Hz, 1H), 2.03–1.96 (m, 1H), 1.60–1.54 (m, 1H); ^13^C NMR (125 MHz, DMSO-*d*_6_): δ 163.9, 158.2 (d, *J* = 241.6 Hz), 156.9 (d, *J* = 9.0 Hz), 155.0 (q, *J* = 36.7 Hz), 137.9, 137.0, 133.8, 129.7, 125.3 (d, *J* = 3.1 Hz), 125.0, 121.1, 120.3, 116.2 (q, 287.0 Hz), 102.6 (d, *J* = 26.1 Hz), 98.2 (d, *J* = 20.9 Hz), 70.1, 69.8, 62.7, 52.7, 52.5, 34.7; ^19^F NMR (471 MHz, DMSO-*d*_6_): δ −73.9, −107.9; *R*_*f*_ HPLC: 8.3 min (13 min from 10 to 95% MeCN in water (0.1% formic acid), then 7 min 95% MeCN). 98.9% purity; HRMS (MALDI): *m*/*z* found, 513.0399 [M + Na]^+^ (calcd C_20_H_19_BrF_4_N_2_NaO_3_^+^ 513.0407).

##### (*S*)-*N*-(3-((4-Bromo-5-fluoro-2-((3-hydroxypyrrolidin-1-yl)­methyl)­phenoxy)­methyl)-5-fluorophenyl)-2,2,2-trifluoroacetamide (**58d**)

The synthesis was performed according to GP6 using **57d** (80 mg, 0.183 mmol), (*S*)-(−)-hydroxypyrrolidin (60.9 μL, 0.732 mmol), acetic acid (62.9 μL, 1.10 mmol), and sodium triacetoxyborohydride (194 mg, 0.732 mmol) in DMF (8.0 mL). The crude was purified by preparative HPLC to yield 50.0 mg (54%) of the desired product **58d** as a formate salt. ^1^H NMR (500 MHz, DMSO-*d*_6_): δ 11.44 (br s, 1H), 8.16 (s, 1H), 7.64 (s, 1H), 7.59 (d, *J* = 8.4 Hz, 1H), 7.51 (dt, *J* = 10.7, 2.1 Hz, 1H), 7.20–7.17 (m, 2H), 5.20 (s, 2H), 4.22–4.18 (m, 1H), 3.68 (d, *J* = 14.0 Hz, 1H), 3.63 (d, *J* = 14.0 Hz, 1H), 2.73 (dd, *J* = 9.9, 6.0 Hz, 1H), 2.68 (q, *J* = 7.6 Hz, 1H), 2.52–2.47 (m, 1H), 2.42 (dd, *J* = 10.0, 3.5 Hz, 1H), 2.03–1.96 (m, 1H), 1.59–1.56 (m, 1H); ^13^C NMR (125 MHz, DMSO-*d*_6_): δ 163.4, 161.9 (d, *J* = 241.5 Hz), 157.7 (d, *J* = 241.7 Hz), 156.2 (d, *J* = 9.0 Hz), 154.7 (q, *J* = 37.1 Hz), 140.0 (d, *J* = 8.9 Hz), 138.1 (d, *J* = 11.3 Hz), 133.3, 125.1 (d, *J* = 3.1 Hz), 115.6 (q, 286.9 Hz), 115.3 (d, *J* = 2.4 Hz), 111.0 (d, *J* = 22.2 Hz), 107.4 (d, *J* = 26.2 Hz), 102.1 (d, *J* = 26.2 Hz), 97.9 (d, *J* = 20.1 Hz), 69.3, 69.0, 62.3, 52.3, 52.2, 34.3; ^19^F NMR (471 MHz, DMSO-*d*_6_): δ −73.9, −106.4, −111.2; *R*_*f*_ HPLC: 8.3 min (13 min from 10 to 95% MeCN in water (0.1% formic acid), then 7 min 95% MeCN). 97.8% purity; HRMS (MALDI): *m*/*z* found, 531.0318 [M + Na]^+^ (calcd C_20_H_18_BrF_5_N_2_NaO_3_^+^ 531.0313).

##### (*S*)-*N*-(3-((4-Bromo-5-fluoro-2-((3-hydroxypyrrolidin-1-yl)­methyl)­phenoxy)­methyl)-2,4-difluorophenyl)-2,2,2-trifluoroacetamide (**58e**)

The synthesis was performed according to GP6 using **57e/f** (100 mg, 0.219 mmol), (*S*)-(−)-hydroxypyrrolidin (72.8 μL, 0.876 mmol), acetic acid (75.3 μL, 1.31 mmol), and sodium cyanoborohydride (57.9 mg, 0.876 mmol) in DMF (10.0 mL). The crude was purified by preparative HPLC to yield 58.2 mg (50%) of the desired product 58e (Product decomposes with time). ^1^H NMR (500 MHz, DMSO-*d*_6_): δ 11.44 (br s, 1H), 8.16 (s, 1H), 7.64 (s, 1H), 7.59 (d, *J* = 8.4 Hz, 1H), 7.51 (dt, *J* = 10.7, 2.1 Hz, 1H), 7.20–7.17 (m, 2H), 5.20 (s, 2H), 4.22–4.18 (m, 1H), 3.68 (d, *J* = 14.0 Hz, 1H), 3.63 (d, *J* = 14.0 Hz, 1H), 2.73 (dd, *J* = 9.9, 6.0 Hz, 1H), 2.68 (q, *J* = 7.6 Hz, 1H), 2.52–2.47 (m, 1H), 2.42 (dd, *J* = 10.0, 3.5 Hz, 1H), 2.03–1.96 (m, 1H), 1.59–1.56 (m, 1H); ^13^C NMR (125 MHz, DMSO-*d*_6_): δ 163.4, 161.9 (d, *J* = 241.5 Hz), 157.7 (d, *J* = 241.7 Hz), 156.2 (d, *J* = 9.0 Hz), 154.7 (q, *J* = 37.1 Hz), 140.0 (d, *J* = 8.9 Hz), 138.1 (d, *J* = 11.3 Hz), 133.3, 125.1 (d, *J* = 3.1 Hz), 115.6 (q, 286.9 Hz), 115.3 (d, *J* = 2.4 Hz), 111.0 (d, *J* = 22.2 Hz), 107.4 (d, *J* = 26.2 Hz), 102.1 (d, *J* = 26.2 Hz), 97.9 (d, *J* = 20.1 Hz), 69.3, 69.0, 62.3, 52.3, 52.2, 34.3; *R*_*f*_ HPLC: 7.6 min (13 min from 10 to 95% MeCN in water (0.1% formic acid), then 7 min 95% MeCN). 96.8% purity; HRMS (ESI): *m*/*z* found, 527.0400 [M + H]^+^ (calcd C_20_H_18_BrF_6_N_2_O_3_^+^ 527.0400).

##### (*S*)-1-(5-Bromo-2-((2,6-difluoro-3-(2,2,2-trifluoroacetamido)­benzyl)­oxy)-4-fluorobenzyl)­pyrrolidine-3-carboxamide (**58f**)

The synthesis was performed according to GP6 using **57e/f** (100 mg, 0.219 mmol), (*S*)-pyrrolidine-3-carboxamide hydrochloride (132 mg, 0.876 mmol), acetic acid (75.3 μL, 1.31 mmol), and sodium cyanoborohydride (57.9 mg, 0.876 mmol) in DMF (10.0 mL). The crude was purified by preparative HPLC to yield 22.6 mg (19%) of the desired product **58f** as formate salt (Product decomposes with time). ^1^H NMR (500 MHz, DMSO-*d*_6_): δ 11.34 (br s, 1H), 8.13 (s, 1H), 7.62 (td, *J* = 8.8, 5.9 Hz, 1H), 7.58 (d, *J* = 8.4 Hz, 1H), 7.35 (d, *J* = 10.9 Hz, 1H), 7.28 (td, *J* = 9.0, 1.5 Hz, 1H), 7.22 (br s, 1H), 6.76 (br s, 1H), 5.21 (s, 2H), 3.50 (s, 2H), 2.81–2.71 (m, 2H), 2.58 (q, *J* = 8.8 Hz, 1H), 2.46–2.39 (m, 2H), 1.88–1.83 (m, 2H); ^13^C NMR (125 MHz, DMSO-*d*_6_): δ 175.5, 163.1, 162.3, 159.6 (dd, *J* = 248.6, 6.5 Hz), 157.6 (d, *J* = 242.0 Hz), 156.2 (d, *J* = 9.0 Hz), 155.4 (q, *J* = 37.0 Hz), 155.0 (dd, *J* = 252.7, 7.9 Hz), 133.3, 129.4 (d, *J* = 10.1 Hz), 125.3, 119.4 (dd, *J* = 13.4, 3.6 Hz), 115.8 (q, 286.5 Hz), 112.5 (dd, *J* = 20.0, 17.9 Hz), 111.8 (dd, *J* = 22.9, 3.4 Hz), 102.5 (d, *J* = 26.0, Hz), 98.5 (d, *J* = 20.9 Hz), 59.0, 56.7, 53.3, 51.3, 42.2, 27.5; ^19^F NMR (471 MHz, DMSO-*d*_6_): δ −73.9, −107.7, −114.8, −120.3; *R*_*f*_ HPLC: 7.6 min (13 min from 10 to 95% MeCN in water (0.1% formic acid), then 7 min 95% MeCN). 96.7% purity; HRMS (ESI): *m*/*z* found, 554.0506 [M + H]^+^ (calcd C_21_H_19_BrF_6_N_3_O_3_^+^ 554.0508).

##### (*S*)-1-(2-((1H-Benzo­[*d*]­imidazole-2-yl)­methoxy)-5-bromobenzyl)­pyrrolidin-3-ol (**58g**)

The synthesis was performed according to GP6 using **57g/h** (180 mg, 0.544 mmol), (*S*)-(−)-hydroxypyrrolidine (181 μL, 2.18 mmol), acetic acid (187 μL, 3.26 mmol), and sodium cyanoborohydride (144 mg, 2.18 mmol) in DMF (10.0 mL). The crude was purified by preparative HPLC to yield 82.3 mg (38%) of the desired product **58g** as a formate salt. ^1^H NMR (500 MHz, DMSO-*d*_6_): δ 8.16 (s, 1H), 7.57–7.55 (m, 2H), 7.51 (d, *J* = 2.6 Hz, 1H), 7.40 (dd, *J* = 8.7, 2.6 Hz, 1H), 7.19–7.15 (m, 3H), 5.41 (d, *J* = 1.1 Hz, 2H), 4.85 (br s, 1H), 4.29–4.24 (m, 1H), 3.72 (s, 2H), 2.78–2.72 (m, 2H), 2.56–2.50 (m, 2H), 2.08–1.99 (m, 1H), 1.67–1.61 (m, 1H); ^13^C NMR (125 MHz, DMSO-*d*_6_): δ 163.8, 155.6, 150.8, 138.9, 132.7, 131.2, 130.4, 122.4, 115.7, 115.4, 113.3, 69.9, 65.0, 62.9, 53.9, 52.8, 34.7; *R*_*f*_ HPLC: 7.3 min (13 min from 10 to 95% MeCN in water (0.1% formic acid), then 7 min 95% MeCN). 99.1% purity; HRMS (MALDI): *m*/*z* found, 402.0802 [M + H]^+^ (calcd C_19_H_21_BrN_3_O_2_^+^ 402.0812).

##### (*S*)-1-(2-((1*H*-Benzo­[*d*]­imidazole-2-yl)­methoxy)-5-bromobenzyl)­pyrrolidine-3-carboxamide (**58h**)

The synthesis was performed according to GP6 using **57g/h** (60.0 mg, 0.181 mmol), (*S*)-(−)-pyrrolidine-3-carboxamide hydrochloride (109 mg, 0.724 mmol), acetic acid (62.2 μL, 1.09 mmol), and sodium triacetoxyborohydride (192 mg, 0.724 mmol) in DMF (6.0 mL). The crude was purified by preparative HPLC to yield 15.0 mg (19%) of the desired product **58h** as a formate salt. ^1^H NMR (500 MHz, DMSO-*d*_6_): δ 8.15 (s, 1H), 7.58–7.55 (m, 3H), 7.47 (dd, *J* = 8.8, 2.6 Hz, 1H), 7.41 (br s, 1H), 7.21–7.18 (m, 3H), 6.93 (br s, 1H), 5.43 (s, 2H), 3.93 (s, 2H), 3.05 (t, *J* = 8.6 Hz, 1H), 3.00–2.94 (m, 1H), 2.92–2.84 (m, 2H), 2.79 (q, *J* = 7.8 Hz, 1H), 2.08–1.95 (m, 2H); ^13^C NMR (125 MHz, DMSO-*d*_6_): δ 174.8, 163.1, 155.3, 150.2, 138.4, 132.8, 131.6, 127.9, 122.0, 115.2, 115.0, 112.8, 64.4, 56.3, 53.6, 52.5, 42.0, 27.8; *R*_*f*_ HPLC: 6.2 min (13 min from 10 to 95% MeCN in water (0.1% formic acid), then 7 min 95% MeCN). 96.6% purity; HRMS (MALDI): *m*/*z* found, 451.0739 [M + Na]^+^ (calcd C_20_H_21_BrN_4_NaO_2_^+^ 451.0740).

##### (*S*)-1-(2-((1H-Benzo­[*d*]­imidazole-6-yl)­methoxy)-5-bromobenzyl)­pyrrolidin-3-ol (**58i**)

The synthesis was performed according to GP6 using **57i** (60.0 mg, 0.181 mmol), (*S*)-(−)-hydroxypyrrolidine (60.2 μL, 0.724 mmol), acetic acid (62.3 μL, 1.09 mmol), and sodium cyanoborohydride (47.9 mg, 0.724 mmol) in DMF (6.0 mL). The crude was purified by preparative HPLC to yield 43.3 mg (59%) of the desired product **58i**. ^1^H NMR (500 MHz, DMSO-*d*_6_): δ 8.23 (s, 1H), 7.69 (s, 1H), 7.59 (d, *J* = 8.3 Hz, 1H), 7.48 (d, *J* = 2.5 Hz, 1H), 7.36 (dd, *J* = 8.7, 2.6 Hz, 1H), 7.29 (dd, *J* = 8.3, 1.5 Hz, 1H), 7.06 (d, *J* = 8.8 Hz, 1H), 5.21 (s, 2H), 4.23–4.20 (m, 1H), 3.68 (d, *J* = 14.4 Hz, 1H), 3.63 (d, *J* = 14.4 Hz, 1H), 2.74–2.66 (m, 2H), 2.50–2.42 (m, 2H), 2.04–1.97 (m, 1H), 1.60–1.54 (m, 1H); ^13^C NMR (125 MHz, DMSO-*d*_6_): δ 155.4, 142.5, 138.1, 131.9, 130.5, 130.4, 130.3, 129.6, 121.7, 115.3, 114.7, 114.6, 112.0, 70.2, 69.4, 62.5, 52.7, 52.5, 34.4; *R*_*f*_ HPLC: 5.4 min (13 min from 10 to 95% MeCN in water (0.1% formic acid), then 7 min 95% MeCN). 99.6% purity; HRMS (MALDI): *m*/*z* found, 402.0799 [M + H]^+^ (calcd C_19_H_21_BrN_3_O_2_^+^ 402.0812).

##### (*S*)-1-(5-Bromo-2-(imidazo­[1,2-*a*]­pyridin-7-ylmethoxy)­benzyl)­pyrrolidin-3-ol (**58j**)

The synthesis was performed according to GP6 using **57j** (156 mg, 0.470 mmol), (*S*)-(−)-hydroxypyrrolidine (156 μL, 1.88 mmol), acetic acid (161 μL, 2.82 mmol), and sodium cyanoborohydride (124 mg, 1.28 mmol) in DMF (10.0 mL). The crude was purified by preparative HPLC to yield 137 mg (72%) of the desired product **58j** as a formate salt. ^1^H NMR (500 MHz, DMSO-*d*_6_): δ 8.54 (d, *J* = 6.9 Hz, 1H), 8.23 (br s, 1H), 7.93 (s, 1H), 7.67 (s, 1H), 7.57 (s, 1H), 7.53 (d, *J* = 2.4 Hz, 1H), 7.41 (dd, *J* = 8.8, 2.4 Hz, 1H), 7.06 (d, *J* = 8.8 Hz, 1H), 6.97 (d, *J* = 6.9 Hz, 1H), 6.18 (br s, 1H), 5.20 (s, 2H), 4.27–4.24 (m, 1H), 3.81 (d, *J* = 14.0 Hz, 1H), 3.77 (d, *J* = 14.0 Hz, 1H), 2.86–2.78 (m, 2H), 2.62 (q, *J* = 8.1 Hz, 1H), 2.56 (dd, *J* = 10.1, 2.8 Hz, 1H), 2.07–2.00 (m, 1H), 1.66–1.61 (m, 1H); ^13^C NMR (125 MHz, DMSO-*d*_6_): δ 163.9, 155.1, 144.3, 133.8, 133.3, 132.6, 130.9, 128.5, 126.9, 114.6, 113.2, 112.3, 111.5, 69.2, 68.6, 62.2, 52.7, 52.4, 34.2; *R*_*f*_ HPLC: 4.8 min (13 min from 10 to 95% MeCN in water (0.1% formic acid), then 7 min 95% MeCN). 97.9% purity; HRMS (MALDI): *m*/*z* found, 402.0814 [M + H]^+^ (calcd C_19_H_21_BrN_3_O_2_^+^ 402.0812).

#### Synthesis of Final Compounds **61** and **62**

##### *tert*-Butyl (2-(2-(2-((5-Bromo-2-((3-(2-chloro-4-(methylsulfonyl)­benzamido)-2,6-difluorobenzyl)­oxy)-4-fluorobenzyl)­amino)­ethoxy)­ethoxy)­ethyl)­carbamate (**59**)

The synthesis was performed according to GP6 using **11p** (57.7 mg, 0.10 mmol), BocNH-PEG2-CH_2_CH_2_NH_2_ (99.3 mg, 0.40 mmol), acetic acid (34.3 μL, 0.60 mmol), and sodium cyanoborohydride (25.1 mg, 0.40 mmol) in DMF (5.0 mL). The crude was purified by preparative HPLC to yield 68.5 mg (85%) of the desired product **59** as formate salt. ^1^H NMR (500 MHz, DMSO-*d*_6_): δ 10.64 (br s, 1H), 8.12 (s, 1H), 8.02 (dd, *J* = 8.0, 1.7 Hz, 1H), 7.96–7.96 (m, 1H), 7.88 (d, *J* = 8.0 Hz, 1H), 7.60 (d, *J* = 8.4 Hz, 1H), 7.34 (d *J* = 10.9 Hz, 1H), 7.26 (t, *J* = 8.8 Hz, 1H), 5.22 (s, 2H), 3.59 (s, 2H), 3.45–3.35 (m, 6H), 3.34 (s, 3H), 3.25–3.21 (m, 2H), 3.08–3.02 (m, 2H), 2.58 (t, *J* = 5.5 Hz, 2H), 1.35 (s, 9H). MS (ESI): *m*/*z* found, 810.10.

##### *N*-(3-((2-(((2-(2-(2-Aminoethoxy)­ethoxy)­ethyl)­amino)­methyl)-4-bromo-5-fluorophenoxy)­methyl)-2,4-difluorophenyl)-2-chloro-4-(methylsulfonyl)­benzamide (**60**)

Trifluoroacetic acid (1.0 mL) was added to a solution of **59** (22.0 mg, 0.0272 mmol) in DCM (2.0 mL) at 0 °C. The resulting mixture was stirred at 0 °C for 2 h. After that time, solvents were evaporated to obtain the crude product, which was purified by preparative HPLC. After HPLC purification, the fractions collected were basified with an aqueous sodium carbonate (1 M) and extracted with DCM 3× to avoid formylation of the free amine upon concentration with formic acid. Compound **60** was obtained as a free base with a yield of 19.4 mg (quantitative). ^1^H NMR (500 MHz, DMSO-*d*_6_): δ 10.54 (br s, 1H), 8.12 (s, 1H), 8.01 (dd, *J* = 8.0, 1.7 Hz, 1H), 7.94–7.90 (m, 1H), 7.88 (d, *J* = 8.0 Hz, 1H), 7.60 (d, *J* = 8.4 Hz, 1H), 7.34 (d *J* = 10.9 Hz, 1H), 7.26 (t, *J* = 8.8 Hz, 1H), 5.22 (s, 2H), 3.57 (s, 2H), 3.49–3.39 (m, 6H), 3.34 (s, 3H), 3.17 (m, 4H), 2.55 (t, *J* = 5.5 Hz, 2H); MS (ESI): *m*/*z* found, 709.70.

##### *N*-(3-((4-Bromo-2-(14-(5,5-difluoro-7-(1*H*-pyrrol-2-yl)-5*H*-5λ4,6λ4-dipyrrolo­[1,2-*c*:2′,1′-f]­[1,3,2]­diazaborinin-3-yl)-12-oxo-5,8-dioxa-2,11-diazatetradecyl)-5-fluorophenoxy)­methyl)-2,4-difluorophenyl)-2-chloro-4-(methylsulfonyl)­benzamide (**61**)

DIPEA (7.5 μL, 0.042 mmol) was added to a solution of **60** (10.2 mg, 0.0144 mmol) and Py-BODIPY-NHS ester (5.58 mg, 0.0144 mmol) in DMF (0.4 mL) at rt. The resulting mixture was stirred at rt for 2 h. The crude product was purified by preparative HPLC to yield 7.1 mg of **61** (43%) as a TFA salt. ^1^H NMR (500 MHz, DMSO-*d*_6_): δ 11.45 (s, 1H), 10.67 (s, 1H), 8.75 (s, 2H), 8.13 (d, *J* = 1.7 Hz, 1H), 8.04–7.96 (m, 2H), 7.92–7.85 (m, 2H), 7.81 (d, *J* = 8.0 Hz, 1H), 7.53 (d, *J* = 10.9 Hz, 1H), 7.43 (s, 1H), 7.39–7.36 (m, 1H), 7.34 (d, *J* = 4.5 Hz, 1H), 7.31–7.23 (m, 2H), 7.17 (d, *J* = 4.6 Hz, 1H), 7.00 (d, *J* = 3.9 Hz, 1H), 6.40–6.25 (m, 1H), 5.30 (s, 3H), 4.05 (t, *J* = 5.8 Hz, 2H), 3.60 (t, *J* = 5.0 Hz, 2H), 3.49 (s, 4H), 3.47 (s, 1H), 3.40 (t, *J* = 6.0 Hz, 2H), 3.34 (s, 3H), 3.22 (q, *J* = 5.9 Hz, 2H), 3.17–3.08 (m, 2H), 3.07–3.00 (m, 2H); ^13^C NMR (125 MHz, CD_2_Cl_2_): δ 170.5, 164.0, 161.5, 159.5, 155.8, 155.8, 150.3, 143.4, 140.0, 137.3, 136.0, 135.6, 132.3, 131.7, 131.6, 130.6, 129.2, 126.5, 126.13, 126.08, 126.0, 125.8, 123.53, 123.45, 120.34, 120.29, 119.3, 117.7, 116.5, 111.5, 111.3, 101.9, 70.3, 70.0, 69.9, 65.5, 59.8, 46.1, 45.8, 44.3, 39.3, 34.8, 24.4; ^19^F NMR (471 MHz, DMSO-*d*_6_): δ - 73.6, −103.6, −117.2, −121.3, −148.3; *R*_*f*_ HPLC: 3.9 min (2 min from 5 to 80% MeCN in water (0.1% formic acid), then 3 min from 80 to 95% MeCN, then 2 min 95% MeCN). 96.4% purity; MS (ESI): *m*/*z* found, 1021.15.

##### *N*-(3-((4-Bromo-5-fluoro-2-(12-oxo-16-((3*aS*,4*S*,6*aR*)-2-oxohexahydro-1*H*-thieno­[3,4-*d*]­imidazole-4-yl)-5,8-dioxa-2,11-diazahexadecyl)­phenoxy)­methyl)-2,4-difluorophenyl)-2-chloro-4-(methylsulfonyl)­benzamide (**62**)

Triethylamine (13.1 μL, 0.0925 mmol) was added to a solution of **60** (13.1 mg, 0.0185 mmol) and NHS-Biotin (6.32 mg, 0.0185 mmol) in DCM (3.0 mL) at 0 °C. The resulting mixture was stirred at rt for 2 h. After that time, the reaction mixture was diluted with a saturated aqueous sodium bicarbonate solution and extracted with ethyl acetate 3×. The combined organic layers were dried over magnesium sulfate, filtered, and evaporated. The crude product which was purified by preparative HPLC to yield of 4.3 mg of **62** (25%) as a formate salt. ^1^H NMR (500 MHz, DMSO-*d*_6_): δ 10.64 (br s, 1H), 8.17 (br s, 1H), 8.12 (d, *J* = 1.7 Hz, 1H), 8.00 (dd, *J* = 8.0, 1.7 Hz, 1H), 7.94–7.90 (m, 1H), 7.88 (d, *J* = 8.0 Hz, 1H), 7.80 (t, *J* = 5.7 Hz), 7.60 (d, *J* = 8.4 Hz, 1H), 7.34 (d *J* = 10.9 Hz, 1H), 7.26 (t, *J* = 8.8 Hz, 1H), 6.39 (s, 1H), 6.34 (s, 1H), 5.22 (s, 2H), 4.30–4.28 (m, 1H), 4.12–4.09 (m, 2H), 3.58 (s, 2H), 3.49–3.39 (m, 6H), 3.34 (s, 3H), 3.16 (q, *J* = 5.7 Hz, 2H), 3.09–3.05 (m, 2H), 2.80 (dd, *J* = 12.4, 5.1 Hz, 1H), 2.58–2.55 (m, 3H), 2.04 (t, *J* = 7.4 Hz, 2H), 1.63–1.41 (m, 4H), 1.31–1.24 (m, 2H); ^13^C NMR (125 MHz, DMSO-*d*_6_): δ 172.1, 164.3, 163.3, 158.2 (dd, *J* = 247.1, 6.6 Hz), 157.6 (d, *J* = 241.2 Hz), 156.2 (d, *J* = 9.0 Hz), 153.7 (dd, *J* = 251.6, 7.9 Hz), 143.1, 140.7, 132.3, 131.0, 130.0, 128.0, 127.2 (d, *J* = 9.4 Hz), 125.8, 125.3, 121.7 (dd, *J* = 12.8, 3.5 Hz), 112.2 (dd, *J* = 20.2, 17.6 Hz), 111.4 (dd, *J* = 22.4, 3.1 Hz), 102.4 (d, *J* = 26.0 Hz), 98.4 (d, *J* = 20.9 Hz), 69.9, 69.5, 69.1, 61.0, 59.2, 55.4, 47.8, 46.0, 43.1, 38.5, 35.1, 28.2, 25.2; ^19^F NMR (471 MHz, DMSO-*d*_6_): δ −108.3, −117.6, −122.1; *R*_*f*_ HPLC: 7.8 min (13 min from 10 to 95% MeCN in water (0.1% formic acid), then 7 min 95% MeCN). 97.9% purity; HRMS (MALDI): *m*/*z* found, 934.1573 [M + H]^+^ (calcd C_38_H_45_BrClF_3_N_5_O_8_S_2_^+^ 934.1528).

### Aqueous Solubility

Aqueous solubility was evaluated as previously described.^24^ Final concentrations of 42, 56, 75, 100, 134, 178, 237, 316, 422, 563, 750, and 1000 μM of **42** were prepared in PBS pH 7.4 solution containing 1% DMSO, in a 96-well transparent flat-bottom microtiter plate. Precipitation of the compound was measured at 600 and 800 nm after 1 and 24 h at room temperature using a microplate reader (Infinite M200, Tecan Group Ltd., Crailsheim, Germany), and solution clarity was confirmed by eye.

### Metabolic Stability

Compounds were tested according to the following method: a solution of the compound (final concentration 1 mM) was prepared in 100% DMSO. A 432 μL phosphate buffer (0.1 M, pH 7.4) together with 50 μL NADPH-regenerating system (30 mM glucose-6-phosphate, 4 U/mL glucose-6-phosphate dehydrogenase, 10 mM NADP, 30 mM MgCl_2_) and 5 μL of the corresponding test compound were preincubated at 37 °C. After 5 min, the reaction was started by the addition of 13 μL microsome mix from the liver of Sprague–Dawley rats (Thermo Fisher Scientific, Darmstadt, Germany; 20 mg protein/ml in 0.1 M phosphate buffer). The incubation was performed in a shaking water bath at 37 °C. The reaction was stopped by the addition of 500 μL ice-cold methanol at 0, 30, and 60 min. The samples were centrifuged at 5000*g* for 5 min at 4 °C. The supernatants were analyzed and quantified by HPLC. Control samples were always performed to check the stability of the compounds in the reaction mixture. The first control was without NADPH, which is needed for the enzymatic activity of the microsomes. The second control was with inactivated (microsomes, which had been incubated for 20 min at 90 °C). The third control was without test compounds (to determine the baseline). The amounts of the test compounds were quantified by an external calibration curve. The *in vitro* intrinsic clearance is calculated by eq 1, wherein k represents the gradient of the ln peak area ratio plotted against time.

$$Intrinsic\;clearance\;\left({CL}_{\mathrm{int}}\right)\left[\frac{\mu L}{\min\cdot mg}\right]=\frac{\left(\frac{incubation\;volume\;\left[\mu L\right]}{protein\;in\;the\;incubation\;\left[mg\right]}\right)}{\frac{1}{k}\;\left[\min\right]}$$ 1

### Recombinant Protein Expression and Purification

Constructs used in this study comprise the catalytic domains of USP25 or USP28, with deletion in their central UCID (amino acids 157-464-GSGG-538-706 or 149-458-GSGG-529-707, respectively), as previously described.^33^ The USP25 and USP28 variants were cloned between the 3C-cleavage and BamH1 restriction site of the vectors pCDF-14 or pCDF22 with an *N*-terminal 6xHis, or a thioredoxin-6xHis-tag, respectively, using the SLIC method^100^ and are controlled by IPTG inducible T7 expression cassettes (EMBL-plasmid-collection).^33^ For X-ray crystallography, a further truncated USP28cat ΔUCID construct was used (amino acids 149-399-GSGSGS-580-698), as previously described.^33^

The catalytic domains of USP25 and USP28 were expressed and purified as described previously.^33^ The elution fractions were analyzed by SDS-PAGE, and fractions containing the pure protein were concentrated and flash-frozen in liquid nitrogen prior to storage at −80 °C.

### Protein Crystallization and Inhibitor Soaking

USP28cat ΔUCID crystals were produced in a sitting drop, vapor diffusion plate, by mixing 1 μL protein (10 mg/mL) with 0.5 μL precipitant solution (0.2 M lithium sulfate, 0.05 M sodium chloride, 0.1 M 2-(*N*-morpholino)­ethanesulfonic acid (MES), pH 6.4, 14% (w/v) PEG 6000). Plates were incubated at 4 °C, and crystals appeared within 2 days. Inhibitor soaking was performed as previously described—crystals were transferred to a 1 μL drop of precipitant solution and supplemented with T-10531 inhibitor (10 mM stock in 100% DMSO) to a final concentration of 150 μM (final DMSO concentration 1.5%).^33^ After soaking for 3 h, crystals were transferred briefly to a cryoprotectant solution (0.2 M lithium sulfate, 0.05 M sodium chloride, 0.1 M MES pH 6.4, 14% (w/v) PEG 6000, 25% (v/v) ethylene glycol, 150 μM T-10531), before cooling in liquid nitrogen within nylon loops.

### X-ray Crystallography

Diffraction data were collected at EMBL beamline P13.^10^ The crystals crystallized in the cubic space group H 4_1_ 3 2. The data were integrated using XDS^39,41^ and scaled with AIMLESS (CCP4 suite).^40^ The structure was solved by molecular replacement, using Phaser-MR (Phenix)^101^ with USP28cat ΔUCID apo (PDB-ID: 8P19)^34^ as a search model. This was followed by successive rounds of refinement of coordinates, *B*-factors, TLS-parameters, and occupancies using Phenix-Refine^101^ and manual model building using Coot.^42,44^ Inhibitor coordinate restraints were generated with the GRADE server (Smart O. S., Womack T. O., Sharff A., Flensburg C., Keller P., Paciorek C., Vonrhein C., Bricogne G. (2011) grade, version 1.101, Global Phasing Ltd., Cambridge, UK). Data collection and refinement statistics are found in Table S1. A schematic of the ligand interface was generated using LIGPLOT,^43^ and structural figures were produced using PyMOL (The PyMOL Molecular Graphics System, Version 3.1.3.1 Schrödinger, LLC).

The model of **T-10531** bound to USP28 and associated crystallographic data have been deposited in the Protein Data Bank under the accession code PDB 9SUU.

### Dose-Response Assays

The inhibitory potencies of the synthesized compounds were measured utilizing the fluorogenic substrate, Ub-Rhodamine110Gly (Ub-Rho110, UbiQ Bio). All compounds were sent as lyophilized powder and resuspended in 100% DMSO to obtain 50 mM stocks and 10 mM aliquots. Next, utilizing the assay buffer [20 mM 4-(2-hydroxyethyl)-1-piperazineethanesulfonic acid (HEPES) pH 7.5, 150 mM NaCl, 1 mM tris­(2-carboxyethyl)­phosphine (TCEP), and 50 μg/mL BSA], a 3-fold, ten-point dilution series, starting from 100 μM inhibitor was prepared, and 20 nM USP25 or USP28, respectively, were incubated with the diluted compounds for 15 min at room temperature. The mixture was transferred to a black nonbinding 384-well plate (Greiner 781900). The cleavage reaction was initiated by the addition of Ub-Rho110 (final concentration: 250 nM) to the DUB-inhibitor mixture, and the fluorescence was measured using the CLARIOstar microplate reader (BMG Labtech) at 25 °C (λ_ex/em_ 485/535) for 30 min. The initial slope of the increasing fluorescence signal was measured, and the relative inhibition was determined and plotted by nonlinear regression against the inhibitor concentration utilizing the GraphPad Prism (version 10.4.1) software. All statistical results are presented as the mean ± SD and based on technical replicates of two batches from independently purified proteins (*n* ≥ 3).

### Cellular Target Engagement

The assay was performed as described previously.^45^ In brief, full-length USP25, USP25, USP25 P535L, and USP28 were obtained as plasmids cloned in frame with a terminal NanoLuc-fusion. Plasmids were transfected into HEK293T cells using FuGENE HD (Promega, E2312), and proteins were allowed to express for 20 h. Serially diluted inhibitor and Tracer (compound **60**) (TracerDB ID: T000062) at the Tracer KD concentration taken from TracerDB (tracerdb.org)^45^ were pipetted into white 384-well plates (Greiner 781207) using an ECHO acoustic dispenser (Labcyte). The corresponding protein-transfected cells were added and reseeded at a density of 2 × 10^5^ cells/mL after trypsinization and resuspending in Opti-MEM without phenol red (Life Technologies). The system was allowed to equilibrate for 2 h at 37 °C/5% CO_2_ prior to BRET measurements. To measure BRET, NanoBRET NanoGlo Substrate was added as per the manufacturer’s protocol, and filtered luminescence was measured on a PHERAstar plate reader (BMG Labtech) equipped with a luminescence filter pair (450 nm BP filter (donor) and 610 nm LP filter (acceptor)). Competitive displacement data were then graphed using GraphPad Prism 9 software using a normalized 3-parameter curve fit with the following equation: *Y* = 100/(1 + 10­(*X* – log IC_50_)).

### Cell Lines and Cell Culture

HeLa cells were originally obtained from the American Type Culture Collection. Cells were cultured in Dulbecco’s modified Eagle’s medium (DMEM) supplemented with 10% fetal bovine serum (FBS).

### DUB Inhibitor Competition Assay in Cells

Cells were incubated with indicated concentrations of the inhibitors for indicated time points. Cells were lysed in lysis buffer (50 mM Tris, 150 mM NaCl, 0.5% Triton X-100, and 2 mM TCEP at pH 7.5, supplemented with protease inhibitor cocktail (Roche Diagnostics, Mannheim, Germany)). The lysates were sonicated using 5 cycles of 30 s pulse on, 30 s pulse off on Biorupter Pico (Diagenode). The total lysates were centrifuged at maximum speed for 15 min at 4 °C, and the supernatants were transferred to fresh Eppendorf tubes. The protein concentrations of the resulting lysates were determined using the Pierce bicinchoninic acid assay kit (Thermo Scientific). Equal amounts of protein were used for each condition and incubated with the Rho-K­(Biotin)-Ub-PA probe at a final concentration of 0.5 or 1 μM at 37 °C for 5 or 10 min. The samples were immediately boiled in NuPAGE LDS sample buffer (3×) to stop the enzyme probe reaction. They were separated by SDS-PAGE using a 4–12% Bis-Tris gel with 3-(*N*-morpholino)­propanesulfonic acid (MOPS) SDS running buffer and visualized by fluorescence scanning on a Typhoon FLA 9500 using a Rhodamine channel (λ_ex/em_ 473/530 nm).

### DUB Inhibitor Competition Assay in Cell Lysate

Cell lysates were prepared as explained above. Lysates were incubated with indicated concentrations of the inhibitors for 5 min at 37 °C. Subsequently, the lysates were incubated with the Rho-K­(Biotin)-Ub-PA probe at a final concentration of 1 μM at 37 °C for 10 min.

### Immunoblot Analysis

The fluorescence scan of the SDS-PAGE was followed by protein transfer onto nitrocellulose membranes. The membranes were incubated for 1 h in blocking buffer [5% nonfat dry milk in phosphate buffer containing Tween-20­(PBST)]. The membranes were washed with PBST. Subsequently, they were incubated with the following primary antibodies for 1 h at room temperature: anti-USP25 (1:1000, Abcam, #ab187156), anti-USP28 (1:1000, Abcam, #ab126604), and anti-αTubulin (1:1000, Proteintech, #11224-1-AP). After three washes for 10 min with PBST, the membranes were incubated for 1 h at room temperature with horseradish peroxidase (HRP)-conjugated secondary antibodies. The membranes were then again washed three times with PBST and visualized with SuperSignal West Pico Chemiluminescent Substrate (Thermo Fisher Scientific).

### Transfection

For siRNA transfection, siRNA targeting USP28 (siGENOME, M-006076-01-0005 5 nmol) was obtained from Dharmacon. The knockdown of USP28 in HeLa cells was performed in a 24 well plate format. 50 μL of siRNA (500 nM stock) was incubated with 1 μL DharmaFECT 1 (Dharmacon) diluted in 50 μL medium without supplements by shaking for 30 min at room temperature. Cells were added to the transfection mixture and cultured at 37 °C and 5% CO_2_ for 72 h. For additional treatment with the inhibitor, old medium was replaced with fresh medium, and the inhibitor was added at indicated concentrations and incubated for 4 h at 37 °C and 5% CO_2_. Cells were harvested and analyzed as described above.

### CellTiter-Glo Assay

Cell viability was assessed using the CellTiter-Glo assay (Promega) according to the manufacturer’s guidelines, with slight modifications. Hep-G2 cells were detached from Collagen-G-coated tissue culture flasks by Trypsin treatment and resuspended in white DMEM high-glucose medium (Gibco #31053) containing 10% FBS, 100 units/mL Penicillin, 100 μg/mL Streptomycin, and 2 mM l-Glutamine (Gibco). The cell suspension was filtered through a 40 μm cell strainer (Pluriselect #43-50040) to remove aggregates, and the cell density was adjusted to 100,000 cells/mL. Then, 30 μL/well of the suspension (containing 3000 cells) was seeded into 96 well half-area white polystyrene flat-bottom tissue culture plates (Greiner #675083). Compounds were added in 10 μL of medium with 2% DMSO to give the indicated compound concentrations and 0.5% DMSO during treatment at 37 °C and 5% CO_2_. For detection, 20 μL of CellTiter-Glo reagent mix was added to each well, and the plates were incubated for 30 min at room temperature in the dark before luminescence was measured using the standard attenuation protocol on a Tecan SPARK. Control wells containing only medium and wells with cells treated with 0.5% DMSO served as 0% and 100% viability control, respectively. Paclitaxel (50 μM) was used as a positive control to assess assay performance.

### Thermal Shift

Thermal melting curves were measured on a Stratagene Mx3005P (Agilent Technologies) in a range from 25 to 77 °C. Twenty microliter of protein solution (20 mM HEPES, 150 mM NaCl, 1 mM TCEP, pH 8.0) with Sypro Orange was mixed with 1 μL compound (10 mM in DMSO) and analyzed in a 96 well plate. The final sample contained 5 μM protein, 0.01% Triton-X-100, 5× Sypro Orange, and 500 μM compound. For Sypro Orange measurement, the excitation wavelength was set to 465 nm and the emission wavelength to 590 nm. All samples were determined in triplicate. As a reference, ligand-free protein samples were analyzed, which received only pure DMSO. First, the data were analyzed in Excel and then subjected to a Boltzmann fit in GraphPad Prism.^46^

### ITC

The capability of the USP25 and USP28 proteins to bind the compound **42 (T-10531)** was verified via ITC conducted on a TA Instruments Affinity ITC (TA Instruments, New Castle, Delaware, USA). Recombinant USP25 (50 μM) and USP28 (50 μM) and compounds **33** (**T-10507**) and **42** (**T-10531**) (10.6 μM) were dissolved in HEPES buffer [20 mM HEPES pH 8.0, 150 mM NaCl, 1 mM TCEP] supplemented with 0.1% DMSO (v/v). The ITC instrument was adjusted to 21 °C, and the stirring rate was set to 75 rpm. A compound solution was filled into the reaction cell (178 μL cell volume), and the protein solution was titrated (inverse titration). For **42** (**T-10531**), 30 injections of 2.5 μL of USP25 and 20 injections of 1.5 μL of USP28 each, for **33** (**T-10507**), 15 injections of 2.5 μL of USP25 and 20 injections of 1.5 μL of USP28 each were carried out, whereby the first injection was performed with a reduced volume of 0.5 μL. The heats of dilution resulting from titrating one of the USP25 or USP28 proteins into the cell containing only buffer were recorded separately and subtracted from the raw ITC data obtained with the compound. Data were analyzed using the NanoAnalyze software package (version 3.7.5). In order to fit the binding affinity constant (*K*_d_), an independent binding model was used, from which the constant blank has been subtracted.

## Supplementary Material





## Notes

Synthesis methods and characterization of starting materials; HPLC traces of **T-10531** and **T-10507**; X-ray data collection and refinement statistics; detail of **T-10531** binding site and X-ray density; comparison of **T-10531**, vismodegib, and AZ1 binding to USP28; and correlation of clogP and the inhibitory potency (PDF)
Molecular Formula Strings (CSV)

## Abbreviations

ABPP: activity-based protein profiling
ATCC: American type culture collection
BRET: bioluminescence resonance energy transfer
DMEM: Dulbecco’s modified Eagle medium
DSF: differential scanning fluorimetry
DUBs: deubiquitylases
ERAD: endoplasmic-reticulum-associated protein degradation
FBS: fetal bovine serum
HEPES: 4-(2-hydroxyethyl)-1-piperazineethanesulfonic acid
HRP: horseradish peroxidase
ITC: isothermal titration calorimetry
MES: 2-(*N*-morpholino)­ethanesulfonic acid
MOPS: 3-(*N*-morpholino)­propanesulfonic acid
PBST: phosphate buffer containing Tween-20
SCC: squamous cell carcinoma
TCEP: tris­(2-carboxyethyl)­phosphine
TNBC: triple-negative breast cancer
Ub: ubiquitin
USPs: ubiquitin-specific proteases.

## References

[1] Snyder N. A., Silva G. M. (2021). Deubiquitinating Enzymes (DUBs): Regulation, Homeostasis,
and Oxidative Stress Response. J. Biol. Chem..

[2] Lange S. M., Armstrong L. A., Kulathu Y. (2022). Deubiquitinases: From Mechanisms
to Their Inhibition by Small Molecules. Mol.
Cell.

[3] Haq S., Ramakrishna S. (2017). Deubiquitylation
of Deubiquitylases. Open Biol..

[4] Swatek K. N., Komander D. (2016). Ubiquitin Modifications. Cell
Res..

[5] Clague M. J., Urbé S., Komander D. (2019). Breaking the Chains: Deubiquitylating
Enzyme Specificity Begets Function. Nat. Rev.
Mol. Cell Biol..

[6] Deng L., Meng T., Chen L., Wei W., Wang P. (2020). The Role of
Ubiquitination in Tumorigenesis and Targeted Drug Discovery. Signal Transduct. Target. Ther..

[7] Li Y., Reverter D. (2021). Molecular Mechanisms of DUBs Regulation in Signaling
and Disease. Int. J. Mol. Sci..

[8] Li K.-Q., Bai X., Ke A.-T., Ding S.-Q., Zhang C.-D., Dai D.-Q. (2024). Ubiquitin-Specific
Proteases: From Biological Functions to Potential Therapeutic Applications
in Gastric Cancer. Biomed. Pharmacother..

[9] Ye Y., Scheel H., Hofmann K., Komander D. (2009). Dissection of USP Catalytic
Domains Reveals Five Common Insertion Points. Mol. Biosyst..

[10] Cianci M., Bourenkov G., Pompidor G., Karpics I., Kallio J., Bento I., Roessle M., Cipriani F., Fiedler S., Schneider T. R. (2017). P13, the
EMBL Macromolecular Crystallography Beamline
at the Low-Emittance PETRA III Ring for High- and Low-Energy Phasing
with Variable Beam Focusing. J. Synchrotron
Radiat..

[11] Sauer F., Klemm T., Kollampally R. B., Tessmer I., Nair R. K., Popov N., Kisker C. (2019). Differential
Oligomerization of the
Deubiquitinases USP25 and USP28 Regulates Their Activities. Mol. Cell.

[12] Wu Y., Wang Y., Yang X. H., Kang T., Zhao Y., Wang C., Evers B. M., Zhou B. P. (2013). The Deubiquitinase
USP28 Stabilizes LSD1 and Confers Stem-Cell-like Traits to Breast
Cancer Cells. Cell Rep..

[13] Zhao L.-J., Zhang T., Feng X.-J., Chang J., Suo F.-Z., Ma J.-L., Liu Y.-J., Liu Y., Zheng Y.-C., Liu H.-M. (2019). USP28 Contributes to the Proliferation
and Metastasis
of Gastric Cancer. J. Cell. Biochem..

[14] Knobel P. A., Belotserkovskaya R., Galanty Y., Schmidt C. K., Jackson S. P., Stracker T. H. (2014). USP28 Is
Recruited to Sites of DNA Damage by the Tandem
BRCT Domains of 53BP1 but Plays a Minor Role in Double-Strand Break
Metabolism. Mol. Cell. Biol..

[15] Wang J., Dong Y., Ma H., Wu L., Zhen X., Tang L., Jin J., Han S., Zhang P., Peng J. (2022). The Deubiquitinase USP28 Stabilizes
the Expression of RecQ Family
Helicases and Maintains the Viability of Triple Negative Breast Cancer
Cells. J. Biol. Chem..

[16] Müller I., Strozyk E., Schindler S., Beissert S., Oo H. Z., Sauter T., Lucarelli P., Raeth S., Hausser A., Al Nakouzi N., Fazli L., Gleave M. E., Liu H., Simon H.-U., Walczak H., Green D. R., Bartek J., Daugaard M., Kulms D. (2020). Cancer Cells Employ Nuclear Caspase-8
to Overcome the P53-Dependent G2/M Checkpoint through Cleavage of
USP28. Mol. Cell.

[17] Prieto-Garcia C., Tomašković I., Shah V. J., Dikic I., Diefenbacher M. (2021). USP28: Oncogene or Tumor Suppressor?
A Unifying Paradigm
for Squamous Cell Carcinoma. Cells.

[18] Xie S., Liu S., Zhang T., Shi W., Xing Y., Fang W., Zhang M., Chen M.-Y., Xu S., Fan M., Li L., Zhang H., Zhao N., Zeng Z., Chen S., Zeng X., Deng W., Tang Q. (2024). USP28 Serves as a Key
Suppressor of Mitochondrial Morphofunctional Defects and Cardiac Dysfunction
in the Diabetic Heart. Circulation.

[19] Xu D., Liu J., Fu T., Shan B., Qian L., Pan L., Yuan J. (2017). USP25 Regulates
Wnt Signaling by Controlling the Stability of Tankyrases. Genes Dev..

[20] Nelson J. K., Thin M. Z., Evan T., Howell S., Wu M., Almeida B., Legrave N., Koenis D. S., Koifman G., Sugimoto Y., Llorian Sopena M., MacRae J., Nye E., Howell M., Snijders A. P., Prachalias A., Zen Y., Sarker D., Behrens A. (2022). USP25 Promotes Pathological HIF-1-Driven
Metabolic Reprogramming and Is a Potential Therapeutic Target in Pancreatic
Cancer. Nat. Commun..

[21] Habtemichael E. N., Li D. T., Alcázar-Román A., Westergaard X. O., Li M., Petersen M. C., Li H., DeVries S. G., Li E., Julca-Zevallos O., Wolenski J. S., Bogan J. S. (2018). Usp25m Protease Regulates Ubiquitin-like
Processing of TUG Proteins to Control GLUT4 Glucose Transporter Translocation
in Adipocytes. J. Biol. Chem..

[22] Zhong B., Liu X., Wang X., Chang S. H., Liu X., Wang A., Reynolds J. M., Dong C. (2012). Negative Regulation of IL-17-Mediated
Signaling and Inflammation by the Ubiquitin-Specific Protease USP25. Nat. Immunol..

[23] Lin D., Zhang M., Zhang M.-X., Ren Y., Jin J., Zhao Q., Pan Z., Wu M., Shu H.-B., Dong C., Zhong B. (2015). Induction of USP25 by Viral Infection
Promotes Innate Antiviral Responses by Mediating the Stabilization
of TRAF3 and TRAF6. Proc. Natl. Acad. Sci. U.S.A..

[24] Blount J. R., Burr A. A., Denuc A., Marfany G., Todi S. V. (2012). Ubiquitin-Specific
Protease 25 Functions in Endoplasmic Reticulum-Associated Degradation. PLoS One.

[25] Zhang H. (2024). Targeting
USP25 in the Heart. JACC Basic Transl. Sci..

[26] Zheng Q., Song B., Li G., Cai F., Wu M., Zhao Y., Jiang L., Guo T., Shen M., Hou H., Zhou Y., Zhao Y., Di A., Zhang L., Zeng F., Zhang X.-F., Luo H., Zhang X., Zhang H., Zeng Z., Huang T. Y., Dong C., Qing H., Zhang Y., Zhang Q., Wang X., Wu Y., Xu H., Song W., Wang X. (2022). USP25 Inhibition Ameliorates
Alzheimer’s Pathology through the Regulation of APP Processing
and Aβ Generation. J. Clin. Invest..

[27] Liu Z., Zhao T., Li Z., Sun K., Fu Y., Cheng T., Guo J., Yu B., Shi X., Liu H. (2020). Discovery of [1,2,3]­Triazolo­[4,5-d]­Pyrimidine Derivatives
as Highly
Potent, Selective, and Cellularly Active USP28 Inhibitors. Acta Pharm. Sin. B.

[28] Varca A. C., Casalena D., Chan W. C., Hu B., Magin R. S., Roberts R. M., Liu X., Zhu H., Seo H.-S., Dhe-Paganon S., Marto J. A., Auld D., Buhrlage S. J. (2021). Identification
and Validation of Selective Deubiquitinase Inhibitors. Cell Chem. Biol..

[29] Ruiz E. J., Pinto-Fernandez A., Turnbull A. P., Lan L., Charlton T. M., Scott H. C., Damianou A., Vere G., Riising E. M., Da Costa C., Krajewski W. W., Guerin D., Kearns J. D., Ioannidis S., Katz M., McKinnon C., O’Connell J., Moncaut N., Rosewell I., Nye E., Jones N., Heride C., Gersch M., Wu M., Dinsmore C. J., Hammonds T. R., Kim S., Komander D., Urbe S., Clague M. J., Kessler B. M., Behrens A. (2021). USP28 Deletion and
Small-Molecule Inhibition Destabilizes c-MYC and Elicits Regression
of Squamous Cell Lung Carcinoma. eLife.

[30] Xu Z., Wang H., Meng Q., Ding Y., Zhu M., Zhou H., Zhang N., Shi L. (2023). Otilonium Bromide Acts
as a Selective USP28 Inhibitor and Exhibits Cytotoxic Activity against
Multiple Human Cancer Cell Lines. Biochem. Pharmacol..

[31] Wrigley J. D., Gavory G., Simpson I., Preston M., Plant H., Bradley J., Goeppert A. U., Rozycka E., Davies G., Walsh J., Valentine A., McClelland K., Odrzywol K. E., Renshaw J., Boros J., Tart J., Leach L., Nowak T., Ward R. A., Harrison T., Andrews D. M. (2017). Identification and Characterization
of Dual Inhibitors
of the USP25/28 Deubiquitinating Enzyme Subfamily. ACS Chem. Biol..

[32] Wang H., Meng Q., Ding Y., Xiong M., Zhu M., Yang Y., Su H., Gu L., Xu Y., Shi L., Zhou H., Zhang N. (2021). USP28 and USP25 Are Downregulated
by Vismodegib in Vitro and in Colorectal Cancer Cell Lines. FEBS J..

[33] Patzke J. V., Sauer F., Nair R. K., Endres E., Proschak E., Hernandez-Olmos V., Sotriffer C., Kisker C. (2024). Structural Basis for
the Bi-Specificity of USP25 and USP28 Inhibitors. EMBO Rep..

[34] Zhou D., Xu Z., Huang Y., Wang H., Zhu X., Zhang W., Song W., Gao T., Liu T., Wang M., Shi L., Zhang N., Xiong B. (2023). Structure-Based Discovery of Potent
USP28 Inhibitors Derived from Vismodegib. Eur.
J. Med. Chem..

[35] Guerin, D. J. ; Ng, P. Y. ; Wang, Z. ; Shelekhin, T. ; Caravella, J. ; Zablocki, M.-M. ; Downing, J. R. ; Li, H.. ; Ioannidis, S.. Carboxamides as Ubiquitin-Specific Protease Inhibitors. WO 2020033707 A1, 2019

[36] Gersch M., Wagstaff J. L., Toms A. V., Graves B., Freund S. M. V., Komander D. (2019). Distinct USP25 and
USP28 Oligomerization States Regulate
Deubiquitinating Activity. Mol. Cell.

[37] Ekkebus R., van Kasteren S. I., Kulathu Y., Scholten A., Berlin I., Geurink P. P., de Jong A., Goerdayal S., Neefjes J., Heck A. J. R., Komander D., Ovaa H. (2013). On Terminal
Alkynes That Can React with Active-Site Cysteine Nucleophiles in Proteases. J. Am. Chem. Soc..

[38] Kooij R., Liu S., Sapmaz A., Xin B.-T., Janssen G. M. C., Van
Veelen P. A., Ovaa H., Dijke P. T., Geurink P. P. (2020). Small-Molecule
Activity-Based Probe for Monitoring Ubiquitin C-Terminal Hydrolase
L1 (UCHL1) Activity in Live Cells and Zebrafish Embryos. J. Am. Chem. Soc..

[39] Kabsch W. (2010). XDS. Acta Crystallogr. D Biol. Crystallogr..

[100] Li M. Z., Elledge S. J. (2007). Harnessing homologous
recombination
in vitro to generate recombinant DNA via SLIC. Nat Methods.

[40] Winn M. D., Ballard C. C., Cowtan K. D., Dodson E. J., Emsley P., Evans P. R., Keegan R. M., Krissinel E. B., Leslie A. G. W., McCoy A., McNicholas S. J., Murshudov G. N., Pannu N. S., Potterton E. A., Powell H. R., Read R. J., Vagin A., Wilson K. S. (2011). Overview
of the *CCP* 4 Suite and Current Developments. Acta Crystallogr. D Biol. Crystallogr..

[41] Pearce N. M., Krojer T., Bradley A. R., Collins P., Nowak R. P., Talon R., Marsden B. D., Kelm S., Shi J., Deane C. M., Von Delft F. (2017). A Multi-Crystal
Method for Extracting
Obscured Crystallographic States from Conventionally Uninterpretable
Electron Density. Nat. Commun..

[42] Emsley P., Lohkamp B., Scott W. G., Cowtan K. (2010). Features and Development
of Coot. Acta Crystallogr. D Biol. Crystallogr..

[101] Liebschner D., Afonine P. V., Baker M. L., Bunkóczi G., Chen V. B., Croll T. I., Hintze B., Hung L.-W., Jain S., McCoy A. J., Moriarty N. W., Oeffner R. D., Poon B. K., Prisant M. G., Read R. J., Richardson J. S., Richardson D. C., Sammito M. D., Sobolev O. V., Stockwell D. H., Terwilliger T. C., Urzhumtsev A. G., Videau L. L., Williams C. J., Adams P. D. (2019). Macromolecular structure
determination using X-rays,
neutrons and electrons: recent developments in Phenix. Acta Crystallogr D Struct Biol.

[43] Wallace A. C., Laskowski R. A., Thornton J. M. (1995). LIGPLOT: A Program to Generate Schematic
Diagrams of Protein-Ligand Interactions. Protein
Eng..

[44] Schwalm M. P., Saxena K., Müller S., Knapp S. (2024). Luciferase- and HaloTag-Based
Reporter Assays to Measure Small-Molecule-Induced Degradation Pathway
in Living Cells. Nat. Protoc..

[45] Dopfer J., Vasta J. D., Müller S., Knapp S., Robers M. B., Schwalm M. P. (2024). tracerDB: A Crowdsourced
Fluorescent Tracer Database
for Target Engagement Analysis. Nat. Commun..

[46] Niesen F. H., Berglund H., Vedadi M. (2007). The Use of
Differential Scanning
Fluorimetry to Detect Ligand Interactions That Promote Protein Stability. Nat. Protoc..

