# Supplementary material for: Structure Merging Approach Leads to New Dual Potent and Selective USP25/USP28 Inhibitors
Source: J Med Chem. 2026 Apr 22;69(9):10140–68. doi: 10.1021/acs.jmedchem.5c03045 (PMC13181796; doi:10.1021/acs.jmedchem.5c03045)
Supplement: Supplementary file 1 [file jm5c03045_si_001.pdf]

Supporting Information

Structure Merging Approach Leads to New Dual Potent and Selective USP25/USP28 Inhibitors

*Victor Hernandez-Olmos[1,2], Jonathan Vincent Patzke[3], Caroline E. Stone[3], Radhika Karal Nair[3], Kathrin Weller[4], Florian Sauer[3], Erik Endres[5], Oumaima Jamai[6], Lea Rachor[6], Cornelia H. Warmutz[6], Martin P. Schwalm[6,7], Marko Mitrovic,[6,7] Vincent Grenier[6,7], Johanna H. M. Ehrler[6], Anna Proschak[6], Jan Heering[1,2], Christoph Sotriffer[5], Monique P. C. Mulder[4], Stefan Knapp[6,7], Caroline Kisker[3], Ewgenij Proschak[1,2,6]**

[1] Fraunhofer Institute for Translational Medicine and Pharmacology ITMP, Theodor-Stern-Kai 7, 60596 Frankfurt am Main, Germany

[2] Fraunhofer Cluster of Excellence Immune-Mediated Diseases CIMD, Theodor-Stern-Kai 7,
60596 Frankfurt am Main, Germany

[3] Rudolf Virchow Center for Integrative and Translational Bioimaging, Institute for Structural Biology, Julius-Maximilians-University Würzburg, 97080 Würzburg, Germany

[4] Department of Cell and Chemical Biology, Leiden University Medical Centre, Einthovenweg 20, 2300 RC Leiden, The Netherlands

[5] Institute of Pharmacy and Food Chemistry, Julius-Maximilians-University Würzburg, 97074 Würzburg, Germany

[6] Institute of Pharmaceutical Chemistry, Goethe University Frankfurt, 60438 Frankfurt am Main, Germany

[7] Structural Genomics Consortium (SGC), Buchmann Institute for Molecular Life Sciences (BMLS), 60438 Frankfurt am Main, Germany.

Synthesis of starting materials	S3-S10
**SI Figure S1**: HPLC trace of compound **33** (**T-10507**).	S11
**SI Figure S2**: HPLC trace of compound **42** (**T-10531**).	S11
**SI Table S1**: X-ray data collection and refinement statistics	S12
**SI Figure S3**: Detail of **T-10531** binding site and X-ray density	S13
**SI Figure S4**: Comparison of **T-10531**, Vismodegib, and AZ1 binding to USP28	S13
**SI Figure S5**: Correlation of clogP and the inhibitory potency	S14

Preparation of starting materials

2-Chloro-4-(methylsulfonyl)benzoyl chloride (1a)

2-Chloro-4-(methylsulfonyl)benzoic acid (939 mg, 4 mmol) was dissolved in thionyl chloride (15.0 mL) at rt and then refluxed for 3h. The excess of thionyl chloride was removed at reduced preassure and dried overnight. The residue was used in the next step without further purification. Yield (1.01 g, quantitative). ^{1}H NMR (250 MHz, $CDCl_3$) δ 8.16 (d, *J* = 8.2 Hz, 1H), 8.08 (d, *J* = 1.7 Hz, 1H), 7.97 (dd, *J* = 8.2, 1.7 Hz, 1H), 3.11 (s, 3H).

(3-Fluoro-5-nitrophenyl)methanol

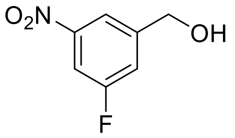

The synthesis was performed according to GP1 using 3-fluoro-5-nitrobenzoic acid (800 mg, 4.32 mmol, 1.0 eq), sodium borohydride (334 mg, 8.64 mmol, 2.0 eq), and boron trifluoride diethyl etherate (1.40 mL, 12.0 mmol, 2.7 eq) in 12 mL THF. The reaction mixture was stirred for 20 h at rt. The product was obtained as a yellow solid in a yield of 98% (723 mg). ^{1}H NMR (250 MHz, $CDCl_3$) δ 8.05 (s, 1H), 7.84 (m, 1H), 7.47 (d, *J* = 7.3 Hz, 1H), 4.83 (s, 2H).

(2,6-Difluoro-3-nitrophenyl)methanol

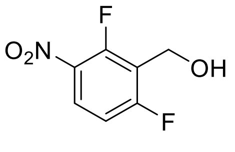

The synthesis was performed according to GP1 using 2,6-difluoro-3-nitrobenzoic acid (1.0 g, 4.92 mmol, 1.0 eq), sodium borohydride (380 mg, 9.84 mmol, 2.0 eq), and boron trifluoride diethyl etherate (1.60 mL, 13.0 mmol, 2.7 eq) in 12 mL THF. The reaction mixture was stirred for 20 h at rt.

The product was obtained as a brown solid in a yield of 87% (811 mg). ^{1}H NMR (250 MHz, $CDCl_3$) δ 8.15-8.06 (m, 1H), 7.10-7.02 (m, 1H), 4.85 (s, 2H).

(2,3-Difluoro-3-nitrophenyl)methanol

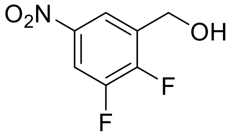

The synthesis was performed according to GP1 using 2,3-difluoro-3-nitrobenzoic acid (700 mg, 3.45 mmol, 1.0 eq), sodium borohydride (266 mg, 6.90 mmol, 2.0 eq), and boron trifluoride diethyl etherate (1.20 mL, 9.30 mmol, 2.7 eq) in 10 mL THF. The reaction mixture was stirred for 19 h at rt. The product was obtained as a brown solid in a yield of 93% (607 mg). ^{1}H NMR (250 MHz, $CDCl_3$) δ 8.27-8.24 (m, 1H, 8.06-8.99 (m, 1H), 4.89 (s, 2H).

(2,5-Difluoro-3-nitrophenyl)methanol

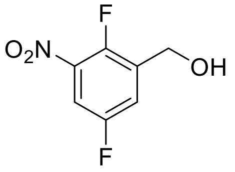

The synthesis was performed according to GP1 using 2,5-difluoro-3-nitrobenzoic acid (450 mg, 2.22 mmol, 1.0 eq), sodium borohydride (171 mg, 4.44 mmol, 2.0 eq), and boron trifluoride diethyl etherate (740 μL, 6.0 mmol, 2.7 eq) in 10 mL THF. The reaction mixture was stirred for 17 h at rt. The product was obtained as a brown solid in a yield of 99% (418 mg). ^{1}H NMR (250 MHz, $CDCl_3$) δ 7.72-7.65 (m, 1H), 7.62-7.55 (m, 1H), 4.87 (s, 2H).

(3-Amino-5-fluorophenyl)methanol (2d)

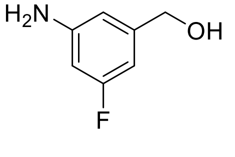

The synthesis was performed according to GP2 using (3-Fluoro-5-nitrophenyl)methanol (710 mg, 4.15 mmol, 1.0 eq) and tin(II) chloride dihydrate (3.82 g, 16.6 mmol, 4.0 eq) in 5 mL EtOH and 10 mL concentrated HCl. The reaction mixture was stirred for 16 h at rt. Purification by flash chromatography (hexane/EtOAc 7:3 to 4:6) gave the product **2d** as a white solid in a yield of 99% (585 mg). ^{1}H NMR (250 MHz, $CDCl_3$) δ 6.47-6.43 (m, 2H), 6.29 (dt, J = 10.4, 2.1 Hz, 1H), 4.87 (s, 2H).

(3-Amino-2,6-difluorophenyl)methanol (2h)

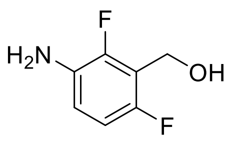

The synthesis was performed according to GP2 using (2,6-Difluoro-3-nitrophenyl)methanol (800 mg, 4.23 mmol, 1.0 eq) and tin(II) chloride dihydrate (3.90 g, 16.9 mmol, 4.0 eq) in 5 mL EtOH and 10 mL concentrated HCl. The reaction mixture was stirred for 16 h at rt. The product **2h** was obtained as a yellow solid in a yield of 87% (585 mg). ^{1}H NMR (250 MHz, $CDCl_3$) δ 6.76-6.36 (m, 2H), 4.76 (s, 2H).

(5-Amino-2,3-difluorophenyl)methanol (2i)

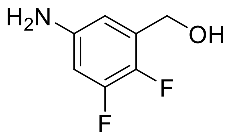

The synthesis was performed according to GP2 using (2,3-difluoro-3-nitrophenyl)methanol (590 mg, 3.12 mmol, 1.0 eq) and tin(II) chloride dihydrate (2.87 g, 12.5 mmol, 4.0 eq) in 5 mL EtOH and 10 mL concentrated HCl. The reaction mixture was stirred for 16 h at rt. The product **2i** was obtained as a brown solid in a yield of 99% (496 mg). ^{1}H NMR (250 MHz, $CDCl_3$) δ 6.48-6.45 (m, 1H), 6.43-6.36 (m, 1H), 4.68 (s, 2H).

(3-Amino-2,5-difluorophenyl)methanol (2j)

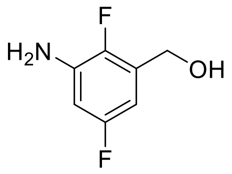

The synthesis was performed according to GP2 using (2,5-difluoro-3-nitrophenyl)methanol (340 mg, 1.80 mmol, 1.0 eq) and tin(II) chloride dihydrate (1.66 g, 7.20 mmol, 4.0 eq) in 5 mL EtOH and 10 mL concentrated HCl. The reaction mixture was stirred for 17 h at rt. The product **2j** was obtained as a brown solid in a yield of 97% (276 mg). ^{1}H NMR (250 MHz, $CDCl_3$) δ 6.51-6.37 (m, 2H), 4.69 (s, 2H).

(3-Amino-2,5,6-trifluorophenyl)methanol (2k)

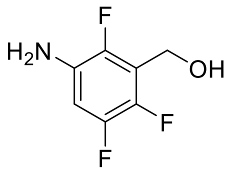

The synthesis was performed according to GP2 using 3-amino-2,5,6-trifluorobenzoic acid (1.0 g, 5.23 mmol, 1.0 eq), sodium borohydride (404 mg, 10.5 mmol, 2.0 eq), and boron trifluoride diethyl etherate (1.74 mL, 14.1 mmol, 2.7 eq) in 16 mL THF. The reaction mixture was stirred for 17 h at rt. The product **2k** was obtained in a yield of 95% (876 mg). ^{1}H NMR (250 MHz, DMSO-d_6) δ 6.72-6.52 (m, 1H), 5.28-5.24 (m, 3H), 4.46 (d, J = 5.5 Hz, 2H).

(3-Amino-6-bromo-2-fluorophenyl)methanol (2l)

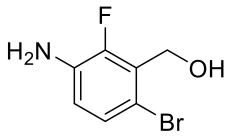

The synthesis was performed according to GP2 using 3-amino-6-bromo-2-fluorobenzoic acid (1.0 g, 4.27 mmol, 1.0 eq), sodium borohydride (330 mg, 8.54 mmol, 2.0 eq), and boron trifluoride diethyl etherate (1.42 mL, 11.5 mmol, 2.7 eq) in 15 mL THF. The reaction mixture was stirred for 17 h at rt. The product **2g** was obtained in a yield of 82% (770 mg). ^{1}H NMR (250 MHz, DMSO-d_6) δ 7.07 (dd,

J = 8.6, 1.5 Hz, 1H), 6.65 (dd, J = 9.3, 8.6 Hz, 1H), 5.27 (br s, 2H), 5.04 (t, J = 5.5 Hz, 1H), 4.52-4.48 (m, 2H).

2,2,2-Trifluoro-N-(3-fluoro-5-(hydroxymethyl)phenyl)acetamide

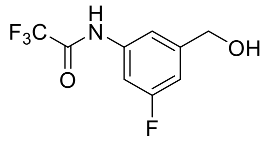

A mixture of 3-amino-5-fluorobenzenemethanol (340 mg, 2.41 mmol), ethyl trifluoroacetate (347 µL, 2.89 mmol) and DMAP (29.7 mg, 0.241 mmol) were dissolved in THF (10.0 mL) and the reaction mixture was stirred at 85°C overnight. The reaction mixture was concentrated under reduced pressure. The residue was dissolved in ethyl acetate and the solution was washed with 2M hydrochloric acid and water. The organic layer was dried over magnesium sulfate, filtered and evaporated to yield the crude product (109 mg, 19% yield) which was used in the next step without further purification. ^{1}H NMR (250 MHz, DMSO-d_6) δ 11.42 (s, 1H), 7.49 (s, 1H), 7.42 (dt, J = 10.7, 2.3 Hz, 1H), 6.99 (dq, J = 9.5, 1.0 Hz, 1H), 5.42 (t, J = 5.7 Hz. 1H), 4.51 (d, J = 5.5 Hz, 2H).

N-(2,4-Difluoro-3-(hydroxymethyl)phenyl)-2,2,2-trifluoroacetamide

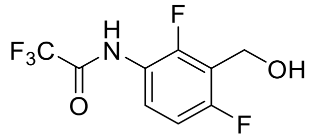

Trifluoroacetic acid anhydride (592 µL, 4.26 mmol) was added dropwise to a solution of **2h** (584 mg, 3.67 mmol), and triethylamine (598 µL, 4.26 mmol) in dichloromethane (15.0 mL) at 0°C. The resulting mixture was stirred at rt overnight. The reaction mixture was diluted with dichloromethane and washed with saturated sodium bicarbonate solution and brine. The organic layer was dried over magnesium sulfate, filtered and evaporated. The crude was purified by flash chromatography (hexane/EtOAc, 9:1 to 5:5) to yield 405 mg (43%) of the desired product. ^{1}H NMR (400 MHz,

DMSO-d_6) δ 11.25 (s, 1H), 7.46 (td, *J* = 8.8, 6.0 Hz, 1H), 7.16 (td, *J* = 9.0, 1.5 Hz, 1H), 5.35 (t, *J* = 5.7 Hz. 1H), 4.52 (d, *J* = 5.6 Hz, 2H).

N-(3-(chloromethyl)phenyl)-2,2,2-trifluoroacetamide (56b/c)

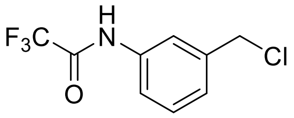

Thionyl chloride (1.15 mL, 35 eq.) was added to a solution of 2,2,2-trifluoro-N-[3-(hydroxmethyl)phenyl]acetamide (100 mg, 0.456 mmol) in dry 1,2-dichloroethane (8.5 mL) and the resulting solution was stirred at 40 °C overnight. The reaction mixture was diluted with dichloromethane and washed with water. The organic layer was dried over magnesium sulfate, filtered and evaporated to yield the crude product (108 mg, 99% yield) which was used in the next step without further purification. ^{1}H NMR (250 MHz, DMSO-d_6) δ 11.32 (br s, 1H), 7.78 (s, 1H), 7.62 (d, *J* = 7.4 Hz, 1H), 7.42 (t, *J* = 7.9 Hz, 1H), 7.29 (d, *J* = 7.5 Hz, 1H), 4.78 (s, 2H).

N-(3-(Chloromethyl)-5-fluorophenyl)-2,2,2-trifluoroacetamide (56d)

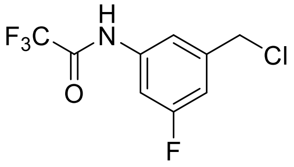

Thionyl chloride (0.928 µL, 30 eq.) was added to a solution of 2,2,2-trifluoro-N-(3-fluoro-5-(hydroxymethyl)phenyl)acetamide (100 mg, 0.422 mmol) in dry 1,2-dichloroethane (10.0 mL) and the resulting solution was stirred at 55 °C overnight. The reaction mixture was diluted with dichloromethane and washed with water. The organic layer was dried over magnesium sulfate, filtered and evaporated to yield the crude product (100 mg, 93% yield) which was used in the next step without further purification. ^{1}H NMR (250 MHz, $CDCl_3$) δ 7.99 (br s, 1H), 7.48 (dt, *J* = 9.7, 2.1 Hz, 1H), 7.34 (s, 1H), 7.00 (dt, *J* = 8.7, 1.6 Hz, 1H), 4.55 (s, 2H).

N-(3-(Chloromethyl)-2,4-difluorophenyl)-2,2,2-trifluoroacetamide (56e/f)

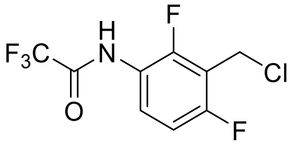

Thionyl chloride (9.0 mL, 30 eq.) was added to a solution of N-(2,4-difluoro-3-(hydroxymethyl)phenyl)-2,2,2-trifluoroacetamide (525 mg, 2.06 mmol) in dry 1,2-dichloroethane (30.0 mL) and the resulting solution was stirred at 40 °C overnight. The reaction mixture was diluted with dichloromethane and washed with water and brine. The organic layer was dried over magnesium sulfate, filtered and evaporated to yield the crude product (527 mg, 94% yield) which was used in the next step without further purification. ^{1}H NMR (400 MHz, DMSO-d_6) δ 11.36 (s, 1H), 7.59 (td, J = 8.8, 6.0 Hz, 1H), 7.27 (td, J = 9.0, 1.5 Hz, 1H), 4.82 (d, 2H).

6-(Chloromethyl)-1H-benzo[d]imidazole (56i)

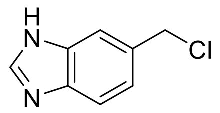

Thionyl chloride (1.70 mL, 35 eq.) was added to a solution of 5-(hydroxymethyl)-1H-benzimidazole (100 mg, 0.675 mmol) in dry 1,2-dichloroethane (8.5 mL) and the resulting solution was stirred at 40 °C overnight. The reaction mixture was diluted with dichloromethane and washed with water. The organic layer was dried over magnesium sulfate, filtered and evaporated to yield the crude product (100 mg, 89% yield) which was used in the next step without further purification. ^{1}H NMR (250 MHz, DMSO-d_6) δ 9.53 (s, 1H), 7.94 (d, J = 1.5 Hz, 1H), 7.85 (d, J = 8.5 Hz, 1H), 7.62 (dd, J = 8.5, 1.5 Hz, 1H), 4.98 (s, 2H).

7-(Chloromethyl)imidazo[1,2-a]pyridine (56j)

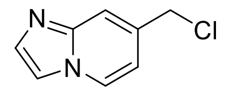

Thionyl chloride (1.70 mL, 35 eq.) was added to a solution of imidazo[1,2-a]pyridine-7-methanol (100 mg, 0.675 mmol) in dry 1,2-dichloroethane (8.5 mL) and the resulting solution was stirred at 40 °C overnight. The reaction mixture was diluted with dichloromethane and washed with water. The organic layer was dried over magnesium sulfate, filtered and evaporated to yield the crude product (112 mg, 99% yield) which was used in the next step without further purification. ^{1}H NMR (250 MHz, DMSO-d_6) δ 8.96 (d, J = 7.0 Hz, 1H), 8.41 (s, 1H), 8.23 (s, 1H), 8.09 (s, 1H), 7.53 (d, J = 7.0 Hz, 1H), 5.02 (s, 2H).

HPLC traces. Conditions**:** Luna 10 µm C18(2) 100 Å, LC Column 250 x 4.6 mm from Phenomenex. Acetonitrile and aqueous formic acid 0.1% were used as eluents. Gradient from 90% to 5% water for 13 min then 7 min 5% water.

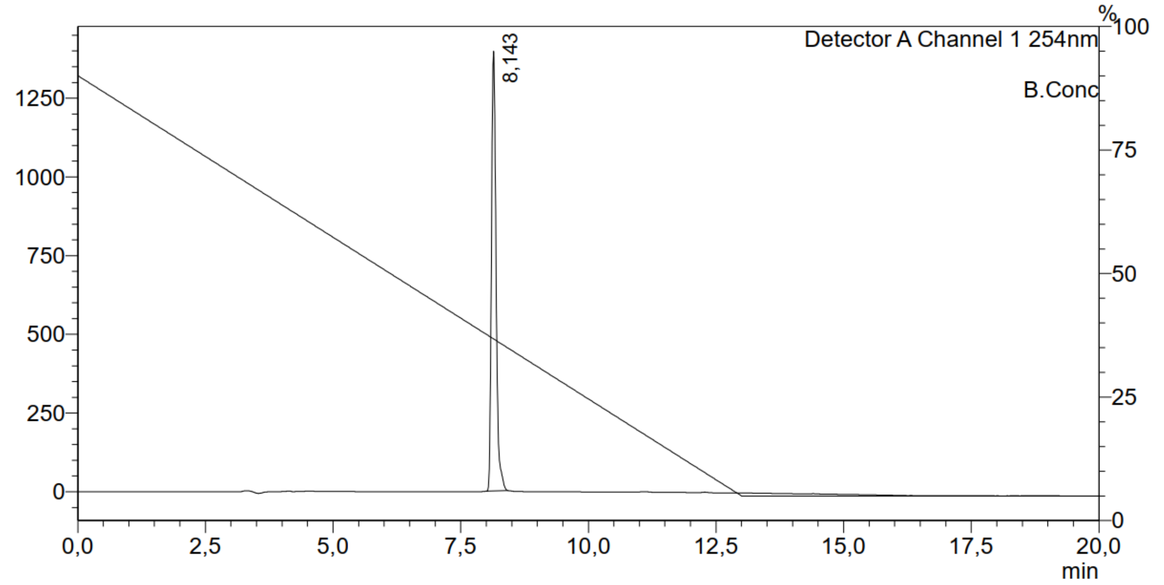

Detector A Channel 1 254nm

Peak#	Ret. Time	Area	Height	Conc.
1	8,143	8743572	1396923	100,000
Total		8743572	1396923	

SI Figure S1: HPLC trace compound **33** (**T-10507**).

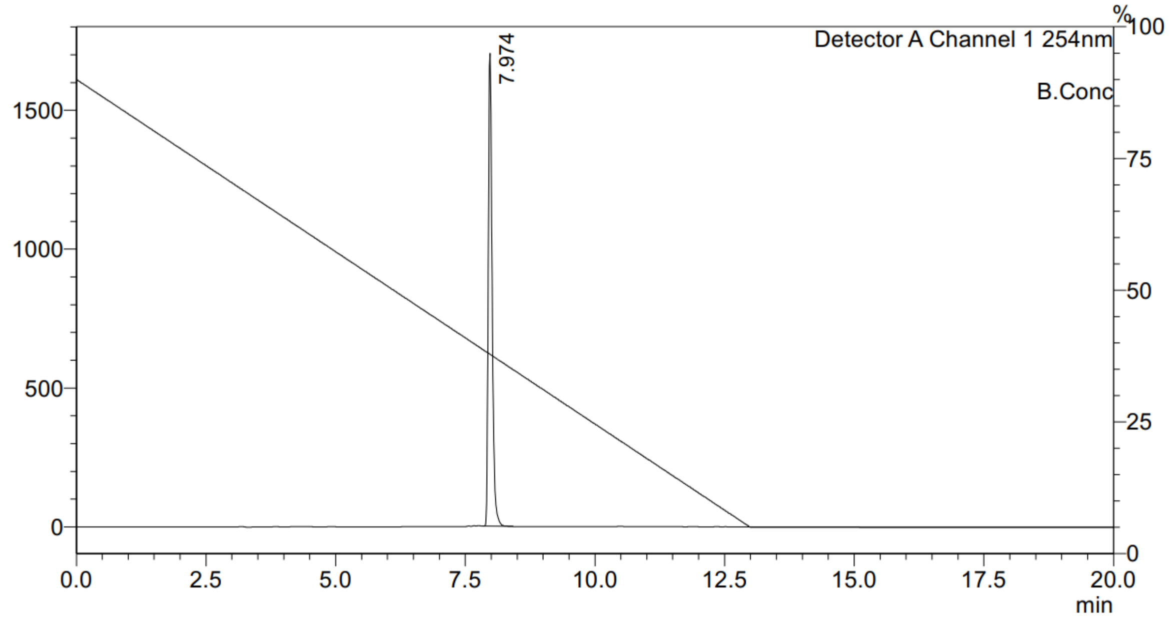

Detector A Channel 1 254nm

Peak#	Ret. Time	Area	Height	Conc.
1	7.974	8871243	1703442	100.000
Total		8871243	1703442	

SI Figure S2: HPLC trace compound **42** (**T-10531**).

SI Table S1: X-ray data collection and refinement statistics.

**Data Collection**	
PDB ID	9SUU
Extended PDB ID	pdb_00009SUU
Beamline	EMBL P13
Space group	I 4_1 3 2
Resolution range (Å)	47.87 - 2.75 (2.85 – 2.75)
Cell dimensions: a, b, c, (Å)	191.483 ,191.48, 191.48
α, β, γ (°)	90, 90, 90
Wavelength (Å)	1.05965
Observed reflections	1274045 (185387)
Unique reflections	15902 (2281)
R_{merge}	0.191 (11.283)
R_{meas}	0.193 (11.353)
Rp.i.m	0.022 (1.253)
CC1/2	0.999 (0.365)
Mean I/σI	19.9 (0.7)
Completeness (%)	100 (100)
Multiplicity	80.1 (81.3)
**Phasing**	
Method	Molecular replacement
**Refinement**	
Resolution (Å)	2.75 - 47.87
Reflections: work/free	14859/1001
R_{work}/R_{free} (%)	22.0/24.5
**Number of Atoms**	
Protein	2788
Water	8
Non-water solvent	16
Inhibitor	40
**Mean B Factors**	
Wilson B (Å^2)	119.57
Protein (Å^2)	119.61
Water (Å^2)	89.91
Non-water solvent (Å^2)	123.54
Inhibitor (Å^2)	120.67
**RMSD**	
Bond lengths (Å)	0.0021
Bond angles (°)	0.45
Ramachandran statistics: favoured/allowed/outliers (%)	95.21/4.79/0.00

Numbers in parentheses correspond to the highest resolution shell.

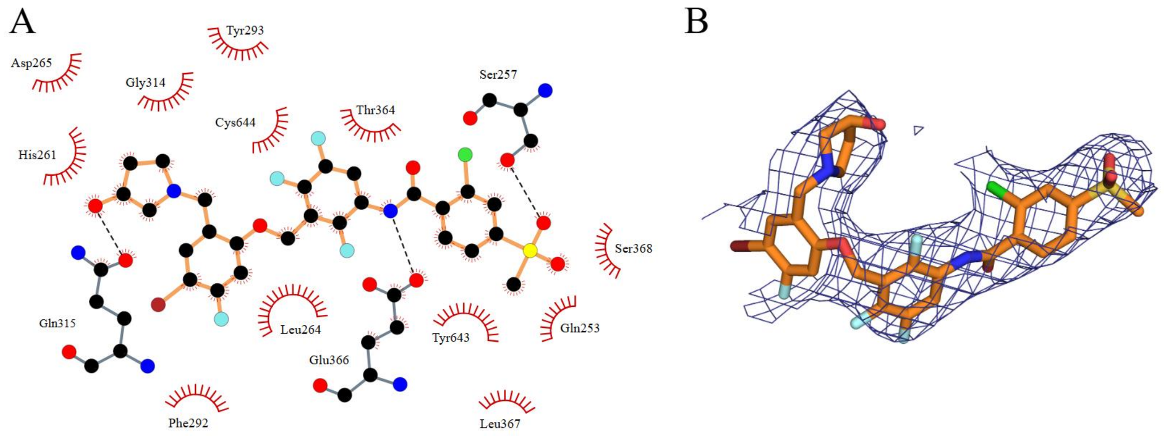

SI Figure S3: Detail of T-10531 binding site and X-ray density

A) Ligand-protein interaction schematic of the T-10531 binding site in USP28, generated with LIGPLOT. Hydrogen bonds are depicted as black dashed lines. Hydrophobic contacts are indicated by red spoked arcs.
B) 2mFo-DFc simulated annealing composite omit map (1σ level) of the density of inhibitor T-10531 (blue mesh) within the USP28-T-10531 structure (T-10531, orange stick representation).

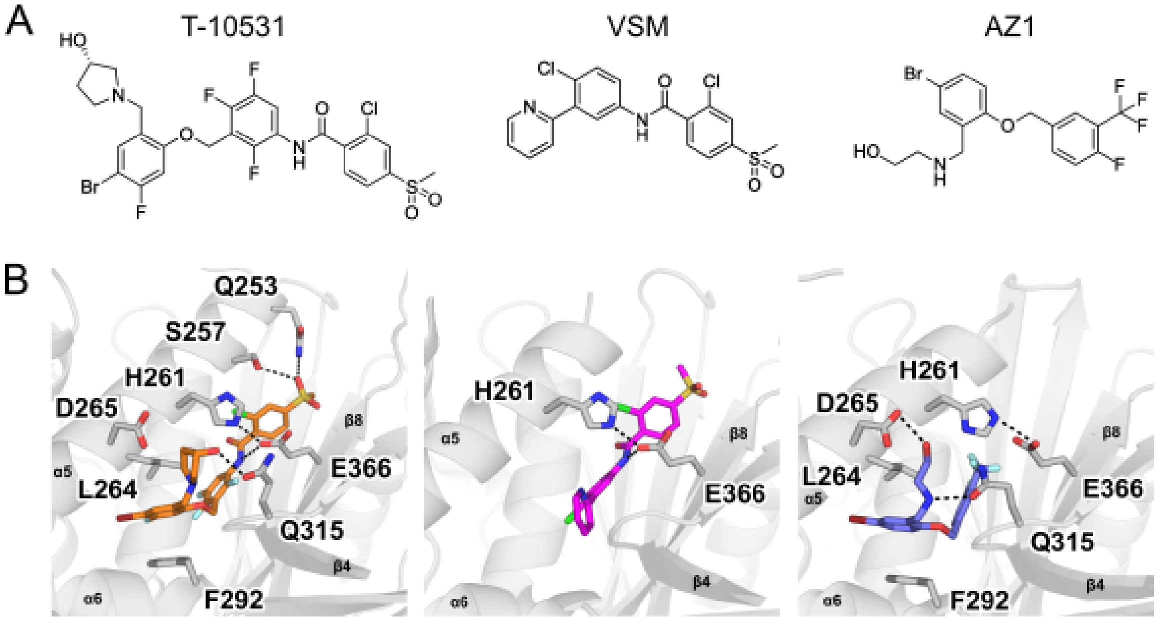

SI Figure S4: Comparison of T-10531, Vismodegib, and AZ1 binding to USP28.

A) Skeletal structural formulae of T-10531 (left), VSM (centre), and AZ1 (right).
B) Detail view of USP28 bound to T-10531 (left, orange). For comparison, USP28:VSM (centre, magenta, PDB-ID: 8P14), and USP28:AZ1 (right, blue, PDB-ID: 8P1P) structures are provided (Patzke et al. 2024). USP28 is depicted as grey ribbons, with key sidechains shown in stick representation. Hydrogen bonds are depicted as black dashed lines.

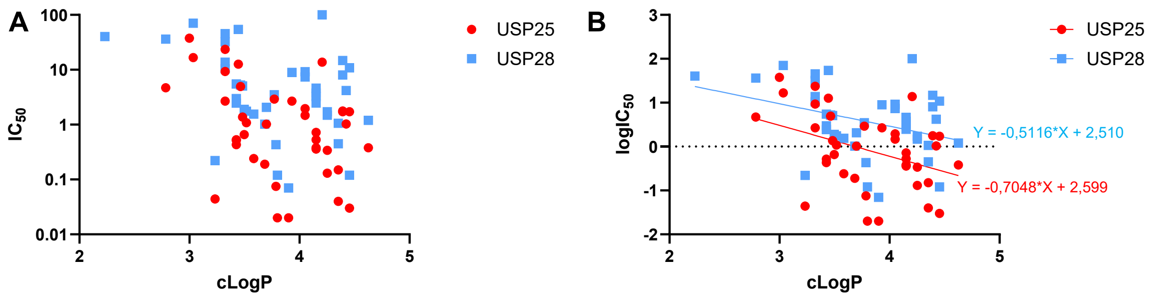

SI Figure S5: Correlation of clogP and the inhibitory potency of all compounds presented in this study (compounds with IC50>100 µM were considered as inactive and omitted from the analysis). A. Correlation between IC50 and clogP. **B.** Linear regression of logIC50 values in correlation to clogP.
